# Effectiveness of Bariatric Surgery vs Community Weight Management Intervention for the Treatment of Idiopathic Intracranial Hypertension: A Randomized Clinical Trial

**DOI:** 10.1001/jamaneurol.2021.0659

**Published:** 2021-04-26

**Authors:** Susan P. Mollan, James L. Mitchell, Ryan S. Ottridge, Magda Aguiar, Andreas Yiangou, Zerin Alimajstorovic, David M. Cartwright, Olivia Grech, Gareth G. Lavery, Connar S. J. Westgate, Vivek Vijay, William Scotton, Ben R. Wakerley, Tim D. Matthews, Alec Ansons, Simon J. Hickman, James Benzimra, Caroline Rick, Rishi Singhal, Abd A. Tahrani, Kristian Brock, Emma Frew, Alexandra J. Sinclair

**Affiliations:** 1 Birmingham Neuro-Ophthalmology, University Hospitals Birmingham NHS Foundation Trust, Queen Elizabeth Hospital, Birmingham, United Kingdom; 2 Department of Metabolic Neurology, Institute of Metabolism and Systems Research, College of Medical and Dental Sciences, University of Birmingham, Birmingham, United Kingdom; 3 Department of Neurology, University Hospitals Birmingham NHS Foundation Trust, Queen Elizabeth Hospital, Birmingham, United Kingdom; 4 Birmingham Clinical Trials Unit, College of Medical and Dental Sciences, University of Birmingham, Birmingham, United Kingdom; 5 Health Economics Unit, Institute of Applied Health Research, University of Birmingham, Birmingham, United Kingdom; 6 Centre for Endocrinology, Diabetes and Metabolism, Birmingham Health Partners, Birmingham, United Kingdom; 7 Institute of Metabolism and Systems Research, College of Medical and Dental Sciences, University of Birmingham, Birmingham, United Kingdom; 8 Department of Neurology, Gloucestershire Hospitals NHS Foundation Trust, Gloucester, United Kingdom; 9 Manchester University NHS Foundation Trust, Manchester Royal Eye Hospital, Manchester, United Kingdom; 10 Department of Neurology, Royal Hallamshire Hospital, Sheffield, United Kingdom; 11 Department of Ophthalmology, Royal Devon and Exeter NHS Foundation Trust, Exeter, United Kingdom; 12 Nottingham Clinical Trials Unit, University of Nottingham, University Park, Nottingham, United Kingdom; 13 Upper GI Unit and Minimally Invasive Unit, Birmingham Heartlands Hospital, University Hospitals Birmingham NHS Foundation Trust, Birmingham, United Kingdom; 14 Institute of Cancer and Genomic Sciences, University of Birmingham, Birmingham, United Kingdom; 15 Department of Endocrinology, University Hospitals Birmingham NHS Foundation Trust, Queen Elizabeth Hospital, Birmingham, United Kingdom; 16 Cancer Research UK Clinical Trials Unit, University of Birmingham, Birmingham, United Kingdom

## Abstract

**Importance:**

Idiopathic intracranial hypertension (IIH) causes headaches, vision loss, and reduced quality of life. Sustained weight loss among patients with IIH is necessary to modify the disease and prevent relapse.

**Objective:**

To compare the effectiveness of bariatric surgery with that of a community weight management (CWM) intervention for the treatment of patients with active IIH.

**Design, Setting, and Participants:**

This 5-year randomized clinical trial (Idiopathic Intracranial Hypertension Weight Trial) enrolled women with active IIH and a body mass index (calculated as weight in kilograms divided by height in meters squared) of 35 or higher at 5 National Health Service hospitals in the UK between March 1, 2014, and May 25, 2017. Of 74 women assessed for eligibility, 6 did not meet study criteria and 2 declined to participate; 66 women were randomized. Data were analyzed from November 1, 2018, to May 14, 2020.

**Interventions:**

Bariatric surgery (n = 33) or CWM intervention (Weight Watchers) (n = 33).

**Main Outcomes and Measures:**

The primary outcome was change in intracranial pressure measured by lumbar puncture opening pressure at 12 months, as assessed in an intention-to-treat analysis. Secondary outcomes included lumbar puncture opening pressure at 24 months as well as visual acuity, contrast sensitivity, perimetric mean deviation, and quality of life (measured by the 36-item Short Form Health Survey) at 12 and 24 months. Because the difference in continuous outcomes between groups is presented, the null effect was at 0.

**Results:**

Of the 66 female participants (mean [SD] age, 32.0 [7.8] years), 64 (97.0%) remained in the clinical trial at 12 months and 54 women (81.8%) were included in the primary outcome analysis. Intracranial pressure was significantly lower in the bariatric surgery arm at 12 months (adjusted mean [SE] difference, −6.0 [1.8] cm cerebrospinal fluid [CSF]; 95% CI, −9.5 to −2.4 cm CSF; *P* = .001) and at 24 months (adjusted mean [SE] difference, −8.2 [2.0] cm CSF; 95% CI, −12.2 to −4.2 cm CSF; *P* < .001) compared with the CWM arm. In the per protocol analysis, intracranial pressure was significantly lower in the bariatric surgery arm at 12 months (adjusted mean [SE] difference, −7.2 [1.8] cm CSF; 95% CI, −10.6 to −3.7 cm CSF; *P* < .001) and at 24 months (adjusted mean [SE] difference, −8.7 [2.0] cm CSF; 95% CI, −12.7 to −4.8 cm CSF; *P* < .001). Weight was significantly lower in the bariatric surgery arm at 12 months (adjusted mean [SE] difference, −21.4 [5.4] kg; 95% CI, −32.1 to −10.7 kg; *P* < .001) and at 24 months (adjusted mean [SE] difference, −26.6 [5.6] kg; 95% CI, −37.5 to −15.7 kg; *P* < .001). Quality of life was significantly improved at 12 months (adjusted mean [SE] difference, 7.3 [3.6]; 95% CI, 0.2-14.4; *P* = .04) and 24 months (adjusted mean [SE] difference, 10.4 [3.8]; 95% CI, 3.0-17.9; *P* = .006) in the bariatric surgery arm.

**Conclusions and Relevance:**

In this randomized clinical trial, bariatric surgery was superior to a CWM intervention in lowering intracranial pressure. The continued improvement over the course of 2 years shows the impact of this intervention with regard to sustained disease remission.

**Trial Registration:**

ClinicalTrials.gov Identifier: NCT02124486

## Key Points

**Question:**

Is bariatric surgery superior to a community weight management intervention in sustaining the weight loss necessary to achieve sustained remission among patients with idiopathic intracranial hypertension?

**Findings:**

In this randomized clinical trial of 66 women with idiopathic intracranial hypertension and a body mass index of 35 or higher, bariatric surgery was superior to a community weight management intervention in decreasing intracranial pressure, with continued improvement at 2 years.

**Meaning:**

The study’s findings indicate that, among women with idiopathic intracranial hypertension and a body mass index of 35 or higher, bariatric surgery is an effective treatment to reduce intracranial pressure and for sustained disease remission.

## Teaser

This randomized clinical trial examines the efficacy of bariatric surgery compared with a community weight management intervention for the treatment of patients with idiopathic intracranial hypertension.

## Introduction

Idiopathic intracranial hypertension (IIH) is a debilitating condition characterized by increased intracranial pressure (ICP) that causes optic disc swelling known as papilledema, with a risk of permanent visual loss and chronic headaches that lead to reduced quality of life.^1,2^ The condition predominately affects women aged 25 to 36 years, with weight gain being a major risk factor.^3,4,5^

The incidence of IIH is increasing and has been associated with increasing obesity rates worldwide.^3,4^ Modest weight gain (approximately 5%) has been associated with an increased risk of developing IIH and experiencing relapses of the disease.^5^ Weight loss has been reported to be a beneficial treatment strategy, with a reduction in body weight of 3% to 15% associated with disease remission as defined by ICP normalization and papilledema resolution.^6^ Body weight is the main modifiable factor associated with the development of IIH,^7^ and a patient-physician priority partnership has emphasized the importance of conducting research to evaluate the most effective approach to treating patients with IIH through weight loss interventions.^8^

Community weight management interventions (excluding very low-energy diets) have been associated with modest weight loss (approximately 5%).^9^ A previous study^6^ reported that a very low-energy diet (≤425 kcal per day) for 3 months was associated with weight loss of 15%, reductions in ICP, improvements in papilledema and visual function, and decreases in headache frequency and severity, with concomitant reductions in the use of analgesic medications. Maintaining weight loss is difficult, and most patients regain weight over a 2- to 5-year period.^10^ Bariatric surgery has been associated with sustained long-term weight loss (25%-30%) as well as positive cardiovascular and metabolic outcomes.^11,12^ Case series suggest that bariatric surgery is also associated with remission among patients with IIH, with concomitant improvement in symptoms and discontinuation of medication, but to our knowledge, there is currently no evidence from randomized clinical trials.^13,14^

We hypothesized that bariatric surgery would be superior to a community weight management intervention in reducing ICP among patients with IIH because of greater sustained weight loss. We therefore conducted a multicenter randomized clinical trial (Idiopathic Intracranial Hypertension Weight Trial [IIH:WT]) comparing bariatric surgery with a community weight management intervention to evaluate which approach was more effective in decreasing ICP among participants with active IIH, with the primary end point being lumbar puncture (LP) opening pressure measured after 12 months.

## Methods

### Trial Design and Participants

The IIH:WT was a 5-year multicenter parallel-group randomized clinical trial (NCT02124486) (trial protocol in Supplement 1). We recruited participants at 5 National Health Service hospitals in the UK between March 1, 2014, and May 25, 2017. Participants were identified from the neurology and ophthalmology clinics of 7 National Health Service hospitals (eMethods 1 in Supplement 2). The National Research Ethics Committee of West Midlands approved the clinical trial, and the trial protocol was reported before enrollment was completed.^15^ All participants provided written informed consent. This study followed the Consolidated Standards of Reporting Trials (CONSORT) reporting guideline for randomized clinical trials.

### Participants

We recruited women aged 18 to 55 years who met the diagnostic criteria for IIH^16^; had normal results from brain imaging, including magnetic resonance venography or computed tomographic venography (apart from radiological signs of increased ICP); had a body mass index (BMI) (calculated as weight in kilograms divided by height in meters squared) of 35 or higher; and had not succeeded in losing weight or maintaining weight loss. To be classified as having active disease, participants were required to have a baseline LP opening pressure of 25 cm cerebrospinal fluid (CSF) or higher and to have papilledema at baseline. Detailed inclusion and exclusion criteria are provided in eTable 1 in Supplement 2.

### Randomization and Treatment

Participants were randomized in a 1:1 ratio to receive either a community weight management intervention (Weight Watchers; weight management arm) or bariatric surgery (surgery arm) using computer-generated random numbers and were stratified by use vs nonuse of acetazolamide medication (eMethods 2 in Supplement 2). All assessors were masked to treatment allocation. A complete medical history, clinical measurements, and a headache diary were completed by all participants in accordance with the study protocol (Supplement 1).^15^

### Outcome Measures

The primary outcome was the difference in ICP between the surgery arm and the weight management arm as measured by LP opening pressure at 12 months. Secondary outcomes included LP opening pressure at 24 months, visual acuity (logMAR; measured using Early Treatment Diabetic Retinopathy Study testing charts), contrast sensitivity (assessed using the Mars Letter Contrast Sensitivity Test), perimetric mean deviation (central threshold automated perimetry measured using the Humphrey 24-2 Swedish interactive thresholding algorithm), and health-associated quality of life (measured using the 36-item Short Form Health Survey,^17^ the Hospital Anxiety and Depression Scale,^18^ and the 5-level EuroQol 5-Dimension questionnaire^14^). Evaluations were performed at baseline and at 3, 6, 12, and 24 months and were planned for 60 months.

Optic nerve head swelling was assessed using spectral domain optical coherence tomography (Spectralis; Heidelberg Engineering). Three neuroophthalmologists (J.B., T.D.M., and S.P.M.) who were masked to participant identity graded papilledema from color fundus photographs (Topcon Medical) using Frisén classification.^19^ Headache symptoms were evaluated using the 6-item Headache Impact Test,^20^ symptom severity scores (rating scale, 0 to 10, with 0 indicating no pain and 10 indicating maximum pain), symptom frequency (days per month), and analgesic medication use (days per month). Any adverse events or serious adverse events (SAEs) that occurred were documented.

### Sample Size Calculation

In a previous study of weight loss among patients with IIH who followed a low-energy diet for 3 months, LP opening pressure was found to be significantly reduced by a mean (SD) of 8 (4.2) cm CSF (*P* < .001), with mean (SD) weight loss of 15.3% (7.0%) of body weight.^6^ We inferred that a similar reduction of LP opening pressure of 8 cm CSF would occur in the surgery arm and that a smaller reduction of 3 cm CSF would occur in the weight management arm (a value to reflect changes slightly greater than the baseline fluctuations observed in the previous study^6^).

We therefore planned to detect a mean difference of 5.0 cm CSF between the groups with 90% power and an error rate of α = .05 using a 2-sided *t* test (assuming an SD of 5.1 cm CSF),^15^ which would have required a sample of 46 patients (23 patients per arm). To allow for a 28% withdrawal rate, 32 participants per arm were required. Based on these assumptions, 66 women (33 participants per arm) were recruited.

### Statistical Analysis

All primary analyses (primary and secondary outcomes, including safety outcomes) were evaluated using intention-to-treat analysis. A per protocol analysis was also performed for the primary outcome as part of a planned secondary analysis. For the per protocol analysis, the surgery arm was defined as participants who had undergone bariatric surgery within 12 months of randomization, and the weight management arm was defined as participants who had not undergone bariatric surgery within 12 months of randomization. Analysis was completed using received data only, with effort made to follow up with participants even after instances of protocol nonadherence to minimize the potential for bias. No imputation of missing data was conducted. The analysis of visual data included data from both eyes, with data on the more affected eye at baseline (defined by perimetric mean deviation) being reported.

Statistical analysis was performed using R software, version 3.6.3 (R Foundation for Statistical Computing). Data were reported as means, SDs or SEs (with medians and interquartile ranges [IQRs] used for nonnormal data), and 95% CIs, as appropriate. Hierarchical linear regression models were used to analyze repeated measures of the primary and secondary outcomes and to estimate differences adjusted for baseline values (eMethods 2 and eTable 2 in Supplement 2). In these models, population-level effects (ie, fixed effects) included the intercept, time (as a factor variable), and the 2-way interaction of treatment arm and time (as a factor variable) to model differences in treatment effects over time. Group-level effects (ie, random effects) comprised patient-level adjustments to the intercept. Because the difference in continuous outcomes between groups is presented, the null effect was at 0. The threshold for statistical significance was prespecified at *P* = .05. Data were analyzed from November 1, 2018, to May 14, 2020.

## Results

### Participants

**Figure 1.**
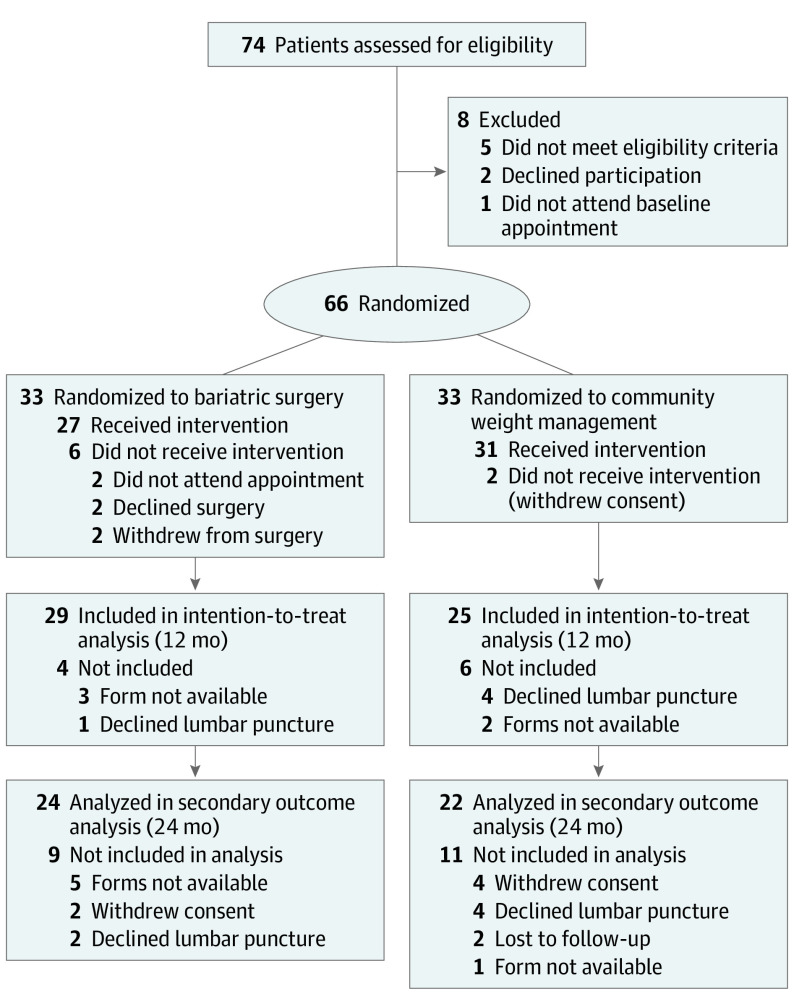
CONSORT Diagram In the bariatric surgery arm, 18 patients were assessed 2 weeks after surgery.

Between 2014 and 2017, 74 women were assessed for eligibility; 6 women did not meet study criteria, and 2 women declined to participate. A total of 66 women (mean [SD] age, 32.0 [7.8] years) enrolled in the study and were randomly assigned to either the surgery arm (n = 33) or the weight management arm (n = 33) (Figure 1). The study population had a mean (SD) LP opening pressure of 35.5 (7.0) cm CSF, and the clinical trial arms were balanced with regard to baseline characteristics (Table 1).

**Table 1.** Baseline Characteristics of Participants in the Trial

Characteristic	Participants^a^		
	All (N = 66)	Bariatric surgery arm (n = 33)	CWM intervention arm (n = 33)
Age, mean (SD), y	32.0 (7.8)	31.0 (8.0)	33.0 (7.7)
Female	66 (100)	33 (100)	33 (100)
Race/ethnicity			
White	55 (83.3)	27 (81.8)	28 (84.8)
Mixed or multiple	5 (7.6)	3 (9.1)	2 (6.1)
Black, African, or Caribbean	5 (7.6)	3 (9.1)	2 (6.1)
Asian or British Asian	1 (1.5)	0	1 (3.0)
Duration of IIH diagnosis, median (range), y	1.1 (0.5-2.6)	1.1 (0.6-2.7)	0.8 (0.4-2.5)
Frisén grade of worse eye, mean (SD)	2.1 (1.0)	2.0 (0.9)	2.2 (1.1)
Perimetric mean deviation of worse eye			
Mean (SD), dB	−3.6 (3.7)	−3.6 (3.5)	−3.5 (3.8)
Participants, No.	65	32	33
LP opening pressure at diagnosis			
Mean (SD), cm CSF	35.5 (7.0)	34.5 (5.7)	36.5 (8.0)
Participants, No.	60	30	30
LP opening pressure at baseline, mean (SD), cm CSF	34.7 (5.7)	34.8 (5.8)	34.6 (5.6)
Acetazolamide receipt	19 (28.8)	8 (24.2)	11 (33.3)

Abbreviations: CSF, cerebrospinal fluid; CWM, community weight management; IIH, idiopathic intracranial hypertension; LP, lumbar puncture.

^a^ Data are presented as number (percentage) of participants unless otherwise indicated.

### Adherence to Protocol

A total of 64 women (97.0%) remained in the clinical trial at 12 months, and 54 women (81.8%) completed the primary outcome. Six participants in the surgery arm did not receive bariatric surgery based on personal choice, and no participants were medically declined for surgery. Two participants withdrew from the weight management arm; between 12 and 24 months, 2 additional participants in the weight management arm underwent bariatric surgery (on a self-funded basis) (Figure 1).

### Treatments

In the surgery arm, the median time from randomization to bariatric surgery was 4.4 months (range, 2.2-10.3 months). Among the 27 participants who underwent surgery, the predominant procedure was Roux-en-Y gastric bypass (12 participants [44.4%]) followed by gastric banding (10 participants [37.0%]) and laparoscopic sleeve gastrectomy (5 participants [18.5%]). Among those in the weight management arm, the mean (SD) number of Weight Watchers face-to-face sessions attended was 14.3 (10.6), with 19 of 33 participants (57.6%) attending at least 1 session.

### Primary Outcomes

**Figure 2.**
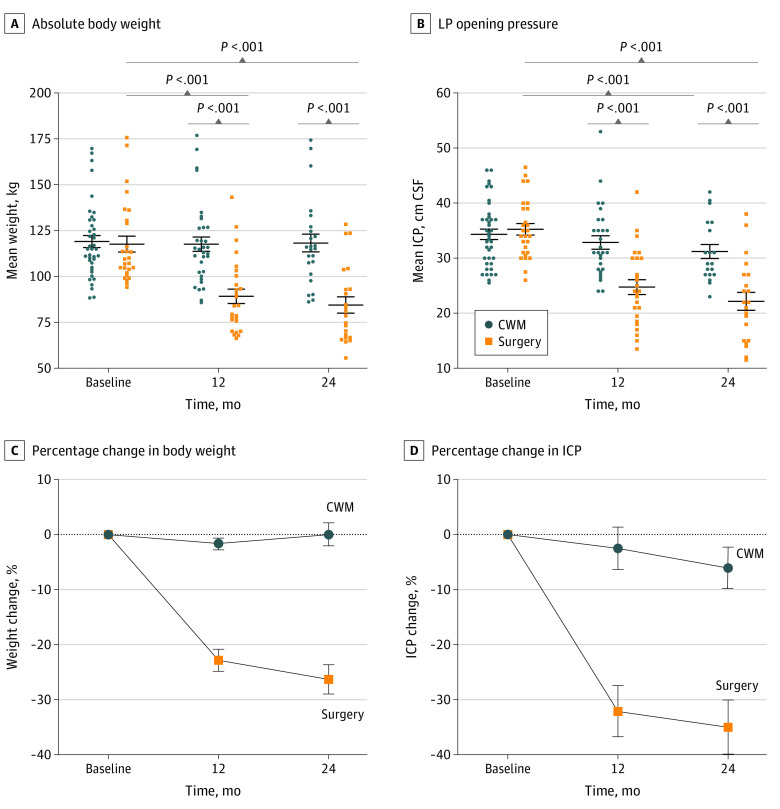
Body Weight, Lumbar Puncture (LP) Opening Pressure, Percentage Change in Body Weight, and Percentage Change in Intracranial Pressure (ICP) by Trial Arm Whiskers represent SEs. CSF indicates cerebrospinal fluid; CWM, community weight management.

The mean (SD) LP opening pressure decreased from 34.8 (5.8) cm CSF at baseline to 26.4 (8.7) cm CSF at 12 months (adjusted mean [SE] difference, −8.7 [1.3] cm CSF; 95% CI, −11.3 to −6.1 cm CSF; *P* < .001) in the surgery arm and from 34.6 (5.6) cm CSF at baseline to 32.0 (5.2) cm CSF at 12 months (adjusted mean [SE] difference, −2.5 [1.4] cm CSF; 95% CI, −5.2 to 0.3 cm CSF; *P* = .08) in the weight management arm, but the difference for the latter was not significant (Table 2 and Figure 2). The prespecified primary outcome analysis indicated that the adjusted mean (SE) difference in LP opening pressure was −6.0 (1.8) cm CSF (95% CI, −9.5 to −2.4 cm CSF; *P* = .001) between the groups at 12 months.

**Table 2.** Primary Outcome and Anthropometric Features by Treatment Arm

Outcome or feature	Baseline		At surgery		At 2 wk after surgery		At 12 mo		At 24 mo		Difference from baseline to 12 mo			Difference from baseline to 24 mo			Difference between arms at 12 mo			Difference between arms at 24 mo		
	Mean (SD)	Participants, No.	Mean (SD)	Participants, No.	Mean (SD)	Participants, No.	Mean (SD)	Participants, No.	Mean (SD)	Participants, No.	Hierarchical regression		*P* value	Hierarchical regression		*P* value	Hierarchical regression		*P* value	Hierarchical regression		*P* value
											Mean (SE)	95% CI		Mean (SE)	95% CI		Mean (SE)	95% CI		Mean (SE)	95% CI	
ICP (intention to treat), cm CSF																						
CWM intervention	34.6 (5.6)	33	NA	NA	NA	NA	32.0 (5.2)	25	31.0 (5.7)	18	−2.5 (1.4)	−5.2 to 0.3	.08	−3.5 (1.6)	−6.6 to −0.3	.03	−6.0 (1.8)	−9.5 to −2.4	.001	−8.2 (2.0)	−12.2 to −4.2	<.001
Bariatric surgery	34.8 (5.8)	33	NA	NA^a^	26.9 (8.1)	18	26.4 (8.7)	29	22.8 (7.8)	22	−8.7 (1.3)	−11.3 to −6.1	<.001	−11.9 (1.5)	−14.8 to −9.0	<.001						
ICP (per protocol), cm CSF																						
CWM intervention	34.6 (5.9)	33	NA	NA	NA	NA	32.4 (6.5)	26	31.4 (5.9)	17	−1.9 (1.4)	−4.6 to 0.7	.15	−3.0 (1.6)	−6.1 to 0.1	.06	−7.2 (1.8)	−10.6 to −3.7	<.001	−8.7 (2.0)	−12.7 to −4.8	<.001
Bariatric surgery	34.9 (5.4)	30	NA	NA^a^	NA	NA	25.7 (7.5)	28	22.8 (7.4)	23	−9.4 (1.3)	−12.1 to −6.8	<.001	−12.1 (1.4)	−14.9 to −9.3	<.001						
Weight, kg																						
CWM intervention	118.5 (20.7)	33	NA	NA	NA	NA	116.6 (22.3)	29	116.5 (22.9)	22	−2.1 (2.0)	−6.0 to 1.8	.29	−1.4 (2.2)	−5.6 to 2.9	.53	−21.4 (5.4)	−32.1 to −10.7	<.001	−26.6 (5.6)	−37.5 to −15.7	<.001
Bariatric surgery	118.4 (21.8)	33	113.3 (21.7)	27	102.3 (18.8)	18	94.0 (23.7)	30	88.9 (25.9)	24	−23.4 (1.9)	−27.2 to −19.6	<.001	−27.8 (2.1)	−31.9 to −23.8	<.001						
Excess body weight, kg																						
CWM intervention	50.6 (19.4)	33	NA	NA	NA	NA	49.1 (21.3)	29	49.5 (22.1)	22	−1.9 (1.9)	−5.7 to 1.9	.32	−1.3 (2.1)	−5.5 to 2.8	.53	−20.3 (5.1)	−30.3 to −10.2	<.001	−25.8 (5.3)	−36.1 to −15.5	<.001
Bariatric surgery	51.5 (20.0)	33	46.2 (20.0)	27	36.5 (16.4)	18	27.2 (22.3)	30	21.2 (24.9)	24	−23.0 (1.9)	−26.8 to −19.3	<.001	−28.0 (2.1)	−32.0 to −23.9	<.001						
BMI																						
CWM intervention	43.7 (7.1)	33	NA	NA	NA	NA	43.1 (7.8)	29	43.5 (8.0)	22	−0.7 (0.7)	−2.1 to 0.7	.35	−0.4 (0.8)	−1.9 to 1.2	.62	−7.3 (1.9)	−11.0 to −3.7	<.001	−9.4 (1.9)	−13.2 to −5.7	<.001
Bariatric surgery	44.2 (7.1)	33	42.2 (7.1)	27	38.9 (5.7)	18	35.1 (8.0)	30	32.8 (8.9)	24	−8.5 (0.7)	−9.9 to −7.2	<.001	−10.4 (0.8)	−11.8 to −8.9	<.001						

Abbreviations: BMI, body mass index (calculated as weight in kilograms divided by height in meters squared); CSF, cerebrospinal fluid; CWM, community weight management; ICP, intracranial pressure; NA, not applicable.

^a^ Intracranial pressure was not assessed until 2 weeks after surgery was performed.

### Secondary Outcomes

The secondary outcome of LP opening pressure at 24 months demonstrated increasing effect size between 12 and 24 months, with a mean (SE) difference between the 2 arms of −8.2 (2.0) cm CSF (95% CI, −12.2 to −4.2 cm CSF; *P* < .001). In the per protocol analysis, ICP was significantly lower in the surgery arm at 12 months (adjusted mean [SE] difference, −7.2 [1.8] cm CSF; 95% CI, −10.6 to −3.7 cm CSF; *P* < .001) and at 24 months (adjusted mean [SE] difference, −8.7 [2.0] cm CSF; 95% CI, −12.7 to −4.8 cm CSF; *P* < .001) (Table 2). Exploratory analysis showed that the mean (SE) ICP had decreased in the surgery arm from 34.8 (5.8) cm CSF at baseline to 26.9 (8.1) cm CSF at 2 weeks after surgery (*P* < .001) (Table 2 and Figure 2). At 12 months, the mean (SE) percentage change in ICP was −32.1% (4.7%) in the surgery arm compared with −2.5% (3.9%) in the weight management arm (*P* < .001). At 24 months, the mean (SE) percentage change in ICP was −35.0% (4.9%) in the surgery arm compared with −6.0% (3.8%) in the weight management arm (*P* < .001) (Figure 2).

At both 12 and 24 months, all measures of improvement in weight, BMI, and reduction of excess body weight were significantly greater in the surgery arm vs the weight management arm (*P* < .001) (Table 2 and Figure 2), with increased effect between 12 and 24 months. Weight was significantly lower in the surgery arm at 12 months (adjusted mean [SE] difference, −21.4 [5.4] kg; 95% CI, −32.1 to −10.7 kg; *P* < .001) and at 24 months (adjusted mean [SE] difference, −26.6 [5.6] kg; 95% CI, −37.5 to −15.7 kg; *P* < .001) compared with the weight management arm. With regard to the percentage of weight loss (Figure 2) and the percentage of excess weight loss, the mean (SE) difference between groups at 12 months was −18.3% (1.9%; 95% CI, −22.1% to −14.6%; *P* < .001) and −46.4% (4.9%; 95% CI, −56.1% to −36.7%; *P* < .001), respectively. The 24-month results were similar (adjusted mean [SE] difference in weight loss, −23.6% [2.1%; 95% CI, −27.8% to −19.4%]; adjusted mean [SE] difference in excess weight loss, −61.6% [5.5%; 95% CI, −72.3% to −50.8%]; *P* < .001 for both) (Figure 2).

Papilledema was reduced in both arms; from baseline to 12 months, the median Frisén grade decreased from 2 (IQR, 2-3) to 1 (IQR, 1-2) in the surgery arm and from 2 (IQR, 2-3) to 1 (IQR, 1-2) in the weight management arm. Differences in headache disability, as measured by Headache Impact Test scores between the 2 arms, were not significant at 12 months (adjusted mean [SE] difference, −1.4 [2.6]; 95% CI, −6.6 to 3.8; *P* = .60) or 24 months (adjusted mean [SE] difference, −1.4 [2.8]; 95% CI, −7.0 to 4.1; *P* = .61) (eTable 3 in Supplement 2). Exploratory analysis indicated a greater improvement in mean monthly headache days, headache severity, and Headache Impact Test scores in the surgery arm between baseline and 12 months (eTable 3 in Supplement 2). Differences in visual function, as measured by perimetric mean deviation between the 2 arms, were not significant at 12 months (adjusted mean [SE] difference, −0.5 [0.8]; 95% CI, −2.0 to 1.0; *P* = .53) or 24 months (adjusted mean [SE] difference, −0.1 [0.8]; 95% CI, −1.5 to 1.8; *P* = .86) (eTable 4 in Supplement 2). There was no evidence of improvement in IIH symptoms in either group (eTable 5 in Supplement 2).

Analysis of quality of life using the 36-item Short Form Health Survey showed a significant change in the physical component score at 12 months (adjusted mean [SE] difference, 7.3 [3.6]; 95% CI, 0.2-14.4; *P* = .04). This change was also significant at 24 months between the 2 arms (adjusted mean [SE] difference, 10.4 [3.8]; 95% CI, 3.0-17.9; *P* = .006) (eTable 6 in Supplement 2). In addition, significant improvement was observed in the 3 domains of energy and fatigue at 12 months (adjusted mean [SE] difference, 14.9 [6.4]; 95% CI, 2.4-27.4; *P* = .02), in physical functioning at both 12 months (adjusted mean [SE] difference, 20.2 [6.8]; 95% CI, 6.9-33.5; *P* = .003) and 24 months (adjusted mean [SE] difference, 27.7 [7.2]; 95% CI, 13.7-41.8; *P* < .001), and in general health at 24 months (adjusted mean [SE] difference, 22.8 [6.0]; 95% CI, 11.1-34.6; *P* < .001) (eTable 7 in Supplement 2). No other domains showed significant differences (eTable 7 in Supplement 2). Scores from the Hospital Anxiety and Depression Scale showed within-arm improvement in the surgery arm, with changes in scores on the depression subscale at 12 months (adjusted mean [SE] difference, −1.6 [0.8]; 95% CI, −3.1 to 0; *P* = .05) and 24 months (adjusted mean [SE] difference, −2.7 [0.9]; 95% CI, −4.4 to −1.0; *P* = .002) (eTable 6 in Supplement 2). Relevant medication changes over the course of the clinical trial are available in the eResults in Supplement 2.

### Adverse Events

In the whole cohort, 15 SAEs were reported by 12 months, and an additional 9 SAEs were reported by 24 months (eTable 8 in Supplement 2); 18 SAEs were unrelated to the group allocation. The 24 SAEs occurred in 17 participants, with 1 individual experiencing 4 SAEs. Of the 24 SAEs, 9 events were caused by exacerbation of IIH leading to hospitalization. No patients underwent emergency surgery for IIH in the first year. During 12 to 24 months, 1 patient in the weight management arm underwent CSF shunting for deterioration of IIH.

By 24 months, 6 related SAEs were reported in the whole cohort. One related SAE in the weight management arm was a post-LP headache. The 5 related SAEs in the surgery arm included 4 events that were treated conservatively, comprising 1 post-LP headache, 1 delayed discharge immediately after surgery, and 1 hospital admission each for vomiting and epigastric pain, both of which resolved spontaneously. One SAE comprised a hospital admission with vomiting, which was identified through diagnostic laparoscopy to be caused by obstruction at the site of the mesenteric closure. The mesenteric stitch was removed, and the participant experienced no further events. There were no deaths in the 24-month period among participants in either group.

## Discussion

To our knowledge, the IIH:WT is the first randomized clinical trial to evaluate the efficacy of bariatric surgery compared with a community weight management intervention among patients with active IIH. A significant difference was found in the primary outcome of ICP at 12 months. Reduction in ICP among patients with IIH has been associated with disease remission, which enables papilledema resolution and improvement in headache symptoms.^6^ The results of this clinical trial therefore support the use of bariatric surgery as an effective treatment approach among patients with active IIH who have a BMI of 35 or higher, with an enduring effect at 24 months.

Idiopathic intracranial hypertension has been reported to adversely affect patients’ quality of life.^2^ The IIH:WT documented significant improvements in physical component score, energy and fatigue physical functioning, and general health (eTable 7 and eTable 8 in Supplement 2) after bariatric surgery. At 24 months, there were significant differences in outcomes, supporting the use of bariatric surgery for the improvement of physical functioning and general health (eTable 8 in Supplement 2). These improvements could reflect the receipt of bariatric surgery because this surgery is associated with benefits for quality of life as well as with IIH remission.^21^ No improvements in mental component scores were observed in other domains, which is consistent with the findings of a meta-analysis comprising clinical trials that examined bariatric surgery.^22^

Bariatric surgery delivers a wider range of health benefits compared with conservative medical methods for weight loss.^23^ A meta-analysis reported that Roux-en-Y gastric bypass surgery was associated with better outcomes compared with other types of bariatric procedures and weight management programs.^24^ Roux-en-Y gastric bypass surgery has also been associated with a reduced risk of cardiovascular disease compared with routine care.^12^

These cardiovascular improvements and their positive implications for other comorbidities, such as polycystic ovarian syndrome, may be of additional benefit for those with IIH because IIH is associated with a 2-fold increased risk of worse cardiovascular outcomes^4^ and polycystic ovarian syndrome.^1^ Future clinical trials should investigate which type of bariatric surgical procedure is superior for patients with IIH.

The complication rates of bariatric surgery have improved over time, with the mortality rate currently reported to be 0% to 0.64%.^25^ In the IIH:WT, both trial withdrawal and SAE rates were low, with only 1 participant requiring further surgical intervention.

### Limitations

This study has several limitations. The type of bariatric procedure was not predetermined for the surgical arm because the development of the study design was based on pragmatic considerations to reflect routine clinical practice. As a consequence, the number of participants in the trial was too low to confidently recommend 1 surgical procedure over another. This clinical trial was also unable to evaluate patient-centered outcomes because of the relatively low number of participants required to power the study to achieve its primary outcome. Powering the study to achieve meaningful secondary outcome analyses would have required a 5-fold increase in the number of participants. Therefore, we were not able to develop meaningful inferences about the effects of bariatric surgery on the secondary outcomes.

For practical reasons, the physicians performing the LPs were not masked to the results. Although they were masked to the treatment arms to which the participants had been assigned, the difference in weight loss between the 2 arms would have made complete masking a challenge. Applying the results of this clinical trial to a broader population of participants with IIH is limited by the study’s inclusion criteria; thus, the findings do not directly inform treatment among men or women with a BMI lower than 35. These individuals may benefit from bariatric surgery because it has been reported to have favorable metabolic and glycemic implications for those with a BMI between 30 and 35.^23^

## Conclusions

In this randomized clinical trial, bariatric surgery among patients with active IIH had favorable sustained outcomes with regard to reductions in ICP, disease remission, and superior quality of life outcomes at 2 years compared with a community weight management intervention. These results can be used to develop recommendations for health care strategies and to inform health policy decisions regarding bariatric surgery for individuals with active IIH.

## Notes

Supplement 1. Trial Protocol

Supplement 2. **eMethods 1.** Clinical Trial Investigators and Sites, Bariatric Surgery Pathway Team, Patient Identification Centers, and Study Oversight Committees **eMethods 2.** Community Weight Management Intervention, Bariatric Surgery Pathway, and Hierarchical Regression Analysis **eResults.** Relevant Medication Changes Over the Course of the Clinical Trial **eTable 1.** Inclusion and Exclusion Criteria of the IIH:WT **eTable 2.** IIH:WT Schedule of Events **eTable 3.** Secondary Outcome: Headache **eTable 4.** Secondary Outcome: Visual Data (Worst Eye) **eTable 5.** IIH Symptoms, Baseline to 12 Months **eTable 6.** Quality of Life and Hospital Anxiety and Depression Scores **eTable 7.** Quality of Life Subscales as Measured by the SF-36 **eTable 8.** Serious Adverse Events at 12 and 24 Months

Supplement 3. Data Sharing Statement
