# Supplementary material for: Effectiveness of Bariatric Surgery vs Community Weight Management Intervention for the Treatment of Idiopathic Intracranial Hypertension: A Randomized Clinical Trial
Source: JAMA Neurol. 2021 Apr 26;78(6):678–86. doi: 10.1001/jamaneurol.2021.0659 (PMC8077040; doi:10.1001/jamaneurol.2021.0659)
Supplement: Supplement 1. — Trial Protocol [file jamaneurol-e210659-s001.pdf]

A randomised controlled trial of bariatric surgery versus a community weight loss programme for the sustained treatment of Idiopathic Intracranial Hypertension: the IIH:WT Trial

IIH:WT trial

TRIAL PROTOCOL: VERSION 4.1 13th September 2019

Sponsor: University of Birmingham (RG_12-089)

Chief Investigator: Professor Alex Sinclair, University of Birmingham

Coordinating Centre: **Birmingham Clinical Trials Unit (BCTU)**

Funder: National Institute for Health Research (NIHR-CS-011-028)

REC Ref. No.: **14/WM/0011**

ISRCTN40152829

IRAS ID: 142942

32 TRIAL COLLABORATIVE GROUP AND CONTACT DETAILS

**Chief Investigator**	
Professor Alex Sinclair	Professor of Neurology<br>University of Birmingham<br>Email: a.b.sinclair@bham.ac.uk
**Co-Investigators**	
Mr Mike Burdon	Consultant Neuro-ophthalmologist<br>Queen Elizabeth Hospital, Birmingham<br>Email: mike.burdon@uhb.nhs.uk
Professor Carl E Clarke	Professor of Clinical Neurology, Honorary Consultant Neurologist<br>Sandwell and West Birmingham Hospitals NHS Trust<br>Email: carlclarke@nhs.net
Professor Emma Frew	Professor in Health Economics<br>University of Birmingham<br>Email: e.frew@bham.ac.uk
Natalie Ives	Statistics Lead<br>Birmingham Clinical Trials Unit<br>University of Birmingham<br>Email: n.j.ives@bham.ac.uk
Mr Andrew Jacks	Consultant Neuro-ophthalmologist<br>Queen Elizabeth Hospital, Birmingham<br>Email: andrew.jacks2@uhb.nhs.uk
Mr Tim Matthews	Consultant Neuro-ophthalmologist<br>Queen Elizabeth Hospital, Birmingham<br>Email: tim.matthews@uhb.nhs.uk
Mrs Susan Mollan	Consultant Neuro-ophthalmologist<br>Queen Elizabeth Hospital, Birmingham<br>Email: susan.mollan@uhb.nhs.uk
**Collaborators**	
Mr Rishi Singhal	Consultant Bariatric Surgeon<br>Birmingham Heartlands Hospital<br>Email: rishi.singhal@heartofengland.nhs.uk
**Sub-study collaborators**	
Mr Andrew Clouter	Research Associate<br>University of Birmingham<br>cloutera@adf.bham.ac.uk
Dr Peter Nightingale	Statistician<br>University of Birmingham<br>Email: p.g.nightingale@bham.ac.uk
Dr Yusuf Rajabally	Consultant Neurologist<br>Queen Elizabeth Hospital, Birmingham<br>Email: yusuf.rajabally@uhb.nhs.uk

Professor Kim Shapiro	Professor and Chair of Cognitive Neuroscience<br>University of Birmingham<br>Email: k.l.shapiro@bham.ac.uk
Professor Mark Wagshul	Associate Professor<br>Albert Einstein College of Medicine, New York, USA<br>Email: mark.wagshul@einstein.yu.edu
**Clinical Trials Unit (BCTU)**<br>**Trial Management**	
Sarah Tearne	Neuroscience Trials Team Leader<br>Birmingham Clinical Trials Unit<br>University of Birmingham<br>Email: s.clarke.2@bham.ac.uk
Ryan Ottridge	Senior Trial Manager<br>Birmingham Clinical Trials Unit<br>University of Birmingham<br>Email: r.ottridge@bham.ac.uk
Rebecca Woolley	Statistician<br>Birmingham Clinical Trials Unit<br>University of Birmingham<br>Email: r.l.woolley.1@bham.ac.uk
**Trial Steering Committee**	
Professor Alex Sinclair (Chief Investigator)	Professor Neurology<br>University of Birmingham<br>Email: a.b.sinclair@bham.ac.uk
Professor Gordon Plant (Chair)	Consultant Neurologist<br>King's College Hospital<br>Email: gordon.plant@uclh.nhs.uk
Krystal Hemmings	Patient Representative<br>Trustee, IIH UK (Patient charity)<br>Email: krystalhemmings@iih.org.uk
Mr Amir Khan	Medical Director<br>Walsall Manor Hospital<br>Email: amir.khan@walsallhealthcare.nhs.uk
Jill Mollison	Statistician<br>University of Oxford<br>Email: jill.mollison@phc.ox.ac.uk
**Data Monitoring Committee**	
Dr Pearse Keane (Chair)	NIHR Clinical Lecturer<br>Moorfields Eye Hospital and UCL Institute of Ophthalmology, London<br>pearse.keane@moorfields.nhs.uk

Neil Corrigan	Medical Statistician<br>Leeds Institute of Clinical Trials Research, University of Leeds<br>n.corrigan@leads.ac.uk
Mr Kamal Mahawar	Consultant Bariatric Surgeon<br>City Hospitals Sunderland<br>kamal.mahawar@chsft.nhs.uk

Protocol Amendments

The following amendments and/or administrative changes have been made to this protocol since the implementation of the first approved version.

Amendment number	Date of amendment	Protocol version	Type of amendment	Summary of amendment
N/A	20th March 2014	V1.2	Non-substantial	Addition of ISRCTN
1	27th March 2014	V1.3	Substantial	Addition of bypass surgery choice
2	23rd July 2014	V1.4	Substantial	Addition of STOP-Bang and Ravens
3	11th November 2014	V1.5	Substantial	Addition of surgical controls and assessment flexibility
4	30th June 2015	V1.6	Substantial	Addition of post-op visit and change of headache grading
5	20th January 2016	V1.7	Substantial	Reduction of eligibility criteria from 6m to 2m
6	2nd February 2016	V2.0	Substantial	Addition of sites and sleeve gastrectomy surgery choice
7	6th February 2017	V3.0	Substantial	Increase recruitment from 60 to 64
8	14th February 2017	V4.0	Substantial	Changes to ensure adherence to updated BCTU SOPs
9	10th May 2019	V4.0	Substantial	Re-opening fat/skin sample recruitment
10	13th September 2019	V5.0	Substantial	Addition of long term follow up and streamlined subset of controls

COMPLIANCE STATEMENT

This protocol describes the IIH:WT trial only. The protocol should not be used as a guide for the treatment of patients not taking part in the IIH:WT trial.

The trial will be conducted in accordance with the approved protocol, UK Policy Framework for Health and Social Care Research 2017, the Data Protection Act 2018 and the EU General Data Protection Regulation 2018, and the principals of Good Clinical Practice as defined by the European Good Clinical Practice (GCP) Directive. Every care has been taken in the drafting of this protocol, but future amendments may be necessary, which will receive the required approvals prior to implementation.

ROLES

University of Birmingham is the sponsor. Professor Alex Sinclair is the Chief Investigator.

Birmingham Clinical Trials Unit is responsible for obtaining necessary approvals, the Trial Management Group is jointly responsible for overseeing good clinical practice and the Investigators are responsible for obtaining informed consent and care of the participants.

The Trial Management Group consists of the Chief Investigator, Health Economist, Statisticians, and BCTU Trial Management staff.

Neuroscience Trials Office

For general protocol related queries and supply of trial materials:

Birmingham Clinical Trials Unit (BCTU), College of Medical & Dental Sciences, Public Health Building,
University of Birmingham,
Birmingham B15 2TT

Telephone: 0121 415 9125
Fax: 0121 415 9135
Email: neuroscience@trials.bham.ac.uk
Website: www.birmingham.ac.uk/IIHWT

Randomisation / Registration

Telephone: 0800 953 0274 (toll free in the UK, available 9am-5pm Monday – Friday)

Safety Reporting

Fax SAE Forms to: 0121 415 9135 or email to neuroscience@trials.bham.ac.uk

Chief Investigator and Sponsor Signatures

The Chief Investigator and the Sponsor have discussed this protocol. The Investigator agrees to perform the investigations and to abide by this protocol.

The Investigator agrees to conduct the trial in compliance with the approved protocol, the UK Data Protection Act (2018), the Trust Information Governance Policy (or other local equivalent) and the UK Policy Framework for Health and Social Care Research 2017.

For University of Birmingham sponsored trials, the sponsor will confirm approval of the protocol by signing the IRAS form and therefore a signature on the protocol is not required.

Chief investigator
Professor Alex Sinclair,
Professor of Neurology
University of Birmingham

Signature Date

Principal Investigator Signature Page

Principal Investigator:
I have read and agree to the protocol, as detailed in this document. I agree to adhere to the protocol as outlined and agree that any suggested changes to the protocol must be approved by the Research Ethics Committee (REC).

I am aware of my responsibilities as an Investigator under the guidelines of the UK Policy Framework for Health and Social Care Research 2017, Good Clinical Practice (GCP), the Declaration of Helsinki and the trial protocol and I agree to conduct the trial according to these guidelines and to appropriately direct and assist the staff under my control who will be involved in the trial.

Principal investigator
<insert name>

Signature Date

Name of Institution
<insert name>

Each Principal Investigator should sign this page and return a copy to the Neuroscience Trials Office

LIST OF ABBREVIATIONS

**AE**	Adverse Event
**AHI**	Apnea-Hypopnea Index
**BCTU**	Birmingham Clinical Trials Unit
**BHH**	Birmingham Heartlands Hospital
**BMI**	Body Mass Index
**CI**	Chief Investigator
**CRF**	Case Report Form
**CSF**	Cerebrospinal Fluid
**DCF**	Data Clarification Form
**DMC**	Data Monitoring Committee
**GCP**	Good Clinical Practice
**HIT-6**	Headache Impact Test-6
**ICP**	Intracranial Pressure
**IIH**	Idiopathic Intracranial Hypertension
**ISRCTN**	International Standard Randomised Control Trial Number
**LAGB**	Laparoscopic Adjustable Gastric Banding
**LogMAR**	Logarithmic mean angle of resolution
**LP**	Lumbar Puncture
**LSG**	Laparoscopic Sleeve Gastrectomy
**MRI**	Magnetic Resonance Imaging
**MRV**	Magnetic Resonance Venography
**NICE**	National Institute for Health and Care Excellence
**NIHR**	National Institute for Health Service Research
**OSA**	Obstructive Sleep Apnoea
**OCT**	Optical Coherence Tomography
**PBMC**	Peripheral Blood Mononuclear Cells
**PI**	Principal Investigator
**PIC site**	Participant Identification Centre
**PIS**	Participant Information Sheet
**PN**	Peripheral Neuropathy
**QALY**	Quality-Adjusted Life Year
**R&D**	Research and Development
**REC**	Research Ethics Committee
**RYGBP**	Roux-en-Y Gastric Bypass
**SAE**	Serious Adverse Event
**SART**	Sustained Attention to Response Task
**TMF**	Trial Master File
**TMG**	Trial Management Group
**TSC**	Trial Steering Committee
**UENS**	Utah Early Neuropathy Score
**UHB**	University Hospitals Birmingham NHS Foundation Trust
**UoB**	University of Birmingham
**WTP**	Willingness to Pay

CONTENTS

COMPLIANCE STATEMENT....4
ROLES....5
LIST OF ABBREVIATIONS....8
TRIAL SUMMARY....12
TRIAL SCHEMA....14

1. BACKGROUND....16
1.1. Idiopathic Intracranial Hypertension (IIH)....16
1.2. Current therapy for IIH....16
1.3. Weight loss....16
1.4. Bariatric surgery for IIH....17
1.5. The choice of questions to be asked....17
1.5.1 Rationale....17
1.5.2 Risks and benefits....18
1.6. Objective....18
1.7. Exploratory objectives....18

2. TRIAL DESIGN....19
2.1. Design....19
2.2. Primary Aims....19
2.3. Secondary Aims....19
2.4. Setting....19
2.5. Target population....19
2.6. Treatment arms....19
2.7. Primary Outcome Measure....20
2.8. Secondary Outcome Measures....20

3. ELIGIBILITY....20

4. CONSENT AND RANDOMISATION....21
4.1. Informed consent process....21
4.2. Identifying potential participants....21
4.3. Pre-screening....21
4.4. Screening/baseline Visit....22
4.5. Written informed consent....22
4.6. Randomisation....23
4.7. Informing the participant's GP....23
4.8. Ineligible patients....23
4.9. Optional consent to collection of NHS routine clinical datasets....23

5. TREATMENT ALLOCATIONS....24
5.1. Trial intervention: bariatric surgery....24
5.2. Trial intervention: dietary weight loss programme....24
5.3. Compliance monitoring....24
5.4. Concomitant therapy....25
5.4.125
5.5. Excluded medications or interactions....25
5.6. Withdrawal of treatment or protocol violation....25

6. FOLLOW-UP AND ASSESSMENTS....25
6.1. Format of assessment visits....25

6.2. Screening/baseline visit ... 25
6.3. Follow up visits ... 28
6.4. Post-surgery visits ... 28
6.5. Withdrawal ... 28
6.6. Timing of assessments ... 28
6.7. Participant completed questionnaires ... 28
6.8. Assessment methods ... 29
6.9. Blood and CSF samples ... 31
6.10. Processing and storage of samples ... 31

7. ADVERSE EVENT REPORTING ... 32
7.1. Assessment of Safety ... 32
7.2. Serious Adverse Events ... 32
7.3. SAE reporting procedures ... 33
7.4. Assessment of relatedness ... 33
7.5. Assessment of expectedness ... 33
7.6. Expected Adverse Events ... 34
7.7. Related and Unexpected SAEs ... 34
7.8. Annual Progress Reports ... 34
7.9. Reporting urgent safety measures to the REC ... 34
7.10. Data Monitoring Committee ... 34
7.11. Notification of Serious Breaches of GCP and/or the protocol ... 35

8. DATA MANAGEMENT AND VALIDATION ... 35
8.1. Source Data ... 35
8.2. Confidentiality of personal data ... 35
8.3. Long-term storage of data ... 36
8.4. Data management ... 36
8.5. Definition of the End of Trial ... 36

9. STATISTICAL CONSIDERATIONS ... 37
9.1. Sample size ... 37
9.2. Projected accrual and attrition rates ... 37
9.3. Statistical Analysis ... 37
9.4. Missing Data and Sensitivity Analyses ... 38
9.5. Subgroup Analyses ... 38
9.6. Interim Analyses ... 38
9.7. Final Analyses ... 38

10. HEALTH ECONOMIC OUTCOMES ... 38
10.1. Health economic outcomes ... 38
10.2. Overall objective ... 39
10.3. Methods ... 39
10.4. Economic evaluation ... 41

11. EXPLORATORY SUB-STUDIES ... 41
11.1. Exploratory Aims ... 41
11.2. Exploratory Outcome Measures ... 42
11.3. Changes to participant pathway to incorporate sub-studies ... 42
11.4. Sleep apnoea observational cohort sub-study ... 44
11.5. Metabolic syndrome sub-study ... 44
11.6. Magnetic resonance imaging in IIH sub-study ... 46
11.7. Cognitive function sub-study ... 47

11.8. Exploratory samples ... 48
11.9. Processing and storage of additional samples ... 49

12. EXPLORATORY SUB-STUDIES: ADDITIONAL RECRUITMENT GROUPS ... 50
12.1. Matched obese control group sub-study ... 50
12.1.1 Eligibility for matched obese control group sub-study ... 51
12.2. MRI Test Run sub-study ... 52
12.2.1 Eligibility for MRI Test Run sub-study ... 52
12.3. Control Fat and Skin Sample sub-study ... 53
12.3.1 Eligibility for Control Fat and Skin Sample sub-study ... 53

13. DATA ACCESS AND QUALITY ASSURANCE ... 53
13.1. Monitoring and Audit ... 53
13.2. Site Set-up and Initiation ... 53
13.3. Central Monitoring ... 54
13.4. Notification of Serious Breaches ... 54
13.5. Independent Trial Steering Committee ... 54
13.6. Data Monitoring Committee: determining when clear answers have emerged ... 54

14. ORGANISATION AND RESPONSIBILITIES ... 55
14.1. Principal Investigator at each centre ... 55
14.2. Nursing Co-ordinator at each centre ... 55
14.3. The Neuroscience Trials Office ... 55
14.4. Research Governance ... 55
14.5. Ethical and Trust Management ... 56
14.6. Funding and Cost implications ... 56
14.7. Indemnity ... 56
14.8. Publication ... 56

15. PROJECT TIMETABLE AND MILESTONES ... 57

16. APPENDIX A: FRISÉN GRADING ... 58

17. REFERENCES ... 59

TRIAL SUMMARY

EXECUTIVE SUMMARY	
**Title**	A randomised controlled trial of bariatric surgery versus a community weight loss programme for the sustained treatment of Idiopathic Intracranial Hypertension: the IIH:WT Trial.
**Acronym**	IIH:WT
**Trial design and methods**	Randomised controlled parallel arm trial with patients randomised 1:1 to bariatric surgery or dietary weight loss programme.
**Total number of participants planned**	64 (plus at least 20 obese controls, 5 MRI test run volunteers, and 40 fat and skin sample controls).
**Trial duration per participant**	60 months with assessments at baseline, then at 3, 6, 12, 24 and 60 months.
**Accrual period**	45 months (randomised participants, 26 further months for all controls)
**Estimated total trial duration**	6 months for set up, 45 months for recruitment, 60 months for follow up, 6 months for final analysis and write up of results: 117 months.
**Primary study objectives**	The trial will evaluate the effectiveness of two methods of weight loss in the treatment of IIH: bariatric surgery vs. dietetic intervention. The primary outcome will be change in intracranial pressure between baseline and 12 months.
**Main inclusion criteria**	• Female IIH patients aged between 18 and 55 years, diagnosed according to the modified Dandy criteria who have active disease (papilloedema in at least one eye, significantly raised ICP > 25cmCSF) of over 2 months duration and normal brain imaging (magnetic resonance imaging and venography as noted at diagnosis).<br>• Body mass index (BMI) >35kg/m^2.<br>• Tried other appropriate non-surgical treatments to lose weight but have not been able to achieve or maintain adequate, clinically beneficial weight loss for at least 6 months.<br>• Able to give informed consent.
**Main exclusion criteria**	• Age less than 18 or older than 55 years.<br>• Pregnant.<br>• Significant co-morbidity, Cushing's syndrome, Addison's disease or the use of oral or injected steroid therapy.<br>• Undergone optic nerve sheath fenestration.<br>• Definite indication for or contraindication against surgery or dieting.<br>• Have a specific medical or psychiatric contraindication for surgery, including drug misuse, eating disorder or major depression (suicidal ideation, drug overdose or psychological admission in last 12 months).<br>• Previous bariatric surgery.<br>• Inability to give informed consent e.g. due to cognitive impairment.

LAY SUMMARY
Idiopathic intracranial hypertension (IIH) is a condition with an unknown cause or causes. The condition is associated with raised pressure in the brain and can cause disabling daily headaches and visual loss, which can be permanent. The raised brain pressure squashes the nerves supplying the eye (also known as papilloedema) and this can affect vision.<br><br>Over 90% of patients with IIH are overweight and weight loss is the most effective treatment. Other

treatments for IIH have very little current evidence to support their use. This trial aims to compare two methods of weight loss, bariatric surgery and the most effective dietary weight loss programme commonly available, Weight Watchers, to see which offers the most effective long-term treatment for IIH. Bariatric surgery is recommended by the NICE clinical guidelines for patients with a Body Mass Index (BMI) of over 40 kg/m^2, or over 35 $kg/m^2$with a co-morbidity. Women suffering from IIH have a BMI on average around 38 kg/m^2 and IIH is not recognised as a co-morbidity for bariatric surgery.

This trial will recruit 64 women from UK NHS Trusts. They will be randomised and 32 participants will be allocated to the dietary weight loss arm, and enrolled in their local Weight Watchers group. 32 participants will be allocated to the bariatric surgery arm, and referred to their local bariatric surgery pathway to receive gastric banding, gastric bypass, or sleeve gastrectomy according to patient and surgeon preference. Both groups of participants will be allocated to a treatment arm which is proven to bring about weight loss.

A control group of at least 20 women with similar characteristics, but who do not have IIH will provide a pre-intervention comparison. A second group of control participants without IIH but undergoing bariatric surgery will donate fat and skin samples to optimise the laboratory experiments that will be carried out on samples taken from the bariatric surgery arm participants. At least 5 volunteers will also be recruited to undergo 2 MRI test scans to validate the MRI sequences being used in the trial. These groups will not participate any further in the trial.

Participants with IIH entered into the randomised trial will then be followed up for five years, with the most important measurement being their brain pressure after one year of being in the trial. The main risk is to patients in the bariatric surgery arm: weight loss surgery, although safe, is a major operation, and careful follow up is required. Laparoscopic gastric banding has a mortality rate of less than 0.1%, and both laparoscopic gastric bypass and laparoscopic sleeve gastrectomy have a mortality rate of less than 0.2%.

Participants will need to provide informed consent; those who are unable to do so will not be enrolled in the trial.

Participants with IIH and the 20 obese controls will also be asked to give samples of urine, blood, and cerebrospinal fluid. Some participants, including the obese controls, will also be asked to participate in sub-studies to look at the relationship between IIH and other illnesses connected with obesity, from which they may suffer. These samples and sub-studies may provide valuable insight into the causes of IIH and future treatment options.

TRIAL SCHEMA

Figure 1 – For trial participants with IIH

NB: Typical surgery pathway as followed at Birmingham Heartlands Hospital (BHH)

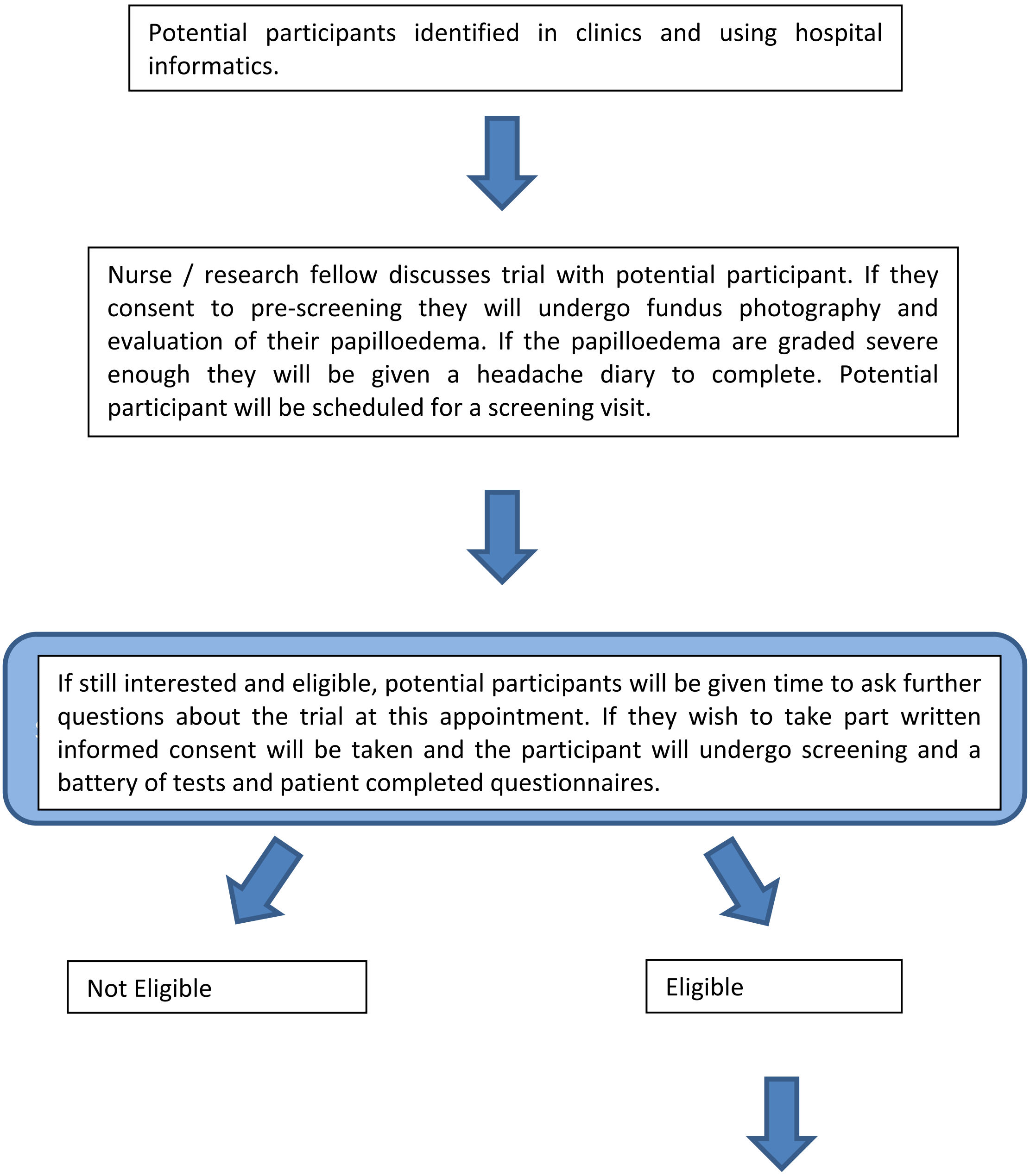

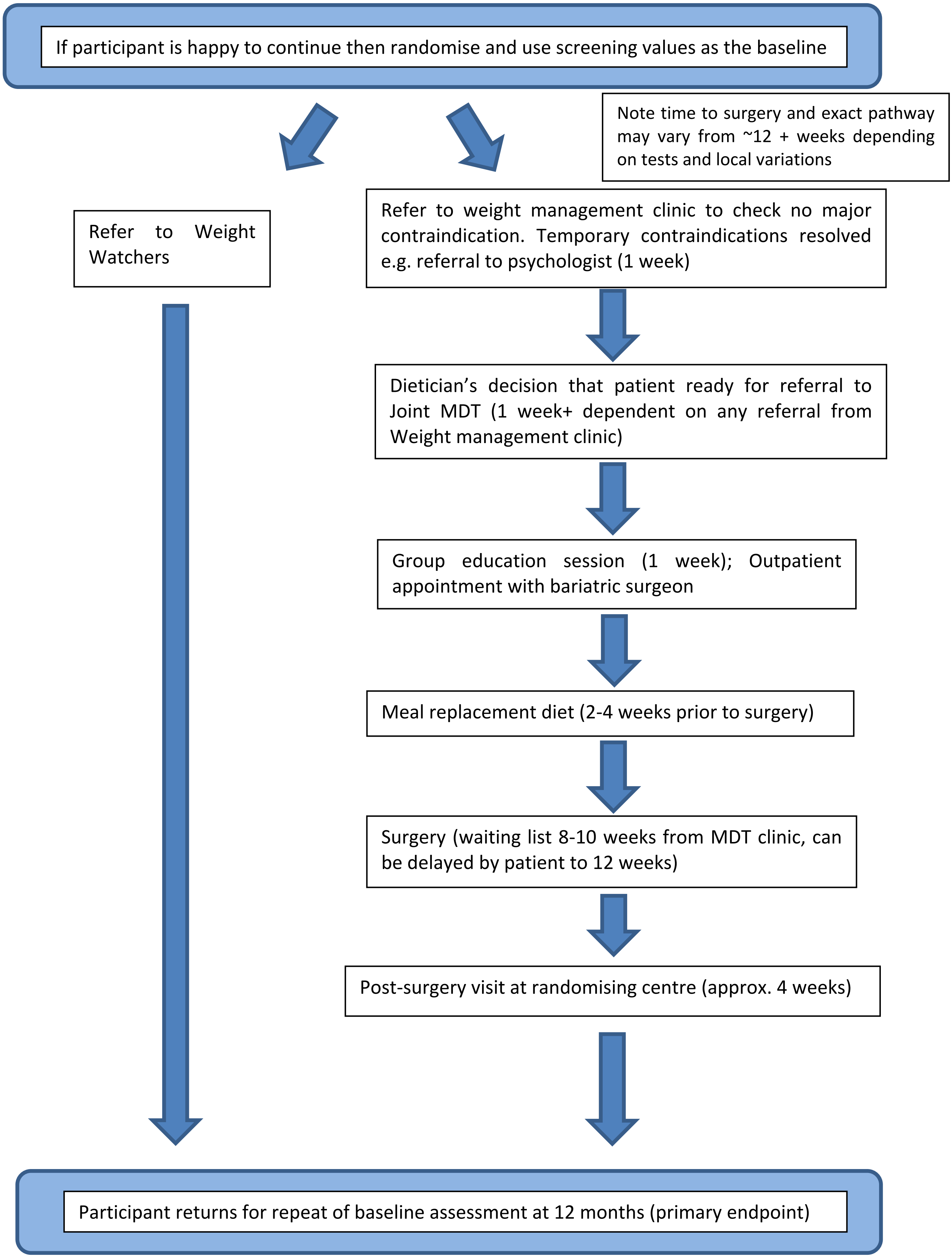
If participant is happy to continue then randomise and use screening values as the baseline
Note time to surgery and exact pathway may vary from ~12 + weeks depending on tests and local variations
Refer to Weight Watchers
Refer to weight management clinic to check no major contraindication. Temporary contraindications resolved e.g. referral to psychologist (1 week)
Dietician's decision that patient ready for referral to Joint MDT (1 week+ dependent on any referral from Weight management clinic)
Group education session (1 week); Outpatient appointment with bariatric surgeon
Meal replacement diet (2-4 weeks prior to surgery)
Surgery (waiting list 8-10 weeks from MDT clinic, can be delayed by patient to 12 weeks)
Post-surgery visit at randomising centre (approx. 4 weeks)
Participant returns for repeat of baseline assessment at 12 months (primary endpoint)

1. BACKGROUND

1.1. Idiopathic Intracranial Hypertension (IIH)

IIH, also known as benign intracranial hypertension or pseudotumour cerebri, is a condition of unknown aetiology characterised by elevated intracranial pressure (ICP) and papilloedema. IIH is a condition found primarily in obese women (90%), causing disabling daily headaches and visual loss, which is severe and permanent in up to 25% of cases [1]. Effective treatments are lacking and range from unproven medical therapy to surgical procedures which offer symptomatic relief and prevent blindness. Amongst the obese female population, the incidence of IIH is 20 per 100,000. Worldwide, the number of obese individuals has doubled since 1980 with 22.7% of the UK population being characterised as obese (body mass index (BMI) >30kg/m^2) [2] and in line with the global epidemic of obesity, the prevalence of IIH is expected to rise and consequently contribute significant morbidity to the young female obese population.

1.2. Current therapy for IIH

The 2005 Cochrane review concluded that there was insufficient evidence to determine which treatments were potentially beneficial and which were harmful in IIH [3]; hence there are no specific guidelines regarding the treatment of IIH.

Medical therapy, typically carbonic anhydrase inhibitors, has been used with the aim of lowering ICP, although evidence of efficacy is lacking. Our pilot study of 50 patients comparing the carbonic anhydrase inhibitor acetazolamide to control showed improvement in both arms, however this trial was not powered to determine a difference between the two treatment arms [4]. Topiramate, a carbonic anhydrase inhibitor with weight loss properties, has been evaluated in IIH and was found to induce weight loss, but this trial is difficult to interpret since no therapeutic benefit on IIH was noted above the control cohort treated with acetazolamide (visual field grades improved from baseline in both groups, but there was no statistically significant difference between groups) [5].

In cases of deteriorating vision, surgical techniques such as cerebrospinal fluid (CSF) diversion (shunting) or optic nerve sheath fenestration can be used to prevent blindness. The incidence of CSF shunting procedures to lower ICP is rising rapidly in the USA in line with the growing obesity figures [6]. Shunting itself is a far from satisfactory treatment of IIH. Our audit at University Hospitals Birmingham NHS Foundation Trust recorded 127 shunt insertions for patients with IIH between 1998 and 2008 resulting in short-term significant visual improvement [7]. However, 79% of patients continued to suffer with headaches at 2 years, with 28% having iatrogenic low pressure headaches. Shunt revision occurred in 51%, with 30% requiring multiple revisions. Our data and that of others confirm the significant morbidity and low mortality from CSF shunting [8]. Patients waiting for a shunt and suffering disabling headaches with very high pressures can also be offered repeated lumbar punctures (LP) to lower ICP and thereby offer symptomatic relief.

1.3. Weight loss

Weight loss has been suggested as a treatment strategy in IIH, but the only prospective evidence of efficacy came from an uncontrolled study of 9 patients on a low calorie rice diet, who were subjectively observed to improve [9]. We have published a seminal prospective study of 25 participants which demonstrated that use of a very low calorie diet leading to weight loss and significantly reduced BMI (loss of 15.3% ± 7.0% of body weight) significantly lowered ICP (-8.0 ± 4.2 cmCSF, $p<0.001$) and significantly improved papilloedema, vision and headache symptoms [10].

There are no published systematic reviews or meta-analyses of weight modification or bariatric surgery in IIH, although an ever increasing number of case series and case reports (62 documented cases reviewed as of 2011) describe the beneficial effects of bariatric surgery in IIH [11].

1.4. Bariatric surgery for IIH

Bariatric surgery has many advantages as a potential treatment for IIH:

1) Weight loss is greater than that observed with other weight reducing approaches [12]. Patients typically lose 30% of body weight by one year [13];
2) Weight loss is sustained [12];
3) Other obesity related co-morbidities such as diabetes are improved [14-16];
4) Life expectancy is significantly increased, particularly in young patients as they have the lowest surgical risk and longest life expectancy to benefit from the resulting weight loss [17];
5) There are a number of bariatric surgical procedures which can either reduce the gastric capacity (e.g. gastric banding [LAGB]) or reduce intestinal absorption as well as reducing gastric capacity (e.g. Roux-en-Y gastric bypass [RYGBP]) enabling the surgery to be tailored to the individual patient's needs;
6) Bariatric surgery is a cost-effective intervention compared to non-surgical interventions to manage obesity, even in those with mild obesity (BMI >30 kg/m^2) [18];
7) Bariatric surgery appears to have low associated morbidity and mortality: Hutter et al show 30 day mortality rates of 0.05% for LAGB, 0.11% for the newer laparoscopic sleeve gastrectomy (LSG) procedure, and 0.14% for RYGB [19]. RYGBP and LSG carry the highest risks, but patients undergoing these procedures typically have the highest preoperative morbidity and BMI [19, 20].

The National Institute for Health and Care Excellence (NICE) recommends bariatric surgery for the treatment of morbid obesity (BMI at least 40 kg/m^2) or in people with a BMI of over 35 kg/m^2 in conjunction with other significant disease that may be improved if they lose weight [21].

1.5. The choice of questions to be asked

Weight loss, achieved through intensive dieting, is an effective therapeutic strategy in IIH. However long-term maintenance of weight loss is notoriously poor, which leads to the recurrence of symptoms: patients in the IIH weight loss study were noted to regain weight and consequently their symptoms and signs of IIH returned, a documented phenomenon in the condition [22]. Despite the relapse in IIH following weight gain, our study provides evidence of the efficacy of therapeutic weight loss. Consequently, sustainable approaches to weight loss in IIH are likely to offer patients an effective, potentially curative treatment.

1.5.1 Rationale

Long-term maintenance of weight loss is difficult irrespective of the dietary regime followed [23]. Obesity pharmacological therapies including orlistat and high dose liraglutide reduce weight by an average of 2.89kg and 7.6kg respectively [24, 25]. These data suggest that these drugs are unlikely to achieve sufficient weight loss to significantly modify IIH.

Bariatric surgery has been shown to be a sustainable approach to weight loss [26, 27], and so may offer long-term treatment of IIH. However, very little research and no randomised controlled trials have addressed this question.

Bariatric surgery is an invasive approach to weight reduction and a significant move away from current treatment for IIH. To impact current clinical practice, we feel that bariatric surgery would need to be compared to the best alternative weight loss regime (rather than just current practice). The comparator arm of the study will therefore be a dietary weight loss programme using the internationally recognised Weight Watchers diet.

Weight Watchers is a commercial dietary weight loss programme. This well recognised brand has a large geographical spread and over two million members in the UK. The programme contains both dietary and lifestyle modification advice, and each meeting is conducted according to the usual Weight Watchers guidelines led by a group leader trained by Weight Watchers. Weight Watchers represents the most

effective widely available dietary regime, achieving superior weight loss, attendance and cost effectiveness compared to other commercially available or primary care led weight reduction programmes [28].

Participants in the IIH:WT trial will therefore be randomised between referral to their local bariatric surgery pathway or a dietary weight loss programme, which will be their local Weight Watchers group for 12 months.

1.5.2 Risks and benefits

Participants randomised to the bariatric surgery arm will be referred to the bariatric surgery pathway and, if judged suitable according to the bariatric surgery clinic's screening processes, will undergo LAGB, RYGBP, or LSG. The decision of which surgery to undergo will be made between the surgeon and the participant based upon the participant's health circumstances and preference.

As shown above in 1.4, bariatric surgery is a safe procedure. RYGBP and LSG have a higher mortality rate than LAGB, but are often performed in a higher risk population. Bariatric surgery is particularly safe in our patient population who will typically be young and not morbidly obese (with a mean BMI of 38 g/m^2 [10]), and typically without other co-morbidities of obesity – so their surgical risk is much lower than a patient with morbid obesity (BMI 40-60 kg/m^2) and obesity co-morbidities such as heart disease.

Hutter et al. give a mean reduction in BMI of 7.05 m/kg^2 for LAGB, 11.87 for LSG and 15.34 for RYGBP [19]. These results are more than adequate for achieving the 15.3% ± 7.0% reduction in body weight shown to significantly reduce ICP (-8.0 ± 4.2 cmCSF, $p<0.001$), papilloedema and symptoms in patients with IIH [10].

Participants randomised to the dietary weight loss programme arm of Weight Watchers will benefit from a programme which has the highest success rate of commercially available dietary weight loss programmes [28] and will gain an understanding of nutrition and portion sizes in an environment that offers the support and motivation necessary to lose weight. There are no known risks to taking part in the Weight Watchers programme [28-31].

The benefits of both trial arms in our patient population will also be increased as they are relatively young and will have more years to enjoy the advantages of weight loss in terms of overall improved health and reduction in co-morbidities.

The main risk to participants in this trial is in the surgical procedure as described above. The assessment and management of risk is detailed in the separate IIH:WT Risk Assessment document. An ongoing evaluation of risk will continue throughout the trial.

1.6. Objective

We wish to assess if weight loss through bariatric surgery and / or dietary weight loss programme is an effective sustainable treatment for IIH, with sustained reduction of ICP, visual symptoms and headaches.

1.7. Exploratory objectives

As part of the trial, there are also a number of exploratory objectives which will be assessed through various optional sub-studies. These will not be carried out at all sites:

- Sleep apnoea observational cohort sub-study
- Metabolic syndrome sub-study
- Magnetic resonance imaging in IIH sub-study
- Cognitive function sub-study
- Matched obese control group
- Obese sample control group
- MRI test run sub-study

See sections 11 and 12 for further information on the sub-studies.

2. TRIAL DESIGN

2.1. Design

We will conduct a randomised controlled parallel arm trial where participants will be randomised in a 1:1 ratio to an NHS bariatric surgery pathway or to a community based Weight Watchers dietary weight loss programme. Sixty-four participants (32 to each arm) will be randomised.

The trial will necessarily be open label due to the nature of the intervention though assessors of visual outcomes will be masked to randomised treatment allocation. It will not be practical to blind the nurse or clinician undertaking the medical and visual function assessments. The primary outcome, ICP, is an objective measure.

2.2. Primary Aims

The trial will evaluate the effectiveness of two methods of weight loss for the treatment of IIH: bariatric surgery versus a dietary weight loss programme. It will:

- Evaluate if weight loss achieved through bariatric surgery reduces ICP and consequently treats patients with IIH.
- Evaluate if bariatric surgery is more effective than a dietary weight loss programme in reducing ICP and consequently treating patients with IIH.
- Evaluate the long-term effectiveness of bariatric surgery versus a dietary weight loss programme in reducing ICP and consequently treating patients with IIH.

2.3. Secondary Aims

The trial will evaluate the clinical effectiveness, cost-effectiveness and participant-centred clinical outcome measures (e.g. quality of life) of bariatric surgery versus a dietary weight loss programme.

2.4. Setting

Suitable patients will be identified at Neurology and Neuro-ophthalmology clinics in UK NHS Trusts as well as at Participant Identification Centres (PIC sites) as described in 4.2 below. Participants randomised to the bariatric surgery arm will be referred to the local bariatric surgery pathway; participants randomised to the dietary weight loss arm will be enrolled in their local Weight Watchers group.

2.5. Target population

Women with BMI>35kg/m^2, with active IIH (papilloedema [Frisén grade ≥ 1 in at least one eye] and ICP >25 cmCSF) of over 2 months' duration who have tried other appropriate non-surgical treatments to lose weight, but have not been able to maintain weight loss.

2.6. Treatment arms

Intervention arm

- Participants randomised to the bariatric surgery arm of the trial will be referred to the local NHS bariatric surgery pathway.

Active control arm

- Participants randomised to the dietary weight loss arm will be given vouchers that exempt them from paying for consecutive and specified weeks of their local Weight Watchers. Attendance at the groups will be monitored through participant self-reporting.

2.7. Primary Outcome Measure

- ICP (as measured in cmCSF by LP) at 12 months

2.8. Secondary Outcome Measures

- ICP at 24 and 60 months
- Reported IIH symptoms (presence or absence of tinnitus, visual loss, diplopia, visual obscurations and headache) at 12 months (and at 24 and 60 months)
- Visual function in both eyes (measured by LogMAR chart to assess visual acuity, automated perimetry (Humphrey 24-2 central threshold) to measure the visual field mean deviation, a MARS chart to evaluate contrast sensitivity, and Ishihara charts to measure colour vision) at 12 months (and at 24 and 60 months)
- Papilloedema in both eyes at 12 months (measured by masked assessment of fundus photography and by Optical Coherence Tomography scans (OCT)) (and at 24 and 60 months)
- Headache associated disability using the headache impact test-6 score (HIT 6) and headache diary at 12 months (and at 24 and 60 months)
- Anthropometric measures (e.g. waist, hip, fat mass, blood pressure) at 12 months (and at 24 and 60 months)
- Quality of life (participant reported using the EQ-5D-5L, ICECAP-A questionnaire , SF-36 Version 1 questionnaire, Hospital Anxiety and Depression scale (HAD score) and Allodynia Symptom Checklist-12) at 12 months (and at 24 and 60 months)
- Difference in number of referrals to CSF shunting procedures and optic nerve sheath fenestration between treatment arms at 12 months (and at 24 and 60 months)
- Health economics including cost-effectiveness at 12, 24 and 60 months.

3. ELIGIBILITY

Inclusion criteria

1. Female IIH patients aged between 18 and 55 years, diagnosed according to the updated modified Friedman Jacobsen criteria [32] who have active disease (papilloedema [Frisén grade ≥ 1 in at least one eye], significantly raised ICP >25cmCSF) of over 2 months'[*] duration and no evidence of venous sinus thrombosis (magnetic resonance or CT imaging and venography as noted at diagnosis).
2. BMI >35kg/m^2.
3. Tried other appropriate non-surgical treatments to lose weight but have not been able to achieve or maintain adequate, clinically beneficial weight loss for at least 6 months.
4. Able to give informed consent.

Exclusion criteria

1. Age less than 18 or older than 55 years.
2. Pregnant[†].
3. Significant co-morbidity, Cushing's syndrome, Addison's disease or the use of oral or injected steroid therapy.
4. Undergone optic nerve sheath fenestration.
5. Definite indication for or contraindication against surgery or dieting.

[*] A month is defined as 4 weeks.

[†] It is recommended by the bariatric surgery team overseeing the bariatric surgery pathway at BHH that patients do not become pregnant within a year of surgery.

6. Have a specific medical or psychiatric contraindication for surgery, including drug misuse, eating disorder or major depression (suicidal ideation, drug overdose or psychological admission in last 12 months).
7. Previous bariatric surgery.
8. Inability to give informed consent e.g. due to cognitive impairment.

4. CONSENT AND RANDOMISATION

4.1. Informed consent process

The conduct of the trial will be in accordance with the principles of Good Clinical Practice (GCP) as captured in the UK Research Governance Framework (2005 2nd Edition; as amended). The participant's written informed consent to participate in the trial must be obtained before any procedures relating to the trial (including screening) are undertaken and after a full explanation has been given of the trial, the treatment options and the manner of treatment allocation.

Participant information sheets (PIS) and consent forms will be provided so that potential participants can find out more about the trial before deciding whether or not to participate.

4.2. Identifying potential participants

Research staff will identify potential participants in clinic or using informatics. Clinic lists will also be screened before clinics for basic eligibility criteria. These patients will then be approached during their clinic appointment to establish any interest in taking part in the IIH:WT trial. In some cases, potential participants may be posted an appropriately approved invitation letter and PIS (including summary sheet), and will then be followed up by telephone by the research team.

Additionally, potential participants will be identified and referred to trial sites from PIC sites. In these cases the patient details will be sent to the research team at the trial site who will then contact the potential participant. Participants will not be consented at PIC sites.

A hospital poster will be used in appropriate clinics e.g. Neuro-ophthalmology and Neurology. Hospital newsletters and social media may also be used for advertising purposes. Finally, the trial will be advertised on websites such as the IIH UK charity website with a printable consent form allowing the trial site research teams to contact potential participants' doctors for a referral and transfer of patient notes and details.

4.3. Pre-screening

Eligibility should be assessed and documented by a clinician or research nurse and then the process of obtaining written informed consent for pre-screening may be delegated as appropriate (to a suitably trained member of the local research team). This must be clearly documented on the IIH:WT Delegation and Signature Log in the site file.

Potential participants will be offered details of the trial and provided with a short written PIS explaining the pre-screening process (i.e. the current Research Ethics Committee [REC] approved version which should be on appropriately headed paper).

If they are interested, they will be asked to consent to a pre-screening process that will involve having their papilloedema graded. The treating neuro-ophthalmologist will grade the papilloedema clinically using Frisén grading (Appendix A). The papilloedema will be further recorded using fundus photography, which will be carried out at the pre-screening stage where practical (or at baseline if not practical at pre-screening) and then at the 12, 24 and 60 month visits (see 6.1.1 below). If the papilloedema according to the Frisén grading is ≥ 1 in at least one eye the potential participant will be eligible, and they will be given a provisional clinic appointment (baseline/screening visit) in at least a week's time (at least 7 days and no more than 30 days).

They will then be asked to complete a week long headache diary (which will be used as baseline information if they subsequently consent to join the trial) – this should be completed in the week before their clinic appointment.

It is felt that asking the potential participants to complete this headache data before consenting to join the full trial is appropriate as it spares the participant from further hospital visits and will be explained in the pre-screening PIS and included in the pre-screening consent.

It will be explained that there will be a clinic appointment (baseline/screening visit) which will include further screening and tests for taking part in the trial, and that if they are eligible and still want to take part they will be asked to provide informed consent to be entered into the full trial and then randomised to one of the treatment arms. It will also be explained that potential participants should not eat after midnight as fasting blood samples will be required.

Potential participants will have time between this pre-screening visit and the following screening/baseline visit (at least a week) to consider the trial and decide whether or not they wish to take part, and to discuss the trial with their family and friends if they would like to do so. If the potential participant has any questions or queries about the trial during this time they will have the opportunity to discuss the trial with the research staff, whose contact details will be provided on the PIS. It will be explained that if the potential participant takes part in the pre-screening tests but later decides not to take part in the full trial this will not affect their continuing medical care.

4.4. Screening/baseline Visit

The screening/baseline visit (and subsequent 12, 24 and 60 month visits) will be held on a single day or split across more than one day dependent on participant preference and hospital logistics. At this clinic appointment, potential participants will be given plenty of time to discuss the trial further and to have any questions that they may have about the trial answered. The complex nature of the trial, the possibility of undergoing a surgical procedure, and the need to attend hospital for follow up appointments on 5 occasions after the baseline visit will be carefully explained. The Investigator or designee will explain that trial entry is entirely voluntary. It will also be explained that the participant can withdraw at any time during the trial, without having to give a reason and that their decision will not affect the standard of care they receive.

At the end of the screening/baseline visit and before randomisation, confirmation of participant eligibility will be made by a medically qualified doctor.

4.5. Written informed consent

If the potential participant is still willing to participate in the trial then the informed consent process will be conducted by the Investigator or a delegated clinician for entry into the full trial. This will be obtained before any further procedures or collection of data are undertaken once the potential participant is happy that all their questions have been addressed. It will include consent for all the testing that will be completed for screening and during the trial. The PIS will outline that if any of the test results on the screening day do not fulfil the inclusion and exclusion criteria, then individuals will be withdrawn from further screening investigations and from progressing into the trial. However, data and samples from the screening visit will be kept and may be used in sub-studies. Consent will also be taken to inform their GP by letter of their participation in the trial. If written informed consent is given, then the baseline/screening visit will follow the process for assessments as outlined in section 6.2 below.

If the potential participant is eligible after completing screening/baseline testing, then the participant will be randomised and the recorded data (as well as the headache diary completed in pre-screening) used as baseline values.

Any visual assessments which should be carried out at baseline will not be repeated at this visit if, as a routine part of clinical care, they have been carried out in the last 30 days. These previous test results will be used to spare the participant the trouble of undergoing these lengthy tests (45 minutes for the Humphrey Visual Field test) again; this will be acceptable as the visual field, for example, does not vary rapidly with time.

At each visit the participant's willingness to continue in the trial will be ascertained and documented in the medical notes. Throughout the trial the participant will have the opportunity to ask questions about the trial. Any new information that may be relevant to the participant's continued participation will be provided. Where new information becomes available which may affect the participants' decision to continue, participants will be given time to consider and if happy to continue will be re-consented. Re-consent will be documented in the medical notes. The participant's right to withdraw from the trial will remain.

4.6. Randomisation

Randomisation notepads will be provided to researchers and will be used to collate the necessary information prior to randomisation. Participants are entered and randomised into the trial by a telephone call (0800 953 0274) to the toll-free randomisation service at the University of Birmingham Clinical Trials Unit (BCTU). This secure central randomisation service is available Monday-Friday, 09:00-17:00 UK time, except for bank holidays and University of Birmingham closed days, and will ensure concealment of treatment allocation. The person randomising will need to provide answers to all of the questions on the randomisation notepad before a treatment allocation is given. Participants will be randomised in a 1:1 ratio between the two arms of the trial: dietary weight loss programme or bariatric surgery.

Randomisation will be provided by a computer generated allocation list at the BCTU. The randomisation will be stratified according to whether or not the participant is taking acetazolamide at entry.

After randomisation a confirmation of treatment allocation and trial number will be sent by BCTU to the research team.

Investigators will keep their own study file log which links patients with their trial number in the IIH:WT Participant Recruitment and Identification Log. The Investigator must maintain this document, which is not for submission to the Trials Office. The Investigator will also keep and maintain the IIH:WT Screening Log which will be kept in the Investigator Site File, and should be available to be sent to the Trials Office upon request. The IIH:WT Participant Recruitment and Identification Log and IIH:WT Participant Screening Log should be held in strict confidence.

4.7. Informing the participant's GP

The participant's GP will be notified, with the participant's consent; a specimen "Letter to GP" is supplied.

4.8. Ineligible patients

Reasons for non-participation will be recorded if the information is volunteered at any stage of the pre-screening, screening, or informed consent process.

4.9. Optional consent to collection of NHS routine clinical datasets

This trial will include optional consent to allow linkage to patient data available in NHS routine clinical datasets, including primary care data (e.g. Clinical Practice Research Datalink, The Health Improvement Network, QResearch), secondary care data (Hospital Episode Statistics) and mortality data from the Office of National Statistics through NHS Digital and other central UK NHS bodies. The participant will consent to the trial team sending their name, address, date of birth and NHS number to the relevant national registry and then for the national registry to link this to their data and send the information back to the trial team. The consent will also allow access to other new central UK NHS databases that will appear in the future.

This will allow us to extend the follow-up of patients in the trial and collect long-term outcome and health resource usage data without needing further contact with the trial participants.

This is being introduced after the recruitment of all main trial participants. Rather than increase the burden on participants by making a hospital visit for this consent obligatory, sites will have the option to allow the participant to complete this additional consent by post. In this case the PIS addendum will be posted by the site with a prepaid return envelope and the participant contacted by telephone to ensure that they have the same opportunity to discuss taking part that they would have during a hospital visit.

5. TREATMENT ALLOCATIONS

5.1. Trial intervention: bariatric surgery

Participants randomised to the bariatric surgery arm of the trial will be referred to an NHS surgical pathway. As an illustration of a typical pathway, the pathway followed at BHH is given below:

Initially they will be seen in the weight management clinic for medical and psychological assessment for bariatric surgery. This assessment period will last as long as the weight management team find appropriate. Once the weight management team are satisfied that the participant is suitable, they will be discussed in the joint multidisciplinary team meeting prior to attending a group session for education regarding bariatric surgery. The participant will then have an outpatient appointment with the Consultant Bariatric Surgeon and be given a date for surgery. They will be given up to 12 weeks for further consideration of the procedure if they require it. Immediately prior to the operation, participants will undergo a 2-4 week conditioning meal replacement diet (to shrink the liver, thereby increasing the safety of the laparoscopic procedure). This meal replacement diet will not be provided as part of the trial: it is a normal part of the surgical pathway, replacing participants' normal food during the diet, and a particular choice is difficult to enforce or supply due to patient preference and dietary requirements. Participants will choose and instigate this diet after consultation with the bariatric team. Post-surgery, participants will undergo (8, in the case of LAGB) follow up visits over 2 years as part of the standard surgical follow up. They will then remain indefinitely in touch with the bariatric unit should they need future advice or follow-up. It is envisioned that the standard patient pathway will take approximately 4 months from randomisation to surgery, but in exceptional cases at the decision of the bariatric team this may be longer.

In the rare cases where the laparoscopic procedure has to be converted to open surgery the participant will still be included in the trial and followed up normally.

In all cases, the choice of operation will be decided between participant and surgeon, and standard NHS follow up as required will be included in the treatment.

5.2. Trial intervention: dietary weight loss programme

Participants randomised to the dietary weight loss programme arm will be given vouchers that exempt them from paying for 12 months of their local Weight Watchers meetings. Vouchers will be given in batches every 3 months. These will allow access to 12 sessions in the weekly meetings and to Weight Watchers online and mobile tools.

5.3. Compliance monitoring

Data on compliance in the bariatric surgery arm will be collected directly from the bariatric surgery team overseeing the surgery pathway. Compliance in this arm will be considered as undergoing the bariatric surgery. Reasons for non-compliance will be recorded.

Data on attendance to Weight Watchers for participants in the dietary weight loss programme arm will be self-reported.

5.4. Concomitant therapy

Participants may be taking acetazolamide therapy at entry into the trial. This should not affect trial outcome as randomisation to the two trial arms will be stratified by whether acetazolamide is being taken at entry or not. Other drugs used to treat IIH may also be taken by participants (e.g. analgesia, headache prophylaxis).

Apart from the trial treatments allocated at randomisation, all other aspects of patient management are entirely at the discretion of the local doctors.

5.5. Excluded medications or interactions

Significant co-morbidity, Cushing's syndrome, Addison's disease or the use of oral or injected steroid therapy (not contraception or topical or inhaled steroids) will exclude potential participants from the trial. Patients who have undergone optic nerve sheath fenestration will also be excluded from the trial as distortion of the optic nerve would prevent accurate assessment of their disease state.

Pregnancy will necessarily exclude potential participants as this is a contraindication for weight loss surgery. Potential participants will undergo a pregnancy test at screening; participants in the surgery pathway will also be tested for pregnancy before the procedure as a matter of routine care.

As weight loss is contraindicated during pregnancy and the outcome measures are linked to weight loss, pregnancy during the trial will distort data; thus any participants becoming pregnant during the trial will be excluded from further interventions but followed up as usual where possible. Trial assessments will be carried out at the earliest possible date post-partum for participants who become pregnant during the trial.

5.6. Withdrawal of treatment or protocol violation

If a participant does not receive their treatment or in any other way does not follow the trial protocol they will still be followed up and analysed on an intention to treat basis unless they choose to withdraw from the trial. Such protocol deviations and reasons for withdrawal will be recorded.

If participants randomised to the dietary weight loss arm fail to attend all of their Weight Watchers sessions this will not be considered a protocol violation, although attendance will be recorded (participant-reported) and described. As in other trials involving such an intervention, it is not expected that participants will attend every session; some may have less than 50% attendance (30% of participants attended less than 50% of sessions over 12 weeks in one trial [28] and we would expect a lower attendance rate over 12 months).

6. FOLLOW-UP AND ASSESSMENTS

6.1. Format of assessment visits

Participants will undergo a screening/baseline assessment (0 months). Participants will then be evaluated at 3, 6, 12, 24 and 60 months. Participants randomised to surgery will also be evaluated at approximately 1 month post-surgery.

6.2. Screening/baseline visit

The combined screening/baseline visit will be carried out according to the process shown in Figure 2 overleaf. If it is necessary (i.e. due to availability of required hospital staff or facilities or to make a shorter appointment to fit participant preferences or requirements), then the baseline visit may be split, and some assessments may be arranged for up to 30 days before randomisation provided that the full written informed consent process is complete before any assessments which are not part of routine clinical care are carried out.

To ensure the participant is eligible, the pregnancy test and LP (and related meal stimulation and post meal blood samples) must be done on the day of randomisation if the screening/baseline visit is split.

- **Visual measurements:** Measurements to be undertaken are the LogMAR (log of the minimum angle of resolution) chart to assess visual acuity, automated perimetry to measure the visual field mean deviation, an evaluation of contrast sensitivity using a MARS chart, and an Ishihara book to assess colour vision. The pupils will be dilated using 1% tropicamide (as is routinely done at clinic visits) and papilloedema will then be measured using spectral OCT.

  Papilloedema will be further graded centrally following fundus photographs. These will be compared after all participants have reached the primary endpoint by two neuro-ophthalmologists blinded to trial treatment arm. The assessors will score the paired papilloedema images as better/same/worse as per the methodology described in a previous study [33]. They will also assign a Frisén score to the images.

  If any of the visual assessments which are part of routine clinical care have been carried out within the last 30 days at the participant's outpatient clinic appointment they will not be repeated and the last recorded values will be used as baseline data. The fundus photography will, for the baseline assessments, be done at the pre-screening stage where practical to lessen the burden on participants on the main assessment day.

Figure 2: Baseline visit

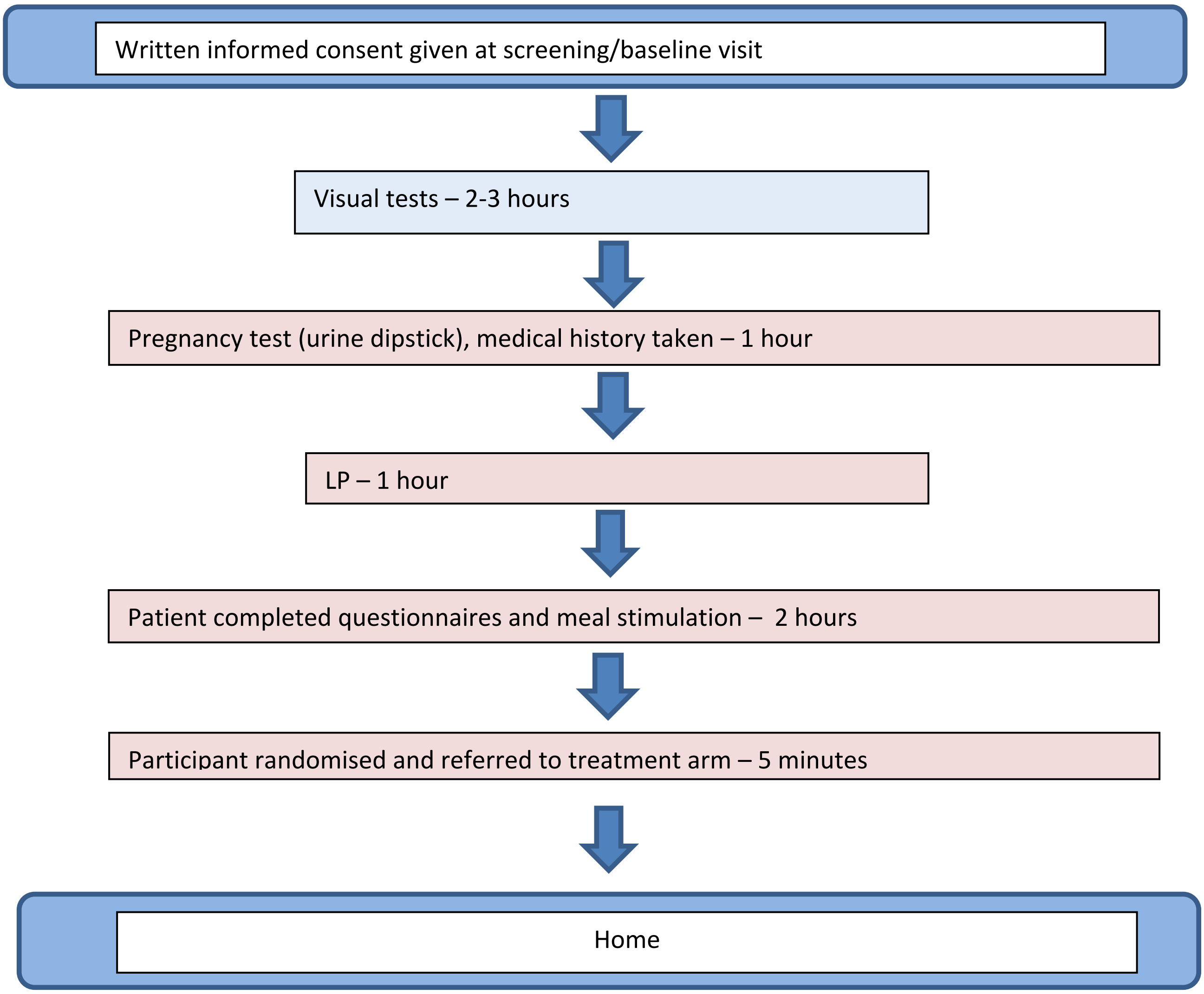

- **Pregnancy Test:** A urine pregnancy test will be done at the screening/baseline visit.

- **Clinical Data:** This visit will include recording of demographic data, and current medication (acetazolimide, topiramate, hormonal contraception, diuretics, anti-hypertensives and headache preventatives).

- **Clinical Measurements:** Blood pressure, waist and hip measures and ratio, height and weight (footwear removed) and body composition using Tanita scales.
  - Height will be measured to the nearest 0.1 cm with a rigid stadiometer
  - Body weight will be measured in light indoor clothing to the nearest 0.1 kg
  - Waist circumference will be recorded whilst the participant is supine to the nearest 0.1 cm at the mid-point between the lower costal margin and the level of the anterior superior iliac crest
  - Hip circumference will be recorded to the nearest 0.1cm, from the widest point of the hips and the maximum protrusion of the gluteal muscles.
  - Brachial blood pressure will be measured as recommended by the British Hypertension Society (http://www.bhsoc.org/how_to_measure_blood_pressure.stm) three times in the sitting position using standardised blood pressure monitors. The average of the second and third blood pressure readings will be recorded.

  The STOP-Bang screening tool [34, 35] will also be used to assess risk of sleep apnoea.

- **IIH Symptoms:** The presence or absence of symptoms attributed to IIH (and not from pre-existing conditions) will be formally recorded (pulsatile tinnitus, visual loss, diplopia (excluding that occurring from a longstanding squint), visual obscurations, and headache).

  Headache: Participants will complete a daily headache diary in the week before the baseline/screening visit (or retrospectively if not possible), which will record severity, duration and use of analgesia. Headache phenotype (according to criteria from the International Headache Society) will be assessed. Headache associated disability will be evaluated using the Headache Impact Test-6 score (HIT-6). Change in the headache severity following LP will also be evaluated using a pain scale (0-10). The participant will be asked to rate their headache severity immediately before the LP, and for the week following the LP (Post-LP Headache diary).

- **Venesection:** The participant will undergo fasted blood sampling for analysis as described in section 6.3 below. After the fasted blood samples they will take two standard fortisips (240mls) as a meal stimulation. An LP will be performed at least 30 minutes after the meal stimulation and a timed series of blood samples will be collected at 15, 30, 60, 90 and 120 minutes following the fortisips.

- **Lumbar Puncture (LP):** To avoid the LP affecting the visual measures it will be performed after the visual tests have been completed. ICP will be recorded in cmCSF. Image guidance may be used if necessary. Only participants with an LP pressure greater than 25cmCSF will be recruited. Both opening and closing ICP will be recorded. 8mls of CSF will be collected and stored for future biomarker analysis.

- **Patient rated outcome measures** will be completed whilst the participant is resting after the LP; this will reduce the time the participant is required in clinic (see section 6.8 below).

If the participant is eligible for the trial following screening and is recruited and randomised into the trial, the data collected at the pre-screening and screening visits will be used for the baseline data.

6.3. Follow up visits

The 3 and 6 month follow up assessments will monitor clinical measurements as in 6.2 above (see Table 1).

The 12, 24 and 60 month visits will follow a similar process to the baseline visit (see Figure 3 and Table 1), except that the pregnancy test will not be repeated. Visits will take place within a window of 1 month of the time point where possible.

6.4. Post-surgery visits

Those participants randomised to surgery will be invited to attend an assessment after their operation (visit window of 1-2 weeks post-op where possible). This is to measure gut neuropeptides (GLP-1) and investigate their role in the disease. At this visit the meal stimulation will be repeated with accompanying LP and pre- and 15, 30, 60, 90 and 120 minutes post-meal stimulation blood sampling. The Post-LP Headache diary will also be completed.

6.5. Withdrawal

Informed consent is defined as the process of learning the key facts about a clinical trial before deciding whether or not to participate. It is a continuous and dynamic process and participants should be asked about their ongoing willingness to continue participation.

Participants should be aware at the beginning that they can freely withdraw (discontinue participation) from the trial (or part of) at any time.

Types of withdrawal as defined are:

- The participant would like to withdraw from trial treatment, but is willing to be followed up in accordance with the schedule of assessments (i.e. the participant has agreed that data can be collected and used in the trial analysis).
- The participant would like to withdraw from trial treatment and does not wish to attend trial visits in accordance with the schedule of assessments but is willing to be followed up at standard clinic visits (i.e. the participant has agreed that data can be collected at standard clinic visits and used in the trial analysis, including data collected as part of long-term outcomes).
- The participant would like to withdraw from trial treatment and is not willing to be followed up in any way for the purposes of the trial and for no further data to be collected (i.e. only data collected prior to the withdrawal can be used in the trial analysis).

The details of withdrawal (date, reason where given and type of withdrawal) should be clearly documented in the source data.

6.6. Timing of assessments

Table 1 on page 30 summarises the outcome measures and assessments over the course of the trial.

6.7. Participant completed questionnaires

Participant completed questionnaires (EQ-5D-5L, SF-36 Version 1, ICECAP, Hospital Anxiety and Depression (HADS) score, Resource usage, Headache Impact Test-6, and Allodynia Symptom Checklist-12) will be completed by participants during their clinic visits at baseline and again at 12, 24 and 60 months. The Epworth Sleepiness Scale and Berlin Questionnaire will also be completed at baseline and 12 months to assess risk of sleep apnoea. A 7 day headache diary will be given to participants to complete at home at least a week before their scheduled clinic appointments.

6.8. Assessment methods

The CRFs will comprise the following forms:

- Visual Assessment Form: to be completed by the clinicians who will be carrying out the relevant standard clinical practice assessments or from their patient notes;

- Clinician Form; Research Nurse Form: to be completed by the named individuals on the delegation log who will be carrying out the relevant assessments and taking the relevant samples;

- Participant booklets including sleep questionnaires: comprising participant completed questionnaires as described above and to be completed by participants during their clinic visit;

- Headache diary: this will be supplied to participants a week before their clinic visits are due and will include analgesic use (a similar diary will be use to track headache severity after lumbar puncture);

  SAE Form: this will be completed by the Principal Investigator (PI) or delegated member of the research team when required. Please see the Adverse Event Reporting section of this protocol for details.

- Pre-Surgery Form; Surgery Form; Post-Surgery Form and Subsequent Procedure Form: these will record the bariatric surgery pathway and will be completed by the named individuals on the delegation log at surgery sites.

- Control "Light" form: there will be a specific CRF combining the assessments to be undergone by the subset of Matched Obese Control participants described in section 12.1.

Table 1: Outcome measures and assessments

**Outcome**	**Measure**	Pre-screening visit	Baseline	3 months	6 months	Post-op	(Primary endpoint) 12 months	24 months	60 months
Primary outcome									
Intracranial pressure	Lumbar puncture		x			x	x	x	x
Secondary outcomes									
Eligibility	Pregnancy test		x						
Weight	BMI		x	x	x	x	x	x	x
	Waist/hip ratio		x	x	x	x	x	x	x
	Blood pressure		x	x	x	x	x	x	x
	Body composition using Tanita scales		x	x	x	x	x	x	x
Visual assessments	Visual acuity and contrast sensitivity		x				x	x	x
	Humphrey visual field (24-2)		x				x	x	x
	Ishihara colour assessment		x				x	x	x
	Optical coherence tomography		x				x	x	x
	Retinal photographs	x					x	x	x
Headache assessments	Headache Impact Test 6	x					x	x	x
	Post-LP Headache diary		x			x	x	x	x
	Headache diary	x					x	x	x
Quality of Life	EQ-5D-5L		x				x	x	x
	ICECAP-A		x				x	x	x
	SF-36 Version 1		x				x	x	x
	Hospital Anxiety and Depression (HAD) score		x				x	x	x
	Allodynia Symptom Checklist-12		x				x	x	x
Health Economics	Cost-effectiveness, -utility and -benefit		x				x	x	x
Biomarkers	Blood		x			x	x	x	x
	CSF		x			x	x	x	x
	Meal stimulation		x			x	x	x	x
Sleep apnoea	Epworth Sleepiness Scale, Berlin questionnaire		x				x		
	STOP-Bang	x					x		
SAE monitoring	SAE forms		x	x	x	x	x	x	x

6.9. Blood and CSF samples

Serum and cerebrospinal fluid samples will provide data on disease biomarkers at baseline, 12, 24 and 60 months.

Venesection

The participant will undergo fasted blood sampling for (identifying tube colours as used by UHB):

- Fasting metabolic evaluation (for real time analysis):
  - HbA1c – 1 purple tube of blood.
  - Glucose and Lipids (Cholesterol, triglycerides, HDL) - 1 grey tube of blood, 1 yellow tube of blood
- PCOS bloods (for real time analysis):
  - Testosterone – 1 yellow tube of blood
  - SHBG, Androstendione, DHEAs, FSH, LH, Oestrodiol, 17OHP [hydroxyprogestrogen] - 1 red tube of blood
- Exploratory analysis:
  - Biomarker analysis including fasting insulin – 1 yellow tube of blood
  - GLP-1 - 1 purple pre-prepared GLP-1 tube of blood (provided to the site and containing a dipeptidyl peptidase-4 [DDP-4] inhibitor, frozen in lab and brought to clinic in an ice bucket before the participant arrives) taken and kept on ice before processing and storage.

The participant will then undergo a meal stimulation test, taking two standard fortisips. Further samples of blood will be taken approximately 15, 30, 60, 90, and 120 minutes after this test and collected in 5 purple pre-prepared GLP-1 tubes as above.

The samples and quantities of blood taken at the various visits are summarised in table 2 and 3 below:

Table 2: Blood samples and tubes (baseline, 12, 24 and 60 months)

Fasting metabolic evaluation	PCOS	Exploratory analysis	Pre and Post meal samples
1 purple tube, 1 grey tube, 1 yellow tube	1 yellow tube, 1 red tube	1 yellow tube	6 purple GLP-1 tubes

Table 3: Blood samples and quantities (baseline, 12, 24 and 60 months)

Yellow tubes (4mls)	Grey tubes (2mls)	Purple tubes (4mls)	Purple GLP-1 tubes (2mls)	Red tubes (6mls)	Volume collected in mls
3	1	1	6	1	36

Lumbar puncture

The participant will undergo a lumbar puncture approximately 30 minutes after the meal stimulation test.

1ml of CSF will be collected for microscopy +/- culture. Approximately 1ml will be collected into 3 tubes (6 drops in each) for glutamate, substance P and calcitonin gene related peptide (CGRP) analysis. Approximately 8mls of CSF will be collected in a universal tube containing a DPP-4 inhibitor and kept on ice before processing.

6.10. Processing and storage of samples

Full details of sample processing are described in the separate trial laboratory manual.

Blood

- Fasting metabolic bloods: 1 purple tube and 1 grey tube will be processed by the hospital laboratories.
- PCOS bloods: 1 yellow tube and 1 red tube will be processed by the hospital laboratories.
- Exploratory analysis and pre/post meal samples: 1 yellow tube and 6 purple GLP-1 tubes will be processed according to the laboratory manual and the aliquots stored at -80°C, initially at the site before transfer to UoB.

CSF

- Microscopy +/- culture: 1ml of sample will be processed by the hospital laboratories.
- Approximately 1ml of sample (3 tubes of 6 drops each) will be transferred on dry ice to a -80°C freezer before transfer to UoB for glutamate, substance P and CGRP analysis.
- The remaining CSF will be processed according to the laboratory manual before storage at -80°C in the hospital and subsequent transfer to UoB for biomarker analysis including GLP-1.

7. ADVERSE EVENT REPORTING

FAX SAE forms to the IIH:WT Trial Office on:
0121 415 9135 or email neuroscience@trials.bham.ac.uk

7.1. Assessment of Safety

There are no novel medical devices or Investigational Medicinal Products (IMPs) used as part of this trial.

The main risks in the trial are the bariatric surgery, as mentioned in sections 1.4 and 1.5.2, and the LP performed at baseline, post-operative visit, 12, 24 and 60 months.

Serious Adverse Events (SAEs) will be reported on a trial-specific SAE form and will follow the procedure/timeframes outlined in this section of the protocol.

7.2. Serious Adverse Events

SAEs are any untoward medical occurrence or effect that:

- Results in death
- Is life threatening
- Requires hospitalisation or prolongation of an existing hospitalisation[‡]
- Results in persistent or significant disability/incapacity or
- Is a congenital anomaly or birth defect, or
- Is otherwise considered medically significant by the Investigator.

SAEs may occur following randomisation or the screening tests required prior to randomisation. The SAE reporting period will end 30 days after the participant's last trial assessment at 5 years (lumbar puncture).

[‡] Hospitalisation is defined as an unplanned, overnight, formal inpatient admission, even if the hospitalisation is a precautionary measure for continued observation. Thus hospitalisation for protocol treatment, elective procedures (unless brought forward due to worsening symptoms), social reasons, or logistical reasons are not regarded as a SAE. Further examples of hospitalisation not constituting an SAE are provided in section 7.6.

7.3. SAE reporting procedures

All SAEs will be recorded on a SAE form and in the participant medical notes. The SAE form must be reported to the trial office within 24 hours of the site being made aware of the event. When completing the form, the local PI (or delegate) will assess the severity and causality of the SAE. It is the PI's responsibility to report SAEs to the trial office and to their Trust's R&D department (if this reporting is required by the Trust).

Completed SAE forms should be faxed to the Neuroscience trial office on 0121 415 9135 or emailed to neuroscience@trials.bham.ac.uk. The Investigator at site will be required to respond to any related queries raised by the trial office as soon as possible.

On receipt the Trial Office will allocate each SAE a unique reference number which will be sent to the site as proof of receipt. If confirmation of receipt is not received within 1 working day please contact the Trial Office. The SAE reference number should be quoted on all correspondence and follow-up reports regarding the SAE and filed with the actual SAE in the Site File.

For SAE Forms completed by someone other than the PI, the PI will be required to countersign the original SAE Form to confirm agreement with the causality and severity assessments. The form should then be sent to the Trial Office and a copy kept in the Site File.

Following reporting of an SAE for a participant, the participants should be followed up until resolution or stabilisation of the event. Follow-up information should be provided using the SAE reference number provided by the BCTU trials team.

7.4. Assessment of relatedness

The following categories, as outlined in Table 4 below, will be used to define the relatedness (causality) of the SAE.

Table 4: categorisation of relatedness

Category	Definition	Causality
Definitely	There is clear evidence to suggest a causal relationship, and other possible contributing factors can be ruled out.	Related
Probably	There is evidence to suggest a causal relationship, and the influence of other factors is unlikely.	
Possibly	There is some evidence to suggest a causal relationship. However, the influence of other factors may have contributed to the event (e.g. the patient's clinical condition, other concomitant events or medication)	
Unlikely	There is little evidence to suggest there is a causal relationship. There is another reasonable explanation for the event (e.g. the patient's clinical condition, other concomitant events or medication).	Unrelated
Not related	There is no evidence of any causal relationship.	

On receipt of an SAE Form, the CI (or designee) will independently review the severity and causality of the SAE. An SAE judged by the PI or CI to have a reasonable causal relationship with the intervention will be regarded as a related SAE. The causality assessment given by the PI will not be downgraded by the CI. If the CI disagrees with the PI's causality assessment, the opinion of both parties will be documented, and where the event requires further reporting, the opinion will be provided with the report.

7.5. Assessment of expectedness

Expectedness will be assessed by the CI (or designee) using this trial protocol as the reference document to assess SAEs. Table 5 overleaf gives definitions of expectedness with respect to SAEs.

If the event is unexpected (i.e. is not defined in the protocol as an expected event) it will be classified as an unexpected SAE.

Table 5: categorisation of expectedness

Category	Definition
Expected	An adverse event that is consistent with known information about the trial related procedures or that is clearly defined in this protocol.
Unexpected	An adverse event that is not consistent with known information about the trial related procedures.

7.6. Expected Adverse Events

Further to the definition of hospitalisation in section 7.2, an overnight hospital stay after surgery **will not be counted as an SAE** if required for routine care and is not due to a surgical complication. Reasons may include, for example, when a patient has a lengthy journey home, time of surgery (late in afternoon), lack of carer, when the patient has no telephone, Insulin treated diabetes, previously diagnosed sleep apnoea, ASA grade 3[§] or more, i.e. it is a clinical decision to stay overnight. An overnight hospital stay for any inflation of the gastric band, whether radiological or clinical, **will not be counted as an SAE** as it is a part of routine care.

Expected Adverse Events include:

- Admission for deterioration of IIH;
- Admission for post-LP headache.

Expected surgical Adverse Events include:

- Admission for regurgitation;
- Admission for full band deflation or band slippage;
- Admission for surgical revision;
- Conversion from laparoscopic to open surgery.

7.7. Related and Unexpected SAEs

The CI will undertake urgent review of all SAEs and may request further information immediately from the clinical team at site. The CI will not overrule the severity or causality assessment given by the site Investigator but may add additional comment on these. The CI will assess the Expectedness of the SAE. Related and Unexpected SAEs will be notified to the REC using the standard National Research Ethics Service SAE report form for non-CTIMPs within 15 days.

7.8. Annual Progress Reports

An annual progress report (with safety information included) will be submitted to the REC within 30 days of the anniversary date on which the favourable opinion was given, and annually until the trial is declared ended. Progress Reports will also be submitted to the Funder in accordance with their requirements.

7.9. Reporting urgent safety measures to the REC

If any urgent safety measures are taken BCTU shall immediately, and in any event no later than 3 days from the date the measures are taken, give written notice to the REC of the measures taken and the circumstances giving rise to those measures.

7.10. Data Monitoring Committee

The independent Data Monitoring Committee (DMC) will review all SAEs.

[§] ASA Grade 3 is defined as a patient with severe systemic disease.

7.11. Notification of Serious Breaches of GCP and/or the protocol

A Serious Breach is an event which is likely to effect to a significant degree:

- the safety or physical or mental integrity of the participants of the trial; or
- the scientific value of the trial.

The BCTU on behalf of the Sponsor shall notify the REC in writing of any serious breach of:

- the conditions and principles of GCP in connection with the trial; or
- the protocol relating to the trial, within 7 days of becoming aware of that breach.

The Sponsor will be notified immediately of any case where the above definition applies during the trial.

8. DATA MANAGEMENT AND VALIDATION

8.1. Source Data

In order to allow for the accurate reconstruction of the trial and clinical management of the subject, source data will be accessible and maintained. Source data is kept as part of the participants' medical notes generated and maintained at site. Source data is all information in original records and certified copies of original records of clinical findings, observations, or other activities related to the trial.

The CRFs are not the source data, although there are exceptions: the below will be considered source data:

- the patient completed questionnaires (see section 6.8);
- the answer sheets provided for administering the cognitive function tests (see section 11);
- the save files generated by the cognitive function tests and sleep apnoea monitoring devices (see sections 11 and 12);
- the patient rated score on the neurophysiology CRF (see section 11).

8.2. Confidentiality of personal data

This trial will collect personal data about participants. Participants will be informed about the transfer of this information to the trial office at BCTU, and will be asked to consent to this. The data will be entered onto a secure computer database built, hosted and maintained by BCTU according to University and BCTU security and quality policies and procedures. Access to the online trials system is via a secure encrypted connection and is restricted to authorised users who have a username and secret password. Functionality on the application is restricted based on the user's role. A full audit log of all changes to trial data is maintained automatically by the system. BCTU servers are protected by physical and electronic access security measures. The servers are kept in a locked air conditioned server room in the BCTU. Server access is restricted to named individuals in security groups, with user rights limited to what is needed for their role. Data is automatically backed up each night to the College of Medicine and Dentistry file share, and then onto tapes which are kept in a fire proof safe.

Any data to be processed outside BCTU will be anonymised. All personal information obtained for the trial will be handled and stored in accordance with the Data Protection Act 2018 and the EU General Data Protection Regulation 2018, held securely, and treated as strictly confidential.

With the participant's consent, their date of birth and NHS number will be collected to assist with long-term follow-up. Participants will be identified using only their unique trial number and date of birth in mmm/yyyy format on CRFs and correspondence between the Trial Office and sites.

The patient consent form, which will be sent to BCTU will, out of necessity, contain identifiable personal data. These will be stored separately from the study record. The consent form will be sent to BCTU, with the patient's consent, to monitor that the consent documentation has been completed correctly.

Samples will be stored as described in section 6.10 above. They will be identified by a unique identifier, visit number, and a code describing the sample. This will be recorded on a Sample Log at each visit.

Investigators will keep their own trial file logs which link participants with anonymised CRFs. The Investigator must maintain documents not for submission to the Trial Office (e.g. Participant Identification Logs) in strict confidence. In the case of specific issues and/or queries from the regulatory authorities, it will be necessary to have access to the complete trial records, provided that participant confidentiality is protected.

The Trial Office will maintain the confidentiality of all participant data and will not disclose information by which participants may be identified to any third party other than those directly involved in the treatment of the participant and organisations for which the participant has given explicit consent for data transfer. Representatives of the IIH:WT trial team may be required to have access to patient notes for quality assurance purposes but participants should be reassured that their confidentiality will be respected at all times.

All staff involved in the IIH:WT trial (clinical, academic, BCTU) share the same duty of care to prevent unauthorised disclosure of personal information. No data that could be used to identify an individual will be published.

8.3. Long-term storage of data

In line with Medical Research Council guidelines and the Medicines for Human Use (Clinical Trials) Regulations, once data collection is complete on all participants, all data will be stored for at least 20 years. Any queries or concerns about the data, conduct or conclusions of the trial can also be resolved in this time. Limited data on the participants and records of any adverse events may be kept for longer if recommended by an independent advisory board.

Trial data will be stored within the BCTU under controlled conditions for at least 3 years after closure. Long-term offsite data archiving facilities will be considered for storage after this time. The BCTU has standard processes for both hard copy and computer database legacy archiving. Archiving will be authorised by the BCTU on behalf of University of Birmingham following submission of the end of trial report.

PI's are responsible for the secure archiving of essential trial documents (for their site) as per their NHS Trust policy. All essential documents will be archived for a minimum of 5 years after completion of trial.

8.4. Data management

The IIH:WT trial will not use double data entry. Data is validated by pop-ups on the database when out of range and by random checks. All data entry will be done by BCTU staff. All missing and ambiguous data will be queried using Data Clarification Forms (DCFs). Responses should be made on the DCF. The original DCF should be copied and the copy attached to the CRF to which it relates. The DCF should be returned to the trial office. A separate data management document will be created by the trial office.

8.5. Definition of the End of Trial

The end of trial will be 1 month after the last data capture-related query is resolved. The last data capture will be 60 months following recruitment of the last participant.

The BCTU trial team will notify the REC and Sponsor that the trial has ended and a summary of the clinical trial report will be provided within 12 months of the end of trial.

9. STATISTICAL CONSIDERATIONS

9.1. Sample size

Total n=64. 32 participants in each arm (bariatric surgery versus diet).

For this study we are hypothesising that the greater weight loss anticipated in the bariatric surgery arm compared to the dietary weight loss arm will consequently reduce the ICP further in the bariatric arm than in the dietary weight loss arm. Bariatric surgery patients typically lose 31% ± 3% of body weight by 12 months [13]). A weight loss of 15.3% ± 7.0% of body weight over 3 months was achieved by patients following a low calorie diet [10]. Data from this study showed that ICP was significantly reduced by 20% (ICP at baseline in 20 IIH patients was 39.8 ± 5.1 cmCSF and ICP was reduced by 8 ± 4.2 cmCSF, p<0.001).

If we assume a conservative change of ICP in the bariatric surgery arm to that previously observed of 8 cmCSF and a change of 3cmCSF in the dietary weight loss arm (a value to reflect changes slightly greater than the baseline fluctuations seen in our previous study), then we are looking to detect a mean difference of 5cmCSF between the groups. To detect this difference of 5cmCSF with 90% power and alpha=0.05 using a 2-sided t-test (assuming a standard deviation of 5.1 [10]) requires a total of 46 patients (23 per arm). If we allow for a 28% drop out rate, then we will need to recruit 32 patients per arm, 64 patients in total.

We believe that the SD of 5.1 is a true reflection of the variability of the data as this is taken from the baseline measurements from our previous study, which is a similar population to that being recruited into this study [10]. However, this assumption for the sample size calculation will be monitored during the trial as part of the interim analyses.

9.2. Projected accrual and attrition rates

Recruitment for our previous study with very similar inclusion criteria was at a rate of 1.5 participants per month [10]; we consequently feel that the recruitment target of 1.5 participants per month (64 participants over 45 months) is realistic and achievable. Attrition rates for this treatment and patient group is not known; we have allowed a 28% rate of drop out.

9.3. Statistical Analysis

A separate Statistical Analysis Plan for the IIH:WT trial will be produced and will provide a more comprehensive description of the planned statistical analyses for the primary and secondary outcome measures. A brief outline of these analyses is given below.

The primary comparison groups will be composed of those randomised to the bariatric surgery arm and those randomised to the dietary weight loss arm. In the first instance, all analyses will be based on the intention to treat principle, i.e. all patients will be analysed in the treatment group to which they were randomised irrespective of compliance with the randomised allocated treatment or other protocol violation. For all major outcome measures, summary statistics and differences between groups (e.g. mean differences, relative risks) will be reported, with 95% confidence intervals and p-values from two-sided tests also given. Outcomes will be adjusted for the stratification variable listed in section 4.6. For all analyses, a p-value <0.05 will be considered statistically significant and there will be no adjustment for multiple testing.

Primary Outcome Analysis

The primary outcome will assess the ICP at 12 months. Data will be reported with means and standard deviations or medians and ranges for non-parametric data. The ICP at 12 months for the two study arms will be compared using a linear regression model with baseline ICP and acetazolamide use at entry (stratification variable) included as covariates in the model.

Secondary Outcome Analyses

Secondary outcome measures include a mixture of continuous and categorical data items. Continuous outcomes (e.g. quality of life) will be analysed as per the primary outcome measure. Categorical outcomes

(e.g. presence or absence of symptoms, number of CSF shunting referrals) will be expressed as the number and percentage of patients experiencing these outcomes in the two groups. Log-binomial models will be used to compare the data between the two study arms, with baseline data (where available, i.e. baseline symptom data) and acetazolamide use at entry (stratification variable) included in the model as covariates.

9.4. Missing Data and Sensitivity Analyses

Our primary analysis will be by intention to treat using complete cases. Where data are missing, we will perform sensitivity analyses to assess how different reasons for the missing data might have impacted upon the results. Sensitivity analyses will include adopting a "baseline value carried forward approach" (i.e. assume no change in ICP for drop-outs). For more details regarding the sensitivity analyses, please refer to the Statistical Analysis Plan.

9.5. Subgroup Analyses

The randomisation will be stratified according to whether or not participants are taking acetazolamide or not at entry into the trial to ensure balance across the two treatment arms. There are no planned subgroups analyses for this trial.

9.6. Interim Analyses

Interim analyses of efficacy and safety are planned annually. These interim analyses will be reviewed by the independent Data Monitoring Committee (DMC) on an annual basis or more frequently if required by the DMC or Trial Steering Committee. A DMC report and charter outlining the terms of reference (including information on stopping rules) will be agreed with the DMC. See section 13.3 for further information on the DMC.

9.7. Final Analyses

The first analysis of the main trial data for publication will be completed once every patient has completed 12 months follow-up. The final analysis for the IIH:WT trial will occur once the last randomised patient reaches the 5 year follow-up assessment.

10. HEALTH ECONOMIC OUTCOMES

10.1. Health economic outcomes

The following analysis will be undertaken:

Cost-effectiveness analysis – Primary trial outcome: ICP measured at baseline and 12 months will be evaluated in terms of cost to reduce the ICP by 12.5%. This will inform the cost-effectiveness analysis and information will come from the trial data.

1. **Cost-utility analysis** – Utility data collected at baseline and 12 months using the EQ-5D-5L and ICECAP-A questionnaires. The utility information from the responses to this questionnaire will be used to estimate Quality-Adjusted Life Years (QALYs).

2. **Cost-benefit analysis** – Monetary outcomes will be measured using the 'Willingness to Pay' (WTP) method. A WTP question will be asked at baseline and at 12 months in both cohorts of participants (surgery and dietary weight loss programme groups). The question will ask for WTP for treatment before and after the treatment takes place hence will ask for values from both an ex-ante and an ex-post perspective.

10.2. Overall objective

The overall objective of the economic component of this trial will be to measure the costs and outcomes to assess the cost-effectiveness of bariatric surgery and dietary weight loss programme for treatment of IIH.

Specific objectives:

1. To measure the costs from both a health care and a societal perspective.
2. To apply the WTP method from both an ex-ante (before intervention) and ex-post (after intervention) perspective.
3. To measure the productivity costs associated with having IIH and the impact the intervention has on these productivity costs.
4. To use the EQ-5D-5L as an outcome measure to derive QALYs.
5. To use the ICECAP-A as an outcome measure for capabilities.
6. To conduct a cost-utility analysis using QALYs (derived from EQ-5D-5L) as the outcome.
7. To conduct a cost-effectiveness analysis using ICP as the primary outcome.
8. To conduct a cost-benefit analysis by incorporating productivity costs and using WTP values as the unit of outcome.

10.3. Methods

Cost data collection

Primary data on costs and resource use will be collected prospectively alongside the trial. The process of collecting resource use data will be undertaken separately from data collection on unit costs. Table 6 overleaf summarises the type of resource use, method of collection and timing of collection within the trial.

The costing will be divided up into the measurement of health service costs and costs associated with productivity loss related to IIH. Productivity loss associated with IIH will be measured by estimating the rate of absenteeism (days of work missed because of illness) and presenteeism (days at work but limited in performing job tasks because of ill health). The productivity loss associated with IIH will be directly compared for the surgical cohort versus the diet cohort.

For the health service resource use, unit costs will be obtained and attached to the resource use items to estimate patient-specific costs. Unit costs will be obtained from published sources.

Outcome data collection

Four types of outcome data will inform the economic analysis and will determine the type of economic evaluation undertaken:

For cost-effectiveness analysis - Primary study outcome: ICP measured at baseline and 12 months. This will inform the cost-effectiveness analysis. This information will come from the trial data.

For cost-utility analysis – utility data collected at baseline and 12 months using the EQ-5D-5L and ICECAP-A questionnaires. The utility information from the responses to this questionnaire will be used to estimate QALYs.

For cost-benefit analysis – monetary outcomes will be measured using the WTP method. A WTP question will be asked at baseline and at 12 months in both cohorts of participants (surgery and dietary weight loss programme groups). The question will ask for WTP for treatment before and after the treatment takes place hence will ask for values from both an ex-ante and an ex-post perspective.

ICECAP-A – capabilities outcomes will be measured at baseline and at 12 months in both cohorts and will feed into a wider perspective analysis therefore will be part of cost-benefit analysis.

Table 6: Health economics data collection

Cost item	Resources Used	Collection method	Timing	Resource use collection instrument
Costs of surgery (health service costs)	Pre-operative:<br>Outpatient visits;<br>Dietician consultations;<br>psychologist appointments.	Pre-op:<br>Pre-op Form will be completed by Trial team by audit of hospital notes.	This information will be collected as an ongoing process throughout the trial.	Data collection by audit of hospital notes by Trial team.
	Surgery:<br>Theatre time; Length of hospital stay; length of stay in ITU; length of stay in HDU.	Surgery data will be collected on Surgery Form completed by audit of hospital notes		
	Conversion rate from laparoscopic to open surgery and Complications / revisions: Mortality; incisional hernias; apronectomy; repeat surgery.	Complications data will be collected on Surgery Form completed by audit of hospital notes		
	Post-discharge and general health service costs:<br>GP visits, practice nurse visits, district nurse visits.<br>Outpatient visits, dietician contacts, psychology consultations.	Post-discharge:<br>Outpatient activity collected by hospital audit.<br>GP visits and outpatient appointments collected by participant questionnaire.	The post-discharge data and general health service costs will be collected by participant questionnaire at 12, 24 and 60 months.	Participant questionnaire.
Costs of Weight Watchers (health service costs)	Unit cost of joining the Weight Watchers programme.	£48.50+VAT per 3 months	This will be recorded with each batch of Weight Watchers vouchers handed to participants.	Trial information (for Weight Watchers cost).
	General health service costs:<br>GP visits, practice nurse visits, district nurse visits.<br>Outpatient visits.	Participant questionnaire.	The general health service costs will be collected by participant questionnaire at 12, 24 and 60 months.	Participant questionnaire.
Productivity costs	Absenteeism: Number of days of work missed because of IIH.<br><br>Presenteeism: Number of days at work but limited in performing work-related tasks.	Participant questionnaire.	Baseline; 12 months; 24 months; 60 months.	Participant questionnaire.

10.4. Economic evaluation

Trial-based analysis

The first stage will be a within-trial cost-effectiveness analysis based on the outcome of 5, 10%, 20 and 30% reduction in ICP. The secondary outcome will be QALYs. A decision-tree model will be used to conduct the within-trial analysis. The analysis will adopt an incremental approach in that data collection will concentrate on resource use and outcome differences between the two trial arms. Appropriate one-way and multi-way deterministic sensitivity analysis will be carried out to test the robustness of the results.

Beyond the trial period

The results of the trial-based analysis will feed into a longer-term Markov decision analytic model if the trial-based analysis suggests a significant impact as a result of the bariatric surgery. If this is the case, the results of the trial-based model will be extrapolated beyond the trial period by using a Markov simulation model that will estimate health gains and cost-effectiveness over a lifetime. Data to populate this longer-term model will come from published sources that will be subject to quality criteria. Costs and benefits will be discounted at 3.5%. The economic analysis will be presented using cost-effectiveness acceptability curves to reflect sampling variation and uncertainties in the threshold cost-effectiveness values where appropriate. The robustness of the results will be explored using sensitivity analysis.

11. EXPLORATORY SUB-STUDIES

There are a number of optional exploratory sub-studies. These are detailed in sections 11 and 12.

11.1. Exploratory Aims

Sleep apnoea

- To evaluate the relationship of Obstructive Sleep Apnoea (OSA) to visual function in participants with IIH.
- To evaluate the impact of weight loss on OSA.

Metabolic syndrome

- To evaluate changes in Framingham cardiovascular disease score and metabolic parameters between baseline and 12 months.
- To evaluate changes in insulin sensitivity and lipids between baseline and 12 months.
- To evaluate changes in the Utah Early Neuropathy Score, peripheral nerve fibre conduction and intraepidermal nerve fibre density between baseline and 12 months.

Magnetic resonance imaging (MRI)

- To evaluate changes in venous sinus compression observed on magnetic resonance venography between baseline and 12 months.

Cognitive function

- To evaluate changes in cognitive function between baseline and 12 months.
- To evaluate the relationship between cognitive function and headache disability scores / index, depression scores, sleep apnoea scores, ICP and BMI.

Matched obese control group

- To evaluate the baseline difference in ICP, visual function, headache disability, sleep apnoea, cognitive testing, features of the metabolic syndrome, and biomarker analysis between IIH participants and a matched obese control cohort.

MRI test run group

- To validate the novel MRI scan sequences being used in the MRI sub-study above.

Biomarkers

- To evaluate the changes in hormonal, inflammatory, oxidative stress and neuropeptide biomarkers between baseline and 12 months.

11.2. Exploratory Outcome Measures

- Change in apnoea-hypopnoea index from 0 to 12 months
- Change in markers of peripheral neuropathy and metabolic syndrome from 0 to 12 months
- Change in MRI (including venous stenoses) from 0 to 12 months
- Change in cognitive function from 0 to 12 months
- Change in biomarkers from 0 to 12 (and 24 and 60) months
- Comparison between IIH patients and the matched control group at baseline with regards to apnoea-hypopnoea index, peripheral neuropathy and metabolic syndrome (including allodynia), MRI, cognitive function, and biomarkers
- Change in MRI over a double baseline period of healthy controls.

11.3. Changes to participant pathway to incorporate sub-studies

The participant pathway at sites taking part in any of the sub-studies will vary from that described in section 6.1 to accommodate the exploratory outcomes. At pre-screening, sub-study participants may additionally be asked to:

- Give a 24 hour urine sample (which will be used in the analysis of biomarkers if they subsequently consent to join the trial) – a urine bottle will be provided, and the urine sample should be completed the day before the appointment; and
- Return home with a sleep apnoea home study device to record two nights of their sleep data (which will be used together with the sleep questionnaires in the sleep apnoea sub-study if they subsequently consent to join the trial).

The screening/baseline day and the 0, 12, 24 and 60 month visits will vary to accommodate the exploratory outcomes as shown in table 7 overleaf:

Table 7: Outcome measures and assessments showing all optional exploratory outcomes

Outcome	Measure	Pre-screening visit	Baseline	3 months	6 months	Post-op	(Primary endpoint) 12 m	24 months	60 months
Primary outcome									
Intracranial pressure	Lumbar puncture		x			x	x	x	x
Secondary outcomes									
Eligibility	Pregnancy test		x						
Weight	BMI		x	x	x	x	x	x	x
	Waist/hip ratio		x	x	x	x	x	x	x
	Blood pressure		x	x	x	x	x	x	x
	Body composition using Tanita scales		x	x	x	x	x	x	x
Visual assessments	Visual acuity and contrast sensitivity		x				x	x	x
	Humphrey visual field (24-2)		x				x	x	x
	Ishihara colour assessment		x				x	x	x
	Optical coherence tomography		x				x	x	x
	Retinal photographs	x					x	x	x
Headache assessments	Headache Impact Test 6	x					x	x	x
	Post-LP Headache diary		x			x	x	x	x
	Headache diary	x					x	x	x
Quality of Life	EQ-5D-5L		x				x	x	x
	ICECAP-A		x				x	x	x
	SF-36 Version 1		x				x	x	x
	Hospital Anxiety and Depression (HAD) score		x				x	x	x
	Allodynia Symptom Checklist-12		x				x	x	x
Brain imaging	Magnetic resonance venography		x				x		
Health Economics	Cost-effectiveness, -utility and -benefit		x				x	x	x
Biomarkers	Blood		x			x	x	x	x
	24 hours urine sampling	x					x		
	CSF		x			x	x	x	x
	Meal stimulation		x			x	x	x	x
Sleep apnoea	Home based sleep studies	x					x		
	Epworth Sleepiness Scale, Berlin questionnaire	x					x		
	STOP-Bang		x				x		
Cognitive testing	Verbal working memory test, Attention Network Test – Interactions (ANT-I), Sustained attention test etc.		x				x		

Neurophysiology testing	Neuropathy screen, allodynia screen, basic electrophysiology and punch biopsy	x				x		
Control group	Full baseline assessments	x						
SAE monitoring	SAE forms	x	x	x	x	x	x	x

11.4. Sleep apnoea observational cohort sub-study

An association between OSA and IIH is well documented, although prevalence is unknown and causality has not been demonstrated [36]. Recent interest has focused on the role of OSA, with resulting intermittent hypoxia, in exacerbating microscopic angiopathies such as diabetic retinopathy [37]. OSA has also been associated with optic nerve ischaemia in glaucoma and non-arteritic ischaemic optic neuropathy [38]. We suggest that OSA may exacerbate optic nerve infarction resulting from papilloedema in IIH, and represent a risk factor for developing visual loss. Therefore an observational cohort study will be conducted with assessments of OSA at baseline and at 12 months.

Objectives for sleep apnoea sub-study

Primary objectives:

- To evaluate the relationship of the apnea-hypopnea index to visual function in patients with IIH.

Secondary objectives:

- To evaluate the impact of weight loss through either bariatric surgery or dietetic intervention on the apnea-hypopnea index.

Method for sleep apnoea sub-study

Participants will be assessed for sleep apnoea at baseline and 12 months (obese controls at baseline only). A member of the local research team will explain to the participant how to use the sleep observation device, which the participant will then take home. It will be programmed by the research nurse to record a set period of 12 hours, and the participant will monitor their sleep over 2 nights. The night which provides the most complete data will be assessed. Sleep studies will be scored in accordance with the American Academy of Sleep Medicine guidelines [39]. Where both nights' sleep studies provide <4 hours of adequate recordings they will be repeated if possible and if the quality remains poor they will be excluded from analysis.

An apnea-hypopnea index score of (AHI) ≥ 5 events/hour will be considered consistent with OSA diagnosis. OSA severity will be assessed based on the AHI, oxygen desaturation index (the number of oxygen desaturations of ≥ 4% per hour) and lowest oxygen saturation. OSA will be classified into mild, moderate and severe based on AHI ≥5, 5-14, 15-29, and ≥30 events/hour respectively.

The data will be scored by a sleep specialist blinded to the participant's treatment arm and quality controlled by a second specialist in sleep medicine by checking a subset of the data.

11.5. Metabolic syndrome sub-study

Our preliminary (currently unpublished) data indicates that patients with IIH (n=29) have features of metabolic syndrome including increased waist circumference (106.5±10.2cm), increased Homeostasis Model Assessment scores (2.1±2.1) (normal scores are less than 1), elevated fasting insulin (14.3±6.4μU/ml) and glucose: insulin ratios (0.41±0.20), with the latter two variables being significantly higher than in a cohort of matched obese controls (p=0.036 and p=0.027 respectively). These results suggest that IIH patients may be at increased risk of developing diabetes and cardiovascular disease in later life. Consequently, morbidity in IIH may extend beyond headaches and visual loss.

Peripheral neuropathy (PN) is a common complication of diabetes, but has also been linked to the metabolic syndrome [40], more specifically pre-diabetes [41] and hypertriglyceridaemia [42]. PN is disabling

as it produces pain and discomfort in the lower limbs which may progress to weakness and sensory loss, resulting ultimately in difficulties with balance and gait. PN may recede with appropriate dietary, lifestyle, and exercise interventions as suggested in patients with pre-diabetes [43]. The prevalence of PN has to our knowledge not previously specifically been evaluated in patients with IIH, but may contribute to morbidity with this population.

We propose as part of the current trial to evaluate the presence of co-existing PN in patients with IIH and to evaluate the effects of the interventions of this trial on objective markers of PN in the participants.

Objectives for metabolic syndrome sub-study

Primary objective:

- To evaluate the presence of PN and metabolic syndrome in patients with IIH.

Secondary objectives:

- To evaluate the impact of weight loss through either bariatric surgery or dietetic intervention on objective markers of PN.

Method for metabolic syndrome sub-study

Participants will undergo a clinical neuropathy screen using the Utah Early Neuropathy Score (UENS) [44] as part of the baseline assessment and the 12 month assessment (obese controls at baseline only). This score is appropriate in this setting as it has been validated in subjects with diabetes and pre-diabetes, is easy and quick to perform, and allows detection of mild cases of PN. The UENS requires a basic routine neurological examination assessing the strength, sensation and reflexes. This takes about 10 minutes.

In addition participants will undergo basic electrophysiology (nerve conduction studies) which will provide objective measurement of large nerve fibre function, which may be impaired in PN. This requires electrical pulses delivered over the surface of the skin with recordings performed also over the skin surface, in upper and lower limbs to study 2 motor nerves (unilateral common peroneal and tibial in their lower leg segments) and 4 sensory nerves (bilateral radial and bilateral sural). These may cause mild discomfort or tingling, but are not generally considered painful nor accompanied by adverse effects. This will take about 10 minutes. The range of values used to define normal response will be those available from recent literature using similar equipment [45].

Finally, as PN may result in damage exclusively to the small nerve fibres which cannot be detected by electrophysiology, we intend to perform a 3mm punch skin biopsy at the lower leg with appropriate sterile technique [46]. This is performed under a local anaesthetic and consequently is not painful. The procedure takes about 15 minutes in total. The superficial skin sample collected will then be studied for the intraepidermal nerve fibre density which is a marker of small nerve fibre function.

Alongside the screening for PN we will also measure anthropometric measurements (BMI, waist/hip and body composition using Tanita scales); Framingham Risk Score [**]; and take bloods (fasting glucose, insulin, cholesterol and triglycerides will be measured) to calculate HOMA scores and evaluate insulin sensitivity.

The following assessments will be added to the participant pathway at baseline/screening and 12 month visits:

- **Neurophysiology:** Participants will undergo a clinical neuropathy screen using the Utah Early Neuropathy Score, allodynia testing as described by LoPinto [47], and basic electrophysiology testing for the metabolic syndrome sub-study as described in 11.7.2 above. The neurophysiology testing will take around 20 minutes in total. These assessments will be reported on an additional CRF:

[**] Framingham Risk Score is an algorithm using age, gender, cholesterol levels, blood pressure and smoking status to evaluate an individual's 10 year cardiovascular risk score.

- Neurophysiology Form: to be completed by the clinicians who will be carrying out the relevant standard clinical practice assessments or from their patient notes.

- **Skin biopsy sample:** Participants will then undergo a 3mm punch skin biopsy at the lower leg with appropriate sterile technique and under local anaesthetic. This will take around 15 minutes in total.

11.6. Magnetic resonance imaging in IIH sub-study

The overall aim of this sub-study is to characterise magnetic resonance imaging features in IIH and to evaluate the potential role of these features as imaging biomarkers for diagnosis and for monitoring disease progression.

We will look particularly at the role of brain compliance in IIH using a non-invasive MRI-based technique called MR-ICP. Compliance is a measure of the ability of the brain to respond to changes in fluid distribution. A compliant brain is able to tolerate changes in fluid balance without suffering from major elevation in ICP, while a non-compliant brain loses this capability. In IIH, we note raised ICP in the absence of hydrocephalus (dilated ventricles), and thus it is likely that brain biomechanics in general, and more specifically the stiffness of brain tissue, is an important mediating factor in disease development.

A novel MRI-based technique capable of noninvasively assessing intracranial compliance and potentially measuring ICP, (termed “MR-ICP”) has been developed and shows great promise in early studies [48-51]. MR-ICP now needs evaluation in the clinical setting.

Cerebral venous sinus compression is well documented in IIH [7] which may further exacerbate CSF drainage at the arachnoid granulation tissue [8]. Venous stenoses are a target for therapeutic stenting in some centres. Changes in the calibre of the venous sinuses are noted in up to 90% of IIH patients [52] and the presence of these stenoses as an imaging biomarker in IIH has been suggested, although the finding can occur in other conditions characterised by elevated ICP [52, 53]. Additionally, volumetric assessment of the optic nerve sheath in IIH has been shown to vary with ICP [54]. No studies have yet assessed the impact of weight loss on venous sinus stenoses in IIH.

Other imaging features characteristic of IIH include “empty sella”, optic nerve sheath distension, and posterior optic globe flattening, but these do not correlate with LP measures of ICP [54].

Objectives for magnetic resonance imaging sub-study

Primary objective:

- To evaluate MRI in patients with active IIH (at baseline) and then after 12 months of therapeutic weight loss (achieved through bariatric surgery or dietary weight loss programme).

Secondary objectives:

- To evaluate the relationship between MRI and ICP and papilloedema as measured by ocular coherence tomography.
- To evaluate magnetic resonance venography (MRV) imaging (cranial venous outflow obstruction index) [55] pre- and post- bariatric surgery/dietary weight loss to establish if venous stenoses are modified by weight loss and, using multivariate regression analysis, evaluate their relationship to ICP and visual function.

Method for imaging sub-study

We will use MRI (3 Tesla scanner) to measures brain stiffness or membrane compliance (e.g. ventricles) as well as volumetric changes in the optic nerve sheath, alterations in calibre of the venous sinuses using MR-ICP, diffusion tensor imaging and MRV. These sequences take about 30 minutes of scanning time.

Participants will be imaged at baseline and at 12 months using these techniques (obese controls at baseline only). Additionally, in order to characterise the immediate effects of reduction in ICP, 5 of these

participants will be imaged additionally, after their baseline LP, enabling measurement pre- and post- LP on the same day. We will sequentially ask participants until 5 have agreed to this. This will be done only at baseline.

11.7. Cognitive function sub-study

Patients with IIH frequently describe memory impairment and a recent retrospective study of 10 IIH patients has provided evidence that cognitive deficits likely exist in patients with IIH [56]. However there has been very little formal testing to characterise cognitive deficits in IIH [57, 58]. Additionally, there has been no evaluation of the extent to which the different features, symptoms and co-morbidities of IIH (headache, depression, raised ICP, obesity, sleep apnoea) contribute towards cognitive dysfunction.

Migrainous headaches, a phenotype of headache frequently experienced by IIH patients [59], have been shown to impair cognition compared to headache free periods [60]. Cognitive impairment is a well-recognised feature in conditions characterised by chronically raised ICP such as hydrocephalus [61, 62] and normal pressure hydrocephalus [63]. Additionally, depression, a frequent co-morbid condition in IIH, has been linked to deficits in memory and attention [64, 65]. Obesity and OSA are also linked to impaired cognition [66, 67]. It is intriguing to speculate that dysfunction of the cortisol generating enzyme 11β-hydroxysteroid dehydrogenase, a characteristic feature of obesity and IIH, could contribute to cognitive deficits in IIH [68, 69].

Cognitive screening of trial participants will be conducted. These tests will all involve looking at different images on a screen and making a response to evaluate cognitive function and are described below.

Objectives for cognitive evaluation sub-study
Primary objective:

- To evaluate cognitive function in patients with active IIH (at baseline) and then after 12 months of therapeutic weight loss (achieved through bariatric surgery or dietary weight loss programme)

Secondary objectives:

- To evaluate the relationship between cognitive function and headache disability scores / index, depression scores, sleep apnoea scores, ICP and body mass index.

Method for cognitive sub-study
Participants will undergo a battery of cognitive tests at baseline and 12 months (obese controls at baseline only). Headache severity at the time of the test will be rated by the participant on a scale of 0-10.

The following assessments will be added to the participant pathway at baseline/screening and 12 month visits:

- **Cognitive testing:** Cognitive tests will be conducted as described below by a research nurse. Tests will take approximately one hour using a computer. The participant will be asked to grade their headache from 0-10 before undergoing the test (and again before undergoing the single repeated Sustained Attention to Response Task after LP):

1. Verbal Short-Term Memory: Word Span (15 minutes)
Participants recall sequences of one- and two-syllable nouns that are presented in lowercase for 1 second each, with a 500 ms blank screen between each word. Participants name each word aloud as it appears. Set sizes range from two to seven words, with each set size presented three times (18 sets total). No word appears more than once during the task.

2. Verbal Working Memory: Operation Span (15 minutes)
Participants recall words against a background arithmetic task. Each display includes a mathematical problem followed by a to-be-remembered word (e.g., "Is (7 x 2) - 1 = 13?" "Car"). The arithmetic operation begins with a parenthetical multiplication or division problem (each equally represented) followed by a

number to add to or subtract from the product or dividend (each equally represented). As soon as the equation appears, participants read the equation aloud. They say, "yes" or "no" out loud to indicate whether the equation is correct or incorrect (correct and incorrect equations are shown approximately half the time each).

3. The Attention Network Test-Interactions (20 minutes)

This is used to measure the alerting, orienting, and executive components of attention, and the interactions among these networks. A fixation cross is presented in the centre of the screen, and remains on the screen. An auditory signal is presented for 50 ms on half of trials, between 400 and 1600 ms after each trial is started. At 450 ms after the onset of the auditory signal, a visual cue is presented either above or below fixation on two-thirds of trials, lasting 100 ms. The target is the centre arrow in a set of five arrows that appears either above or below the fixation. The task is to report the direction of the centre arrow; the arrows flanking the centre arrow are either congruent in direction with the centre arrow, or incongruent in direction.

4. Sustained Attention to Response Task (SART) (15 minutes)

SARTs are vigilance tasks that require that participants sustain their attention so as to minimise distractibility. These tasks require that participants identify very infrequent targets with a key press response, or to withhold key press responses to very infrequent targets.

The SART test will be carried out twice per participant assessment visit: both before and after the LP. This will be to assess the effect of the LP and subsequent reduction in ICP on the result. Several studies show that repeated tests do not show the effects of practice [70-72].

5. Pattern-glare Test (5 minutes)

Participants are shown a series of single images containing black and white stripes and are asked to grade their response to how uncomfortable the image is to look at.

6. An IQ test (Raven's Standard Progressive Matrices – 15-30 minutes) will be performed at the baseline visit (or first available time point if not possible at baseline).

7. An air pollution screening tool (Lifetime Exposure to Air Pollution Scale – 15 minutes) will be completed at the baseline visit (or first available time point if not possible at baseline). This will be administered by the local research team and not completed by the participant.

8. The matched obese control group (see section 12.1 below) will also undergo the **National Institutes of Health ToolBox Cognitive Battery**. This is a collection of cognitive instruments which test an array of cognitive attributes including episodic memory, executive function, processing speed, multi-tasking and planning. They will be delivered by an iPad application supervised by a member of the trial team and last upto 45 minutes. There are no significant risks to the test and burden is limited to time only. This is a validated collection of tests [73, 74].

11.8. Exploratory samples

Additional urine, blood, skin and fat samples will be taken from participants taking part in the exploratory sub-studies. Additional serum samples will be taken at baseline, 12, 24 and 60 months. 24 hour urine collections will also be taken at baseline and at 12 months. A 24 hour urine bottle will be provided to the participant at pre-screening; at 12 months a research nurse at the centre will post the 24 hour urine bottle to the participant prior to their assessment visit. Skin and fat samples will be taken at the time of surgery from participants undergoing surgery at BHH.

The use and storage of these additional samples is detailed overleaf.

Urine

A 24 hours urine collection will be collected from the participant.

Venesection

The participant will undergo additional fasted blood sampling for exploratory analysis:

- Biomarker analysis including nitrotyrosine - 1 red tube of blood
- Polymorphism studies - 1 purple tubes of blood.
- Peripheral Blood Mononuclear Cells (PBMC) from whole blood - 2 purple tubes of blood.

The total quantities of blood taken from participants giving these additional samples are summarised in table 8, 9 and 10 below:

Table 8: Blood samples and tubes (baseline, 12, 24 and 60 months)

Fasting metabolic evaluation	PCOS	Exploratory analysis	Polymorphism studies	Pre and Post meal samples	PBMC (baseline only)
1 purple tube, 1 grey tube, 1 yellow tube	1 yellow tube, 1 red tube	1 yellow tube, 1 red tube	1 purple tube	6 purple GLP-1 tubes	2 purple tubes

Table 9: Blood samples and quantities (baseline and 20 obese controls)

Yellow tubes (4mls)	Grey tubes (2mls)	Purple tubes (4mls)	Purple GLP-1 tubes (2mls)	Red tubes (6mls)	Volume collected in mls
3	1	4	6	2	54

Table 10: Blood samples and quantities (12, 24 and 60 months)

Yellow tubes (4mls)	Grey tubes (2mls)	Purple tubes (4mls)	Purple GLP-1 tubes (2mls)	Red tubes (6mls)	Volume collected in mls
3	1	3	6	2	50

Punch skin biopsy

A 3mm sample will be taken as described in 11.5 (baseline and 12 months) for analysis at the UHB pathology department.

Bariatric surgery samples

Samples will be taken under general anaesthetic at the time of surgery.

Skin: A 10mm ellipse of skin will be taken from the laparoscopic port site.
Fat: A 10mm cubed sample of both subcutaneous and omental fat will be collected.

11.9. Processing and storage of additional samples

The processing of the additional samples taken at the lead site is detailed below:

Urine

The 24 hour urine collection will be measured for total volume and this will be recorded on the assessment CRF. The sample will then be processed according to the laboratory manual and the aliquots stored at -80°C, initially at the site before transfer to UoB for analysis of total corticosteroid metabolite levels, [THF+alloTHF]/THE ratio, cortols/cortolones and total androgen metabolites.

Blood

- Biomarkers: 1 red tube will be processed according to the laboratory manual and the aliquots stored at -80°C, initially at the site before transfer to UoB for storage and biomarker analysis.
- PBMCs: 2 purple tubes will be processed according to the laboratory manual and the aliquots stored at -80°C, initially at the site before transfer to UoB for storage. PBMC from this whole blood will be used to generate induced pluripotent stem cells for future study of CSF regulating tissues and IIH.
- Polymorphism: 1 purple tube will be processed according to the laboratory manual and the aliquots stored at -80°C, initially at the site before transfer to UoB for storage and polymorphism analysis.

Skin samples

Punch biopsies of the skin from the shin will be transported to the UHB pathology department for analysis of the intraepidermal nerve fibre density. Skin biopsies may be used to generate induced pluripotent stem cells for future study of CSF regulating tissues and IIH.

Bariatric surgery samples

Samples will be transported in RNALater immediately from BHH, and brought to the UoB and stored at the -80 degrees Celsius freezer at the UoB for batched analysis.

On these skin and fat samples, molecular biology techniques (e.g. polymerase chain reaction, western blotting, immunohistochemistry, microarray, enzyme activity assays and cell culture techniques) will be used to explore neuropeptides, growth factors, markers of hypoxia as well as the hormonal, vitamin and inflammatory pathways involved in IIH with the aim of improving our understanding of the pathogenesis of IIH.

12. EXPLORATORY SUB-STUDIES: ADDITIONAL RECRUITMENT GROUPS

Additional groups of participants will be recruited to facilitate the sub-studies described in Section 11.

12.1. Matched obese control group sub-study

IIH is strikingly associated with obesity, 87.8 – 94% of patients with IIH being obese [75-77]. The incidence of IIH increases to between 19.3 and 21 per 100,000 in the obese population compared with 0.9 to 2.2 per 100,000 in the general population [78-80].

The mechanism by which obesity causes IIH is debatable. OSA, a condition associated with obesity, leads to nocturnal hypercapnia, right heart failure and surges in intra-thoracic pressure which can elevate ICP particularly in the morning compared to the evening [81]. It has also been suggested that pressure effects of centrally distributed adiposity elevate intra-abdominal pressure which subsequently elevates intra-thoracic pressure, cerebral venous pressure, and finally ICP [82]. This theory does not explain why despite ubiquitous elevation of intra-abdominal pressure in obese patients [83, 84], only a small proportion of patients develop IIH.

Raised ICP is characteristic of IIH. However, the influence of obesity on ICP is not well established and the normal ICP in obese individuals is contentious. In the only study in this area, a weak, non-significant relationship between BMI and LP opening pressure was noted (although only 44 patients with a BMI >30kg/m^2 were evaluated) [84]. We aim to conduct LPs in a cohort of 20 obese patients with a BMI >35kg/m^2 who do not have IIH and consequently make this vital and novel observation of 'normal' ICP in morbidly obese individuals. This result will have profound implications to help establish the normal range of ICP in this patient population. Results will provide vital and much needed evidence to facilitate the

diagnosing of conditions of raised ICP, such as IIH, in the obese. This is particularly important in cases of suspect IIH without papilloedema where there are no other indicators of raised ICP besides headache and diagnosis is very uncertain.

Finally, throughout this trial we are characterising the co-morbidities of IIH which extend beyond visual loss. We propose that there is significant metabolic comorbidity (impaired insulin sensitivity, Framingham Cardiovascular disease risk score, peripheral nerve function and 11β hydroxysteroid dehydrogenase function). Further, we predict that cognitive function may be impaired in patients with IIH. It has not been established, however, to what extent obesity in IIH influences these potentially associated co-morbidities. Through this sub-study we will be able to explore the influence of obesity on IIH.

We will assess at least 20 obese participants without IIH. The cohort will be matched for age, gender and BMI. These participants will undergo the same baseline visit as main trial participants with all exploratory sub-studies as described in Section 11 and then exit the study. They will not complete any health economics questionnaires. A subset of control patients will be recruited to undergo only visual assessments and medical examination/history; this subset will have a separate PIS describing the assessments they will undergo (PIS Control "Light"). The recruitment target will be 20 participants undergoing the full baseline assessment day.

Objectives for matched obese control group sub-study

Primary objectives:

- To evaluate the baseline difference in ICP between IIH patients and a matched obese control cohort.

Secondary objectives:

- To evaluate the baseline difference in visual function, headache disability, sleep apnoea, cognitive testing and features of the metabolic syndrome (including peripheral neurophysiology and nerve fibre density) between IIH patients and a matched obese control cohort.
- To evaluate the baseline difference in biomarker analysis between IIH patients and a matched obese control cohort

12.1.1 Eligibility for matched obese control group sub-study

Inclusion criteria:

1. Female.
2. BMI >35kg/m^2.
3. Able to give informed consent.
4. Aged between 18 and 55 years.

Exclusion criteria:

1. Pregnant.
2. Inability to give informed consent e.g. due to cognitive impairment.
3. Diagnosis of IIH.

Trial participants will be matched to a closest matching control after recruitment has ended and interim analyses will be performed to monitor sub-study recruitment and inform remaining sub-study recruitment to ensure suitable matches are possible.

Recruitment to the matched obese control group sub-study

Potential participants to the matched obese control group sub-study will be identified and approached by research staff at primary care PIC sites, as well as in secondary care. Main trial participants will also be asked if they have friends or family who may meet the above eligibility criteria and be interested in taking part. Appropriate advertising to potential participants will be introduced. In some cases initial discussion

with potential participants may take place by telephone. The clinician or research nurse will introduce the sub-study to potential participants, and will provide the potential participant with sub-study specific PIS and consent forms so that they can find out more about the sub-study before deciding whether or not to participate.

They will be given time to consider participation in the sub-study and if they wish to take part they will be offered an appointment at UHB for an assessment visit. If the participant gives written informed consent before this full visit then they may be given a headache diary, 24 hour urine collection bottle and sleep monitor to take home and bring back for this visit. If necessary, the headache diary may be completed retrospectively on the day of the visit.

Participants in the matched obese control group sub-study will undergo the same screening/baseline assessment day as main trial participants at UHB, and then leave the trial. A subset of control participants will undergo only visual assessments and medical examination/history.

If any abnormalities are found which require follow up participants will be contacted by phone and if necessary invited to return to discuss the findings. The researcher will use their clinical judgement to decide if the participant needs to be referred to an appropriate service.

12.2. MRI Test Run sub-study

To validate the novel magnetic resonance scan sequences used in the MRI sub-study, at least 5 healthy individuals will be scanned twice at least 2 weeks apart. The anonymised scans will be sent to the MRI collaborator for evaluation to check the scanning procedures are suitable for use in the MRI sub-study.

Objectives for MRI Test Run sub-study

Primary objective:

- To validate the MRI test sequences being used in the MRI sub-study.

12.2.1 Eligibility for MRI Test Run sub-study

Inclusion criteria:

1. Age between 18 and 65 years.
2. Able to give informed consent.

Exclusion criteria:

1. Aged under 18 or over 65 years.
2. Inability to give informed consent e.g. due to cognitive impairment.
3. Pregnant.
4. Pacemaker, metal implants, prosthetics, pins, plates, or metal fragments in body (including in the eye but not including dental fillings).

Recruitment to the MRI Test Run sub-study

The MRI test group participants will be recruited through the use of posters and fliers at UHB and UoB. Potential participants will contact a named member of the research team through contact details available on these posters and fliers. The researcher will introduce the MRI Test Run sub-study to them, and will provide them with sub-study specific PIS and consent forms so that potential participants can find out more about the sub-study before deciding whether or not to take part.

They will then have the opportunity to discuss any questions they may have before an appointment for the test scan at UHB is made. At this appointment, and before any trial scans are run, they will have the opportunity to ask any questions they may have before being asked to give written informed consent.

Healthy controls for the MRI test run group will undergo a baseline MRI scan and a second scan at least 2 weeks later, and then leave the trial.

If any abnormalities are found which require follow up participants will be contacted by phone and if necessary invited to return to discuss the findings. The researcher will use their clinical judgement to decide if the participant needs to be referred to an appropriate service.

12.3. Control Fat and Skin Sample sub-study

To gain the full benefit from the fat and skin samples taken from the main trial participants referred to bariatric surgery, obese patients who have not been diagnosed with IIH will be approached at BHH for similar quantities of subcutaneous and omental fat samples as well as skin samples as detailed in 11.8. These participants' weight, age, height and sex will also be recorded as well as clinically relevant co-morbidities. The purpose of these samples will be to optimise the experiments before performing them on the main trial participants' samples.

Objectives for Control Fat and Skin Sample sub-study
Primary objective:

- To obtain subcutaneous and omental fat samples and skin samples to use as control samples.

12.3.1 Eligibility for Control Fat and Skin Sample sub-study

Inclusion criteria:

1. Age between 18 and 65 years.
2. Able to give informed consent.

Exclusion criteria:

1. Aged under 18 or over 65 years.
2. Inability to give informed consent e.g. due to cognitive impairment.
3. Diagnosis of IIH.

Recruitment to the Control Fat and Skin Sample sub-study

Participants in this sub-study will be recruited at the lead surgery site. Suitable potential participants will be approached by the research team at this site. The researcher will introduce the sub-study to them, and will provide them with sub-study specific PIS and consent form so that potential participants can find out more about the sub-study before deciding whether or not to take part.

They will then have the opportunity to discuss any questions before their scheduled procedure, and will be asked to give written informed consent.

13. DATA ACCESS AND QUALITY ASSURANCE

13.1. Monitoring and Audit

The investigators and institutions will permit trial-related monitoring, audits and REC review, providing direct access to source data/documents. Trial participants are informed of this during the informed consent discussion and will consent to provide access to their medical notes. Monitoring of this trial will be to ensure compliance with GCP.

A risk proportionate approach to the initiation, management and monitoring of the trial will be adopted and outlined in the trial-specific risk assessment.

13.2. Site Set-up and Initiation

All PIs will be asked to sign a Site Signature and Delegation log, the Protocol PI signature page, and to supply a current CV and GCP certificate to BCTU. All members of the site research team are required to sign the Site Signature and Delegation Log, which details which tasks have been delegated to them by the PI.

Prior to commencing recruitment, each recruiting site will undergo a process of initiation, either a meeting or a teleconference, which key members of the site research team are required to attend, covering aspects of the trial design, protocol procedures, adverse event reporting, collection and reporting of data and record keeping. Sites will be provided with an Investigator Site File containing essential documentation, instructions, and other documentation required for the conduct of the trial. The trial office must be informed immediately of any change in the site research team.

13.3. Central Monitoring

Trials staff will be in regular contact with the site research team to check on progress and address any queries that they may have. Trials staff will check incoming ICFs and CRFs for compliance with the protocol, data consistency, missing data and timing. Sites will be sent DCFs requesting missing data or clarification of inconsistencies or discrepancies.

On-site monitoring visits may be triggered, for example by poor CRF return, poor data quality, low SAE reporting rates, excessive number of participant withdrawals or deviations. If a monitoring visit is required the Trials team will contact the site to arrange a date for the proposed visit and will provide the site with written confirmation. Investigators will allow the trial staff access to source documents as requested.

13.4. Notification of Serious Breaches

The sponsor is responsible for notifying the REC of any serious breach of the conditions and principles of GCP in connection with that trial or the protocol relating to that trial. Sites are therefore requested to notify the trial office of any suspected trial-related serious breach of GCP and/or the trial protocol. Where the trial office is investigating whether or not a serious breach has occurred sites are also requested to cooperate with the trial office in providing sufficient information to report the breach to the REC where required and in undertaking any corrective and/or preventive action.

Sites may be suspended from further recruitment in the event of serious and persistent non-compliance with the protocol and/or GCP, and/or poor recruitment. Any major problems identified during monitoring may be reported to the TMG, TSC, and REC. This includes reporting serious breaches of GCP and/or the trial protocol to the REC. A copy is sent to the University of Birmingham Clinical Research Compliance Team at the time of reporting to the REC.

13.5. Independent Trial Steering Committee

The TSC provides independent supervision for the trial, providing advice to the Chief and Co-Investigators and the Sponsor on all aspects of the trial and affording protection for participants by ensuring the trial is conducted according to the principles of GCP in Clinical Trials.

If the Chief and Co-Investigators are unable to resolve any concern satisfactorily, PIs and all others associated with the trial may write through the Trial Office to the chairman of the TSC, drawing attention to any concerns they may have about the possibility of particular side-effects, or of particular categories of participant requiring special study, or about any other matters thought relevant.

The TSC will comprise an independent chairperson, one other independent specialist, one independent statistician, one independent patient and public involvement representative, and the CI. This group will meet at the beginning of the trial and thereafter up to six monthly depending on progress.

13.6. Data Monitoring Committee: determining when clear answers have emerged

If one treatment arm is more effective with respect to the primary endpoints than the other, then this may become apparent before the target recruitment has been reached. Alternatively, new evidence might emerge from other sources that one of the treatment arms is definitely effective. To protect against this, during the period of recruitment to the trial, interim analyses of major endpoints will be supplied, in strict confidence, to an independent DMC along with updates on results of other related studies, and any other

analyses that the DMC may request. The DMC will advise the chair of the TSC if, in their view, either of the randomised comparisons in the trial have provided both (a) "proof beyond reasonable doubt"[††] that for all, or for some, types of participant one particular treatment is definitely indicated or definitely contraindicated in terms of a net difference in the major endpoints, and (b) evidence that might reasonably be expected to influence the patient management of many clinicians who are already aware of the other main trial results. The TSC can then decide whether to close or modify any part of the trial. Unless this happens, however, the TMG, TSC, the investigators and all of the central administrative staff (except the statisticians who supply the confidential analyses) will remain unaware of the interim results.

14. ORGANISATION AND RESPONSIBILITIES

To ensure the smooth running of the trial and to minimise the overall procedural workload, it is proposed that each participating centre should designate individuals who will be chiefly responsible for local co-ordination of clinical and administrative aspects of the trial.

All Investigators are responsible for ensuring that any research undertaken follows the agreed protocol, for helping care professionals to ensure that participants receive appropriate care while involved in research, for protecting the integrity and confidentiality of clinical and other records and data generated by the research, and for reporting any failures in these respects, adverse reactions and other events or suspected misconduct through the appropriate systems.

14.1. Principal Investigator at each centre

The responsibilities of the local Principal Investigator are for the conduct of research at their centre and to ensure that all medical and nursing staff involved in the care of the participant are well informed about the trial and trained in trial procedures, including obtaining informed consent. The local Principal Investigator should liaise with the Trial Coordinator on logistic and administrative matters connected with the trial.

14.2. Nursing Co-ordinator at each centre

Each participating centre should designate one nurse as local Nursing Coordinator. This person will be responsible for ensuring that all eligible patients are considered for the trial, that potential participants are provided with PIS, and have an opportunity to discuss the trial if required. The nurse may be responsible for collecting the baseline participant data and for administering the follow-up evaluations.

14.3. The Neuroscience Trials Office

The trial office at UoB is responsible for providing all trial materials, including the trial folders containing printed materials. These will be supplied to each collaborating centre, after relevant ethics committee and R&D approval has been obtained. Additional supplies of any printed material can be obtained on request. The trial office is responsible for collection and checking of data (including reports of SAEs thought to be due to trial treatment). The trial office will help resolve any local problems that may be encountered in trial participation.

14.4. Research Governance

The conduct of the trial will be in accordance with the Medical Research Council Guidelines for Good Clinical Practice 1998 and the Research Governance Framework for Health and Social Care. Participants/carers will be involved in the ethics process, ensuring that all PIS and consent forms are fit for purpose. The trial will adhere to the principles of GCP and the Declaration of Helsinki (2008 / 1996).

---

†† Appropriate criteria of proof beyond reasonable doubt cannot be specified precisely, but a difference of at least $p<0.001$ (similar to Haybittle-Peto boundary) in an interim analysis of a major endpoint may be needed to justify halting, or modifying, the study prematurely. If this criterion were to be adopted, it would have the practical advantage that the exact number of interim analyses would be of little importance, so no fixed schedule is proposed.

All centres will be required to sign an Investigator's Agreement, detailing their commitment to accrual, compliance, GCP, confidentiality and publication. Deviations from the agreement will be monitored and the TSC will decide whether any action needs to be taken, e.g. withdrawal of funding, suspension of centre.

The trial office will ensure researchers not employed by an NHS organisation hold an NHS honorary contract for the relevant organisation.

14.5. Ethical and Trust Management

The Trial has a favourable ethical opinion from West Midlands – The Black Country Research Ethics Committee (REC), determining that the trial design respects the rights, safety and wellbeing of the participants. The Trust Research and Development Office need to assess the "locality issues" relating to their population, the investigators, the facilities and resources. The trial office is able to help the local Principal Investigator in the process of the site specific assessment and NHS permission by completing as much of the standard IRAS form as possible. The local Principal Investigator will be responsible for liaison with the Trust and/or Local Research Network with respect to locality issues and obtaining the necessary signatures at their Trust.

As soon as REC and Trust/Network approval has been obtained, the trial office will send a folder containing all trial materials to the local Principal Investigator. Entry of participants into the trial can then begin.

14.6. Funding and Cost implications

The research costs of the trial are funded by a clinical fellowship from the NIHR awarded to the CI. A subvention for the costs of surgery and Weight Watchers will be payable from this. Participant travel (up to £120 total) will be paid from this, as will £125 offered as a compensation for loss of time and earnings at the 12, 24 and 60 month visits. Participants in the matched obese control group will be offered a compensation for loss of time and earnings of £200 for a full baseline assessment day. Their reasonable travel expenses for this visit will also be refunded from this clinical fellowship. MRI Test Run and Sample Control participants will not be offered any payment or travel expenses. Further help with participant travel expenses has been kindly donated by the patient charity IIH UK.

The trial has been adopted onto the NIHR portfolio and so the 'NHS service support' costs for this trial will be met by CLRN.

Additional costs associated with the trial, e.g. gaining consent, baseline tests, for nurses to explain the questionnaires to participants, etc., are estimated in the standard IRAS form. These costs should be met by accessing the Trust/Network's support budget.

14.7. Indemnity

There are no special arrangements for compensation for non-negligent harm suffered by participants as a result of participating in the trial. The trial is not an industry-sponsored trial and so ABPI/ABHI guidelines on indemnity do not apply. The normal NHS indemnity liability arrangements for research detailed in HSG96(48) will operate in this case.

However, it should be stressed that in terms of negligent liability, NHS Trust hospitals have a duty of care to a patient being treated within their hospital, whether or not that patient is participating in a clinical trial. Apart from defective products, legal liability does not arise where there is non-negligent harm. NHS Trusts may not offer advance indemnities or take out commercial insurance for non-negligent harm.

14.8. Publication

Results of this trial will be submitted for publication in a peer-reviewed journal. The manuscript will be prepared by the IIH:WT trial team and authors will include the CI, collaborators, co-investigators, and BCTU

staff (as long as all listed had reasonable contributions). Results will be disseminated to participants by using a participant newsletter, through patient charities, and on the trial website.

15. PROJECT TIMETABLE AND MILESTONES

Six months have been set aside to recruit and train staff, to identify patients/carers for the involvement group, to gain NRES, SSA and R & D department approvals and to set up trial procedures. With 45 months for recruitment, 60 months to follow the last participant, and 6 months for data analysis, the trial will take 117 months to complete as shown on table 11 overleaf. As the primary endpoint is at 12 months, the trial will take 69 months to reach the publication of its main results.

Table 11: IIH:WT timetable

Time	Action
August 2013	Trial officially commences
November 2013 onwards	Applications for SSA and R&D approval submitted
February 2014	Recruitment commences
October 2017	Main trial recruitment completed
October 2018	Control participant recruitment completed<br>Last participant reaches primary endpoint<br>Data analysis commences<br>Report written
April 2019	Paper submitted for publication
October 2022	Last participant completes 60 month follow up<br>Data analysis commences
April 2023	Long-term follow up paper submitted for publication

16. APPENDIX A: FRISÉN GRADING

Modified Frisén Scale for Grading Papilledema

Grade 1 - C-Shaped halo with a temporal gap

Grade 2 - The halo becomes circumferential

Grade 3 - Loss of major vessels as they *leave* the disc

Grade 4 - Loss of major vessels *on the disc*

Grade 5 - Criteria of Grade IV + partial or total obscuration of *all* vessels on the disc

From CJ Scott et al., 2010 [1]

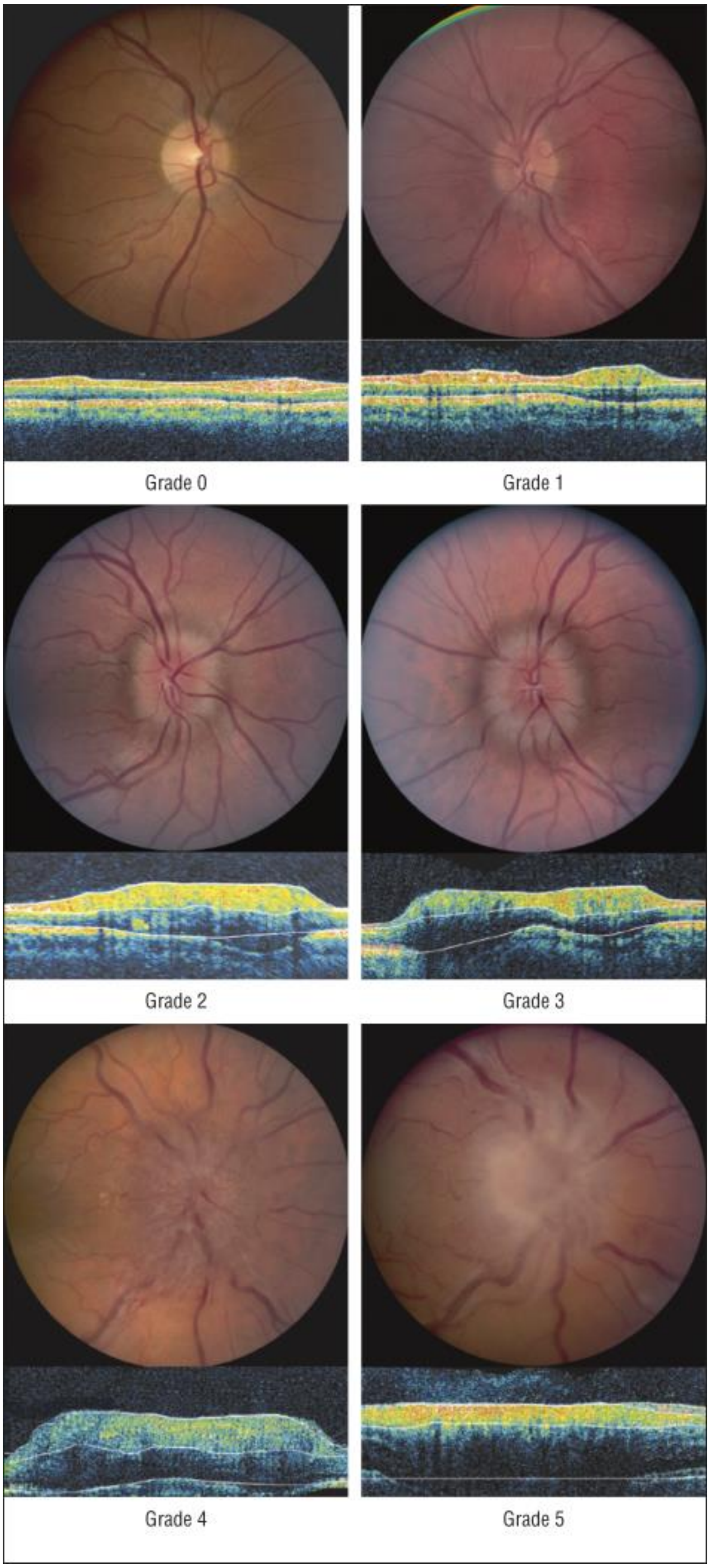

17. REFERENCES

1. Scott, C.J., et al., Diagnosis and grading of papilledema in patients with raised intracranial pressure using optical coherence tomography vs clinical expert assessment using a clinical staging scale. Arch. Ophthalmol, 2010. **128**(6): p. 705-711.

2. Organization, W.H. *Global Database on Body Mass Index*. 2013 2013 [cited 2013 9/19/13 AD]; Available from: http://apps.who.int/bmi/index.jsp.

3. Lueck, C. and G. McIlwaine, *Interventions for idiopathic intracranial hypertension.* Cochrane Database Syst Rev, 2005(3): p. CD003434.

4. Ball, A.K., et al., A randomised controlled trial of treatment for idiopathic intracranial hypertension. J Neurol, 2011. **258**(5): p. 874-81.

5. Celebisoy, N., et al., Treatment of idiopathic intracranial hypertension: topiramate vs acetazolamide, an open-label study. Acta Neurol Scand, 2007. **116**(5): p. 322-327.

6. Curry, W.T., W.E. Butler, and F.G. Barker, Rapidly rising incidence of cerebrospinal fluid shunting procedures for idiopathic intracranial hypertension in the United States, 1988-2002. Neurosurgery, 2005. **57**(1): p. 97-107.

7. Sinclair, A.J., et al., Is cerebrospinal fluid shunting in idiopathic intracranial hypertension worthwhile? A 10-year review. Cephalalgia, 2011. **31**(16): p. 1627-1633.

8. Abubaker, K., et al., Idiopathic intracranial hypertension: lumboperitoneal shunts versus ventriculoperitoneal shunts--case series and literature review. Br J Neurosurg, 2011. **25**(1): p. 94-9.

9. Newborg, B., *Pseudotumor Cerebri Treated by Rice-Reduction Diet.* Arch Intern Med, 1974. **133**(5): p. 802-807.

10. Sinclair, A.J., et al., Low energy diet and intracranial pressure in women with idiopathic intracranial hypertension: prospective cohort study. BMJ, 2010. **341**: p. c2701.

11. Fridley, J., et al., Bariatric surgery for the treatment of idiopathic intracranial hypertension. J Neurosurg, 2011. **114**(1): p. 34-39.

12. Colquitt, J.L., et al., *Surgery for obesity.* Cochrane Database Syst Rev, 2009(2): p. CD003641.

13. Camastra, S., et al., Early and longer term effects of gastric bypass surgery on tissue-specific insulin sensitivity and beta cell function in morbidly obese patients with and without type 2 diabetes. Diabetologia, 2011. **54**(8): p. 2093-2102.

14. Dixon, J.B., et al., Adjustable gastric banding and conventional therapy for type 2 diabetes - A randomized controlled trial. JAMA, 2008. **299**(3): p. 316-323.

15. Singhal, R., et al., Medium-term outcomes of patients with insulin-dependent diabetes after laparoscopic adjustable gastric banding. Surg Obes Relat Dis, 2013. **9**(1): p. 42-47.

16. Singhal, R., et al., Metabolic Outcomes of Obese Diabetic Patients Following Laparoscopic Adjustable Gastric Banding. Obes Surg, 2008. **18**(11): p. 1400-1405.

17. Schauer, D.P., et al., Decision Modeling to Estimate the Impact of Gastric Bypass Surgery on Life Expectancy for the Treatment of Morbid Obesity. Arch Surg, 2010. **145**(1): p. 57-62.

18. Picot, J., et al., The clinical effectiveness and cost-effectiveness of bariatric (weight loss) surgery for obesity: a systematic review and economic evaluation. Health Technol Assess, 2009. **13**(41): p. 1-+.

19. Hutter, M.M., et al., First report from the American College of Surgeons Bariatric Surgery Center Network: laparoscopic sleeve gastrectomy has morbidity and effectiveness positioned between the band and the bypass. Ann Surg, 2011. **254**(3): p. 410-420.

20. Longitudinal Assessment of Bariatric Surgery, C., et al., *Perioperative safety in the longitudinal assessment of bariatric surgery.* N Engl J Med, 2009. **361**(5): p. 445-54.

21. Excellence, N.I.f.H.a.C. *Obesity: the prevention, identification, assessment and management of overweight and obesity in adults and children; CG43*. 2006 2006 [cited 2013 9/18/2013]; Available from: http://www.nice.org.uk/CG43.

22. Ko, M., et al., Weight gain and recurrence in idiopathic intracranial hypertension A case-control study. Neurology, 2011. **76**(18): p. 1564-1567.

23. Middleton, K.M., S.M. Patidar, and M.G. Perri, The impact of extended care on the long-term maintenance of weight loss: a systematic review and meta-analysis. Obes Rev, 2012. **13**(6): p. 509-17.

24. Astrup, A., et al., Effects of liraglutide in the treatment of obesity: a randomised, double-blind, placebo-controlled study. Lancet, 2009. **374**(9701): p. 1606-16.

25. Li, Z.P., et al., *Meta-analysis: Pharmacologic treatment of obesity.* Ann Intern Med, 2005. **142**(7): p. 532-546.
26. Chapman, A.E., et al., Laparoscopic adjustable gastric banding in the treatment of obesity: A systematic literature review. Surgery, 2004. **135**(3): p. 326-351.
27. O'Brien, P.E., et al., Systematic review of medium-term weight loss after bariatric operations. Obes Surg, 2006. **16**(8): p. 1032-1040.
28. Jolly, K., et al., Comparison of range of commercial or primary care led weight reduction programmes with minimal intervention control for weight loss in obesity: Lighten Up randomised controlled trial. BMJ, 2011. **343**: p. d6500.
29. Heshka, S., et al., Weight loss with self-help compared with a structured commercial program - A randomized trial. JAMA, 2003. **289**(14): p. 1792-1798.
30. Jebb, S.A., et al., Primary care referral to a commercial provider for weight loss treatment versus standard care: a randomised controlled trial. Lancet, 2011. **378**(9801): p. 1485-1492.
31. Truby, H., et al., Randomised controlled trial of four commercial weight loss programmes in the UK: initial findings from the BBC "diet trials". BMJ, 2006. **332**(7553): p. 1309-1311.
32. Friedman, D.I. and D.M. Jacobson, *Diagnostic criteria for idiopathic intracranial hypertension.* Neurology, 2002. **59**(10): p. 1492-5.
33. Sinclair, A.J., et al., Rating papilloedema: an evaluation of the Frisen classification in idiopathic intracranial hypertension. J Neurol Sci, 2012. **259**(7): p. 1406-1412.
34. Chung, F., et al., High STOP-Bang score indicates a high probability of obstructive sleep apnoea. Br. J. Anaesth, 2012. **108**(5): p. 768-775.
35. Chung, F., et al., STOP questionnaire: a tool to screen patients for obstructive sleep apnea. Anesthesiology, 2008. **108**(5): p. 812-821.
36. Asensio Sanchez, V.M., J. Merino Angulo, and A. Narros Gimenez, *Apnea syndrome: risk factors for pseudotumor cerebri.* An Med Interna, 2004. **21**(10): p. 520-520.
37. Shiba, T., et al., Nocturnal Intermittent Serious Hypoxia and Reoxygenation in Proliferative Diabetic Retinopathy Cases. Am J Ophthalmol, 2010. **149**(6): p. 959-963.
38. Mojon, D.S., et al., Association between sleep apnea syndrome and nonarteritic anterior ischemic optic neuropathy. Archives of Ophthalmology, 2002. **120**(5): p. 601-605.
39. Ruehland, W.R., et al., The New AASM Criteria for Scoring Hypopneas: Impact on the Apnea Hypopnea Index. Sleep, 2009. **32**(2): p. 150-157.
40. Smith, A.G., K. Rose, and J.R. Singleton, *Idiopathic neuropathy patients are at high risk for metabolic syndrome.* J Neurol Sci, 2008. **273**(1-2): p. 25-28.
41. Hoffman-Snyder, C., et al., Value of the oral glucose tolerance test in the evaluation of chronic idiopathic axonal polyneuropathy. Arch Neurol, 2006. **63**(8): p. 1075-1079.
42. Hughes, R.A.C., et al., A controlled investigation of the cause of chronic idiopathic axonal polyneuropathy. Brain, 2004. **127**(Pt 8): p. 1723-1730.
43. Smith, A.G., et al., *Lifestyle intervention for pre-diabetic neuropathy.* Diabetes Care, 2006. **29**(6): p. 1294-1299.
44. Singleton, J.R., et al., The Utah Early Neuropathy Scale: a sensitive clinical scale for early sensory predominant neuropathy. J Peripher Nerv Syst, 2008. **13**(3): p. 218-227.
45. Rajabally, Y.A., S. Beri, and J. Bankart, Electrophysiological markers of large fibre sensory neuropathy: a study of sensory and motor conduction parameters. Eur J Neurol, 2009. **16**(9): p. 1053-1059.
46. Devigili, G., et al., The diagnostic criteria for small fibre neuropathy: from symptoms to neuropathology. Brain, 2008. **131**(Pt 7): p. 1912-1925.
47. LoPinto, C., W.B. Young, and A. Ashkenazi, *Comparison of dynamic (brush) and static (pressure) mechanical allodynia in migraine.* Cephalalgia, 2006. **26**(7): p. 852-856.
48. Alperin, N., et al., MR-intracranial pressure (ICP): A method to measure intracranial elastance and pressure noninvasively by means of MR imaging: Baboon and human study. Radiology, 2000. **217**(3): p. 877-885.
49. Alperin, N., et al., MRI evidence of impaired CSF homeostasis in obesity-associated idiopathic intracranial hypertension. AJNR Am J Neuroradiol, 2013. **34**(1): p. 29-34.

50. Alperin, N., A. Sivaramakrishnan, and T. Lichtor, Magnetic resonance imaging-based measurements of cerebrospinal fluid and blood flow as indicators of intracranial compliance in patients with Chiari malformation. Journal of Neurosurgery, 2005. **103**(1): p. 46-52.
51. Glick, R.P., et al., Early experience from the application of a noninvasive magnetic resonance imaging-based measurement of intracranial pressure in hydrocephalus (and discussion 1060-1061). Neurosurgery, 2006. **59**(5): p. 1052-1060.
52. Kelly, L.P., et al., Does bilateral transverse cerebral venous sinus stenosis exist in patients without increased intracranial pressure? Clinical Neurology and Neurosurgery, 2013. **115**(8): p. 1215-1219.
53. Ridha, M.A., et al., Magnetic Resonance Imaging Findings of Elevated Intracranial Pressure in Cerebral Venous Thrombosis Versus Idiopathic Intracranial Hypertension with Transverse Sinus Stenosis. Neuro-Ophthalmology, 2013. **37**(1): p. 1-6.
54. Hoffmann, J., et al., Morphometric and volumetric MRI changes in idiopathic intracranial hypertension. Cephalalgia, 2013. **33**(13): p. 1075-1084.
55. Rohr, A. and J. Bindeballe, The entire dural sinus tree is compressed in patients with idiopathic hypertension: a longitudinal, volumetric magnetic resonance imaging study. Neuroradiology, 2011.
56. Kharkar, S., et al., Cognitive Impairment in patients with Pseudotumor Cerebri Syndrome. Behav Neurol, 2011. **24**(2): p. 143-148.
57. Kaplan, C.P., M.E. Miner, and J.M. McGregor, *Pseudotumour cerebri: Risk for cognitive impairment?* Brain Inj, 1997. **11**(4): p. 293-303.
58. Sorensen, P.S., A.M. Thomsen, and F. Gjerris, *Persistent Disturbances of Cognitive Functions in Patients with Pseudotumor Cerebri.* Acta Neurol Scand, 1986. **73**(3): p. 264-268.
59. Friedman, D.I. and E.A. Rausch, Headache, diagnoses in patients with treated idiopathic intracranial hypertension. Neurology, 2002. **58**(10): p. 1551-1553.
60. Mulder, E.J.C.M., et al., *Interictal and postictal cognitive changes in migraine.* Cephalalgia, 1999. **19**(6): p. 557-565.
61. Lacy, M., et al., Intellectual functioning in children with early shunted posthemorrhagic hydrocephalus. Pediatr Neurosurg, 2008. **44**(5): p. 376-381.
62. Mataro, M., et al., Neuropsychological findings in congenital and acquired childhood hydrocephalus. Neuropsychol Rev, 2001. **11**(4): p. 169-178.
63. Kazui, H., et al., Predictors of the disappearance of triad symptoms in patients with idiopathic normal pressure hydrocephalus after shunt surgery. J Neurol Sci, 2013. **328**(1-2): p. 64-69.
64. Malhi, G.S., et al., Neuropsychological deficits and functional impairment in bipolar depression, hypomania and euthymia. Bipolar Disord, 2007. **9**(1-2): p. 114-125.
65. Martinez-Aran, A., et al., Cognitive function across manic or hypomanic, depressed, and euthymic states in bipolar disorder. Am J Psychiatry, 2004. **161**(2): p. 262-270.
66. Bressler, J., et al., Fat mass and obesity gene and cognitive decline The Atherosclerosis Risk in Communities Study. Neurology, 2013. **80**(1): p. 92-99.
67. Kielb, S.A., et al., Cognition in Obstructive Sleep Apnea-Hypopnea Syndrome (OSAS): Current Clinical Knowledge and the Impact of Treatment. Neuromolecular Med, 2012. **14**(3): p. 180-193.
68. Chapman, K., M. Holmes, and J. Seckl, 11 Beta-Hydroxysteroid Dehydrogenases: Intracellular Gate-Keepers of Tissue Glucocorticoid Action. Physiological Reviews, 2013. **93**(3): p. 1139-1206.
69. Sinclair, A.J., et al., Cerebrospinal Fluid Corticosteroid Levels and Cortisol Metabolism in Patients with Idiopathic Intracranial Hypertension: A Link between 11 beta-HSD1 and Intracranial Pressure Regulation? Journal of Clinical Endocrinology & Metabolism, 2010. **95**(12): p. 5348-5356.
70. Manly, T., et al., Coffee in the cornflakes: time-of-day as a modulator of executive response control. Neuropsychologia, 2002. **40**(1): p. 1-6.
71. Manly, T., et al., The absent mind: further investigations of sustained attention to response. Neuropsychologia, 1999. **37**(6): p. 661-670.
72. Robertson, I.H., et al., 'Oops!': Performance correlates of everyday attentional failures in traumatic brain injured and normal subjects. Neuropsychologia, 1997. **35**(6): p. 747-758.
73. Heaton, R.K., et al., Reliability and validity of composite scores from the NIH Toolbox Cognition Battery in adults. J Int Neuropsychol Soc, 2014. **20**(6): p. 588-98.

74. Weintraub, S., et al., *Cognition assessment using the NIH Toolbox.* Neurology, 2013. **80**(11 Suppl 3): p. S54-64.

75. Galvin, J.A. and G.P. Van Stavern, Clinical characterization of idiopathic intracranial hypertension at the Detroit Medical Center. J Neurol Sci, 2004. **223**(2): p. 157-160.

76. Giuseffi, V., et al., Symptoms and Disease Associations in Idiopathic Intracranial Hypertension (Pseudotumor Cerebri) - A Case-Control Study. Neurology, 1991. **41**(2): p. 239-244.

77. Wall, M. and D. George, Idiopathic Intracranial Hypertension - A Prospective-Study of 50 Patients. Brain, 1991. **114**: p. 155-180.

78. Durcan, F.J., J.J. Corbett, and M. Wall, *The Incidence of Pseudotumor Cerebri - Population Studies in Iowa and Louisiana.* Arch Neurol, 1988. **45**(8): p. 875-877.

79. Ireland, B., J.J. Corbett, and R.B. Wallace, The Search for Causes of Idiopathic Intracranial Hypertension - A Preliminary Case-Control Study. Arch Neurol, 1990. **47**(3): p. 315-320.

80. Radhakrishnan, K., et al., Epidemiology of Idiopathic Intracranial Hypertension - A Prospective and Case-Control Study. J Neurol Sci, 1993. **116**(1): p. 18-28.

81. Jennum, P. and S.E. Borgesen, *Intracranial-Pressure and Obstructive Sleep-Apnea.* Chest, 1989. **95**(2): p. 279-283.

82. Sugerman, H., et al., Effects of Surgically Induced Weight-Loss on Idiopathic Intracranial Hypertension in Morbid-Obesity. Neurology, 1995. **45**(9): p. 1655-1659.

83. Sugerman, H., et al., Intra-abdominal pressure, sagittal abdominal diameter and obesity comorbidity. J Intern Med, 1997. **241**(1): p. 71-79.

84. Whiteley, W., et al., CSF opening pressure: Reference interval and the effect of body mass index. Neurology, 2006. **67**(9): p. 1690-1691.

A randomised controlled trial of bariatric surgery versus a community weight loss programme for the sustained treatment of Idiopathic Intracranial Hypertension: IIH:WT

Trial Registration: ISRCTN 40152829

Statistical Analysis Plan

SAP Version Number
1.0

Author:	Rebecca Woolley	Role:	Trial Statistician	Affiliation:	BCTU<br>University of Birmingham
Signature:		Date:			
Chief Investigator:	Alex Sinclair	Role:	Chief Investigator	Affiliation:	University of Birmingham
Signature:		Date:			
**This Statistical Analysis Plan has been approved by:**					
Approver:	Natalie Ives	Role:	Senior Statistician	Affiliation:	BCTU<br>University of Birmingham
Signature:		Date:			

Statistical Analysis Plan Amendments

SAP version number	Date Approved	Protocol version number†	Section number changed	Description of and reason for change	Timing of change with respect to interim/final analysis	Blind Reviewer	
						Name:	
						Signature:	
						Date:	
						Name:	
						Signature:	
						Date:	
						Name:	
						Signature:	
						Date:	

† This SAP was written based on information contained in the trial protocol version as listed here.

Abbreviations & Definitions

Abbreviation / Acronym	Meaning
BCTU	Birmingham Clinical Trials Unit
BHH	Birmingham Heartlands Hospital
BMI	Body mass index
CONSORT	Consolidated Standards of Reporting Trials
DMC	Data Monitoring Committee
HADS	Hospital Anxiety and Depression Scale
HIT-6	Headache Impact Test 6
ICP	Intracranial pressure
IIH	Idiopathic intracranial hypertension
ISRCTN	International Standard Randomised Controlled Trial Number
ITT	Intention to Treat
LAGB	Laparoscopic adjustable gastric banding
LSG	Laparoscopic sleeve gastrectomy
MDT	Multi-disciplinary team
OCT	Optical coherence tomography
RYGBP	Roux-en-Y gastric bypass
SAE	Serious Adverse Event
SAP	Statistical Analysis Plan
SF-36	Short Form-36
TSC	Trial Steering Committee
**Term**	**Definition**
International Standard Randomised Controlled Trial Number	A clinical trial registry
Protocol	Document that details the rationale, objectives, design, methodology and statistical considerations of the study
Randomisation	The process of assigning trial subjects to intervention or control groups using an element of chance to determine the assignments in order to reduce bias.
Statistical Analysis Plan	Pre-specified statistical methodology documented for the trial, either in the protocol or in a separate document.

23

TABLE OF CONTENTS

1. Introduction 7
2. Background and rationale 7
3. Trial objectives 7
4. Trial methods 8
4.1. Trial design 8
4.2. Trial interventions 8
4.3. Primary outcome measure 9
4.4. Secondary outcome measures 9
4.5. Timing of outcome assessments 10
4.6. Randomisation 10
4.7. Sample size 10
4.8. Framework 11
4.9. Interim analyses and stopping guidance 11
4.10. Internal Pilot Progression Rules 11
4.11. Timing of final analysis 11
4.12. Timing of other analyses 12
4.13. Trial comparisons 12
5. Statistical Principles 12
5.1. Confidence intervals and p-values 12
5.2. Adjustments for multiplicity 12
5.3. Analysis populations 12
5.4. Definition of adherence 12
5.5. Handing protocol deviations and violations 13
5.6. Unblinding 13
6. Trial population 13
6.1. Recruitment 13
6.2. Baseline characteristics 14
7. Intervention(s) 14
7.1. Description of the intervention(s) 14
7.2. Adherence to allocated intervention 14
8. Protocol deviations and violations 14
9. Analysis methods 14
9.1. Covariate adjustment 14
9.2. Distributional assumptions and outlying responses 14
9.3. Handling missing data 15
9.4. Data manipulations 15
9.5. Analysis methods – primary outcome 21
9.6. Analysis methods – secondary outcomes 21
9.7. Analysis methods – exploratory outcomes and analyses 22
9.8. Safety data 22
9.9. Planned subgroup analyses 23
9.10. Sensitivity analyses 23
10. Analysis of sub-randomisations 23
11. Health economic analysis 23
12. Statistical software 23
13. References 23
Appendix A: Deviations from SAP 25
Appendix B: Trial schema 25
Appendix C: Schedule of assessments 27
Appendix D: Template report 29

1. Introduction

This document is the Statistical Analysis Plan (SAP) for the IIH:WT trial, and should be read in conjunction with the current trial protocol. This SAP details the proposed analyses and presentation of the data for the main paper(s) reporting the results for the IIH:WT randomised controlled trial. There are numerous sub-studies embedded within the IIH:WT trial; the analyses for these will be described in separate documents.

The results reported in these main papers will follow the strategy set out here. Subsequent analyses of a more exploratory nature will not be bound by this strategy, though they are expected to follow the broad principles laid down here. The principles are not intended to curtail exploratory analysis (e.g. to decide cut-points for categorisation of continuous variables), nor to prohibit accepted practices (e.g. transformation of data prior to analysis), but they are intended to establish rules that will be followed, as closely as possible, when analysing and reporting data.

Any deviations from this SAP will be described and justified in the final report or publication of the trial (using a table as shown in Appendix A). The analysis will be carried out by an appropriately qualified statistician, who should ensure integrity of the data during their data cleaning processes.

2. Background and rationale

The background and rationale for the trial are outlined in detail in the protocol. In brief, IIH:WT is a trial in participants who have idiopathic intracranial hypertension (IIH) which is characterised by elevated intracranial pressure (ICP) and papilloedema. IIH is a condition found almost exclusively in obese women (90%), and causes disabling daily headaches and loss of vision, which is severe and permanent in up to 25% of cases.[1] Weight loss has been suggested as a treatment strategy in IIH. In a prospective study of 25 women, the use of a very low calorie diet which led to weight loss and significantly reduced body mass index (BMI) significantly lowered ICP and significantly improved papilloedema, vision and headache symptom.[2]

Weight loss, achieved through intensive dieting, is an effective therapeutic strategy in IIH. However, long-term maintenance of weight loss is poor, which leads to recurrence of symptoms. Bariatric surgery has been shown to be a sustainable approach to weight loss, and so may offer a long-term treatment of IIH. Surgery has advantages over other weight management interventions; weight loss is greater with bariatric surgery than with other weight reducing approaches and weight loss is more likely to be sustained.

3. Trial objectives

The primary objective is to assess if weight loss through bariatric surgery and/or a dietary weight loss programme is an effective treatment for IIH. IIH:WT will evaluate the effectiveness of two methods of weight loss for the treatment of IIH in women with a BMI greater than 35kg/m^2 with active IIH: bariatric surgery vs. dietary weight loss programme. It will:

- Evaluate if weight loss achieved through bariatric surgery reduces ICP and consequently treats patients with IIH.
- Evaluate if bariatric surgery is more effective than a dietary weight loss programme in

reducing ICP and consequently treating patients with IIH.

- Evaluate the long-term effectiveness of bariatric surgery versus a dietary weight loss programme in reducing ICP and consequently treating patients with IIH.

Secondary aims are to evaluate the clinical effectiveness, cost-effectiveness and participant-centred clinical outcomes (e.g. quality of life) of bariatric surgery versus a dietary weight loss programme.

4. Trial methods

4.1. Trial design

IIH:WT is a prospective, open-label, parallel group, randomised controlled trial where participants with IIH will be randomised in a 1:1 ratio to an NHS bariatric surgery pathway or to a community based Weight Watchers dietary weight loss programme. See Appendix B for Study Schema.

The trial will necessarily be open-label due to the nature of the intervention, though assessors of the visual outcomes will be masked to randomised treatment allocation. The primary outcome, ICP, is not a subjective measure.

4.2. Trial interventions

Participants randomised to the bariatric surgery arm of the trial will be referred to the local NHS bariatric surgical pathway and, if judged suitable according to the bariatric surgery clinic's screening processes, will undergo laparoscopic adjustable gastric banding (LAGB), Roux-en-Y gastric bypass (RYGBP) or laparoscopic sleeve gastrectomy (LSG). The decision of which surgery to undergo will be made between the surgeon and participant based on the participant's health circumstances and preference.

As way of illustration, the pathway followed at Birmingham Heartlands Hospital (BHH) will be described here. Initially the participant will be seen in the weight management clinic for medical and psychological assessment for bariatric surgery. This assessment period will last as long as the weight management team find appropriate. Once the weight management team are satisfied that the participant is suitable, they will be discussed in the joint multi-disciplinary team (MDT) meeting prior to attending a group session for education regarding bariatric surgery. The participant will then have an outpatient appointment with the Consultant Bariatric Surgeon and given a date for surgery. They will be given up to 12 weeks for further consideration of the procedure if they require it. It is envisioned that the standard patient pathway will take approximately 4 months from randomisation to surgery.

Participants randomised to the dietary weight loss programme arm will be provided with vouchers that exempt them from paying for 52 consecutive and specified weeks of their local Weight Watchers. They will be given the vouchers in batches covering 12 sessions at baseline, 3, 6 and 9 months; these will allow access to 12 sessions in the weekly meetings and to Weight Watchers online and mobile tools. Attendance at the groups will be monitored through participant self-reporting.

4.3. Primary outcome measure

The primary outcome is to examine the effect of bariatric surgery on ICP, as measured by lumbar puncture in cmCSF at 12 months. The primary outcome measure is the difference in ICP at 12 months.

4.4. Secondary outcome measures

Secondary outcomes are as follows:

- ICP at 24 and 60 months;
- Reported IIH symptoms (presence or absence of tinnitus, visual loss, diplopia, visual obscurations and headache) at 12 months (and at 24 and 60 months);
- Visual function in both eyes (measured by LogMAR chart to assess visual acuity, automated perimetry (Humphrey 24-2 central threshold) to measure the visual field mean deviation, a MARS chart to evaluate contrast sensitivity, and Ishihara charts to measure colour vision) at 12 months (and at 24 and 60 months);
- Papilloedema in both eyes (measured by masked assessment of fundus photography and by Optical Coherence Tomography scans (OCT)) at 12 months (and at 24 and 60 months);
- Headache associated disability using the headache impact test-6 score (HIT-6) and headache diary at 12 months (and at 24 and 60 months);
- Anthropometric measures (e.g. waist, hip, fat mass, blood pressure) at 12 months (and at 24 and 60 months);
- Quality of life (participant reported using the EQ-5D-5L, Short Form-36 (SF-36) version 1 questionnaire, Hospital Anxiety and Depression Scale (HADS) and Allodynia Symptom Checklist-12) at 12 months (and at 24 and 60 months);
- Difference in number of referrals to CSF shunting procedures and optic nerve sheath fenestration between treatment arms at 12 months (and at 24 and 60 months).

Fundus photographs will be reviewed by the virtual reading centre which includes three masked neuro-ophthalmologists who will grade the images. They will assign a Frisen grading to each image, and will score the paired (baseline and each follow-up) papilloedema images as better/same/worse. The Frisen grading is scored on a 0 to 5 integer scale with 0=normal optic disc and 5=severe papilloedema. A consensus grading and score will be used for the analysis. Where at least two of the neuro-ophthalmologists are in agreement in the Frisen grading and/or score, then these values will be considered the consensus values. Where this is not the case and there is disagreement across the neuro-ophthalmologists in the Frisen grading and/or score, the reviewers will meet (in person or by video conference) to discuss and arrive at a consensus grading and score.

The following participant completed headache evaluation and generic quality of life questionnaires will be used:

- Headache associated disability using the HIT-6 score (score ranges from 36=best outcome to 78=worst outcome);
- EQ-5D 5L index score (score ranges from -0.281=worst outcome to 1=best outcome);
- EQ-5D health thermometer (score ranges from 0=worst outcome to 100=best outcome);
- SF-36 score (score ranges from 0=worst outcome to 100=best outcome);

- HADS (score ranges from 0=best outcome to 21=worst outcome; 0-7=normal, 8-10=borderline abnormal, 11-21=abnormal);
- Allodynia symptom checklist-12 (score ranges from 0=no symptoms to 24=severe symptoms; 0-2=no allodynia, 3-5=mild allodynia, 6-8=moderate allodynia, 9 or more=severe allodynia).

Note: In the protocol, the ICECAP-A is included in the list of participant completed quality of life questionnaires. This questionnaire forms part of the health economic analysis, and so will not be analysed as part of this SAP.

The Allodynia symptom checklist is completed at each assessment as part of the headache diary and then also as part of the participant booklet. The Allodynia symptom checklist in the headache diary gives an at ictus (i.e. maximum severity headache) score and the participant booklet gives an inter-ictal (i.e. between headache) score. These will be analysed separately.

The Headache Diary is completed over 7 days at baseline (participants complete a daily headache diary in the week before the baseline/screening visit) and again at 12, 24 and 60 months (participants complete a daily headache diary in the week prior to visit). Severity (0-5)* and duration (over each 24 hour period) of headache are reported for each day, along with the use of analgesia.
*Headache diary severity score changed to 0-10 during the trial (see section 9.4).

See Appendix C for assessment time points.

4.5. Timing of outcome assessments

The schedule of trial procedures and outcome assessments are given in Appendix C.

4.6. Randomisation

Participants will be randomised in a 1:1 ratio to either bariatric surgery or dietary weight loss programme.

Randomisation will be provided by a computer generated allocation list held centrally at the Birmingham Clinical Trials Unit (BCTU). The randomisation will be stratified by the following variable:

- Whether or not the patient is taking acetazolamide at entry (yes or no).

4.7. Sample size

The trial aims to randomise 64 participants, 32 participants to each arm (bariatric surgery versus dietary weight loss programme).

For this study, we are hypothesising that the greater weight loss anticipated in the bariatric surgery arm compared to the dietary weight loss arm will consequently reduce the ICP further in the bariatric arm than in the dietary weight loss arm. Bariatric surgery patients typically lose 31% ± 3% of body weight by 12 months.[3] A weight loss of 15.3% ± 7.0% of body weight over 3 months was achieved by patients following a low calorie diet.[2] Data from this study

showed that ICP was significantly reduced by 20% (ICP at baseline in 20 IIH patients was 39.8 ± 5.1 cmCSF and ICP was reduced by 8 ± 4.2 cmCSF, $p<0.001$).

If we assume a conservative change of ICP in the bariatric surgery arm to that previously observed of 8 cmCSF and a change of 3cmCSF in the dietary weight loss arm (a value to reflect changes slightly greater than the baseline fluctuations seen in our previous study), then we are looking to detect a mean difference of 5cmCSF between the groups. To detect this difference of 5cmCSF with 90% power and alpha=0.05 using a 2-sided t-test (assuming a standard deviation of 5.1) requires a total of 46 patients (23 per arm). If we allow for a 28% drop out rate, then we will need to recruit 32 patients per arm, 64 patients in total.

It is believed that the SD of 5.1 is a true reflection of the variability of the data as this is taken from the baseline measurements from our previous study, which is a similar population to that which will be recruited into this study.[2] However, this assumption for the sample size calculation will be monitored during the trial as part of the interim analyses.

4.8. Framework

The objective of the trial is to test the superiority of one intervention to another.

The null hypothesis is that there is no difference in ICP between the intervention groups. The alternative hypothesis is that there is a difference between the groups.

4.9. Interim analyses and stopping guidance

If one treatment arm is more effective with respect to the primary endpoint than the other, then this may become apparent before the target recruitment has been reached. Alternatively, new evidence might emerge from other sources that one of the treatment arms is definitely effective. To protect for this, during the period of recruitment to the trial, interim analyses of major endpoints will be supplied, in strict confidence, to the independent Data Monitoring Committee (DMC) along with updates on results of other related studies, and any other analyses that the DMC may request.

The DMC will advise the chair of the Trial Steering Committee (TSC) if, in their view, either of the randomised comparison in the trial has provided both (a) "proof beyond reasonable doubt†" and (b) evidence that might reasonably be expected to influence the patient management of many clinicians who are already aware of the other main trial results.

†Appropriate criteria of proof beyond reasonable doubt cannot be specified precisely, but a difference of at least $p<0.001$ (similar to Haybittle-Peto boundary) in an interim analysis of a major endpoint may be needed to justify halting, or modifying, the study prematurely. If this criterion were to be adopted, it would have the practical advantage that the exact number of interim analyses would be of little importance, so no fixed schedule is proposed.

4.10. Internal Pilot Progression Rules

Not applicable.

4.11. Timing of final analysis

The primary analysis for the study will occur once all participants have completed the 12

month assessment and the corresponding outcome data has been entered onto the study database and validated as being ready for analysis. This is provided the study has not stopped recruitment early for any reason (e.g. DMC advice or funding body request).

4.12. Timing of other analyses

Data is also being collected at 24 and 60 months, therefore longer-term analysis will occur after the last patient has reached 60 months. The analysis methods described in this SAP will be used for analyses performed at this time point.

4.13. Trial comparisons

All references in this document to 'group' or 'arm' refer to Bariatric Surgery or Dietary Weight Loss Programme, the two treatment arms in the trial.

5. Statistical Principles

5.1. Confidence intervals and p-values

All estimates of differences between groups will be presented with two-sided 95% confidence intervals, unless otherwise stated. P-values will be reported from two-sided tests at the 5% significance level.

5.2. Adjustments for multiplicity

No correction for multiple testing will be made.

5.3. Analysis populations

All primary analyses (primary and secondary outcomes including safety outcomes) will be by intention-to-treat (ITT). Participants will be analysed in the intervention group to which they were randomised, and all participants shall be included whether or not they received the allocated intervention. This is to avoid any potential bias in the analysis.

A per protocol analysis will also be carried out for the primary outcome. See section 5.4 for definition of adherence and hence the per protocol group. See section 9.10 for further details on planned sensitivity analyses.

5.4. Definition of adherence

The expected standard patient pathway for bariatric surgery is expected to take 4 months from randomisation to surgery. Therefore, in the majority of cases, patients should have received bariatric surgery by 4 months after randomisation, but in some cases it could be longer. For the assessment of adherence to surgery within the IIH:WT trial, a patient is considered adherent if they receive bariatric surgery within 12 months of randomisation.

In the dietary weight loss programme arm, participants' attendance to the Weight Watchers sessions will be monitored through self-reporting, but there is not a percentage threshold for which a patient will be considered non-adherent. This is because this arm is acting as the reference group. A patient in the dietary weight loss programme arm who receives bariatric surgery will be considered a cross-over patient (and thus classed as non-adherent).

Within IIH:WT, the per-protocol analysis population is defined as:

Per-protocol population:
Bariatric surgery: Participants who had surgery, and surgery was received within 12 months of randomisation.
Dietary weight loss programme: Participants who did not have bariatric surgery by 12 months.

5.5. Handing protocol deviations and violations

A protocol deviation/violation is defined as a failure to adhere to the protocol such as errors in applying the inclusion/exclusion criteria, the incorrect intervention being given, incorrect data being collected or measured, follow-up visits outside the visit window or missed follow-up visits. We will apply a strict definition of the ITT principle and will include all participants as per the ITT population described in section 5.3 in the analysis in some form regardless of deviation from the protocol.[4] This includes participants who were randomised but later found to violate the inclusion or exclusion criteria. This does not include those participants who have specifically withdrawn consent for the use of their data in the first instance; however these outcomes will be explored as per other missing responses.

Many of the participants taking part in the study will be of childbearing age. Pregnancy is a study exclusion criteria and it is recommended that patients do not become pregnant within a year of surgery, so women planning pregnancy will not be recruited. However, if a trial participant becomes pregnant during the trial, these patients will be excluded from further intervention (will not receive surgery if becomes pregnant before surgical intervention if randomised to the surgery arm, or will discontinue with the weight watchers programme if randomised to the dietary weight loss programme arm). To allow an ITT analysis to be undertaken, these patients will be followed up as per trial protocol where possible. However, if a patient is pregnant, it will not be possible to collect data on ICP, as a lumbar puncture cannot be performed if the patient is pregnant. In these cases, data on ICP will be collected at the earliest possible date post-partum.

The primary outcome is ICP at 12 months. All data will be included in the primary analysis regardless of the time the assessment was completed. A sensitivity analysis where patients whose ICP was collected early (with early defined as >1.5 months before the 12 month assessment date) or late (due to pregnancy or other reasons, with late defined as >3 months post the 12 month assessment date) are excluded from the analysis will be undertaken (see section 9.10).

5.6. Unblinding

Not applicable, IIH:WT is an open-label study.

6. Trial population

6.1. Recruitment

A flow diagram (as recommended by CONSORT[5]) will be produced to describe the participant flow through each stage of the trial. This will include information on the number (with reasons) of losses to follow-up (drop-outs and withdrawals) over the course of the trial. A template for reporting this is given in the Template Final Report.

6.2. Baseline characteristics

The study population will be tabulated as per the Template Final Report. Categorical data will be summarised by number of participants, counts and percentages. Continuous data will be summarised by the number of participants, mean and standard deviation if deemed to be normally distributed or number of participants, median and interquartile range if data appear skewed, and ranges if appropriate. Tests of statistical significance will not be undertaken, nor confidence intervals presented.[6]

7. Intervention(s)

7.1. Description of the intervention(s)

Information on the type of bariatric surgery received and timing of surgery will be reported. A template for reporting this information is given in the Template Final Report.

7.2. Adherence to allocated intervention

A tabulation of those randomised to the bariatric surgery arm who received bariatric surgery and those who did not have bariatric surgery will be produced. A separate table will tabulate attendance at the Weight Watchers sessions for those in the dietary weight loss programme arm. A template for reporting this information is given in the Template Final Report.

8. Protocol deviations and violations

Frequencies and percentages by group will be tabulated for the protocol deviations and violations as per the Template Final Report.

9. Analysis methods

Intervention groups will be compared using generalised estimating equations, or a similar method, to adjust for all covariates as specified in section 9.1, where possible.

9.1. Covariate adjustment

In the first instance, intervention effects between groups for all outcomes will be adjusted for the stratification parameter listed in section 4.6 (whether or not the participant is taking acetazolamide at entry – yes or no), unless otherwise stated. Other covariate adjustment will be baseline values for parameters where available (e.g. analysis of ICP at 12 months will also include the baseline ICP as a covariate in the model). If covariate adjustment results in problems with the model converging, then the stratification variable will be removed from the model first. If model convergence is still a problem then unadjusted estimates will be produced. It will be made clear in the final report why this occurred (e.g. not possible due to low event rate / lack of model convergence).

9.2. Distributional assumptions and outlying responses

Distributional assumptions (e.g. normality of regression residuals for continuous outcomes) will be assessed visually prior to analysis; although in the first instance the proposed primary method of estimation in this analysis plan will be followed. If responses are considered to be particularly skewed and/or distributional assumptions violated, the impact of this will be

examined through sensitivity analysis; this will consist of transformation of responses prior to analysis (e.g. log transformation) in the first instance. If extreme values are apparent and considered to be affecting the integrity of the analysis, a sensitivity analysis consisting of removing the outlying response(s) and repeating the analysis will be performed. Output from these analyses, if performed, will be described and presented alongside the original analysis (or included, e.g. in appendices) with the excluded values clearly labelled.

9.3. Handling missing data

In the first instance, analysis will be completed on received data only with every effort made to follow-up participants even after protocol violation to minimise any potential for bias. To examine the possible impact of missing data on the results, and to make sure we are complying with the ITT principle, sensitivity analysis will be performed on the primary outcome measure.[7] See section 9.10 for further details.

9.4. Data manipulations

The Trial Statistician will derive all responses from the raw data recorded in the database. The specifics of the data manipulations required are as follows.

Scoring for the participant questionnaires is detailed below:

- **HIT-6**
  The HIT-6 response scales are coded as follows:
  - Question 1: Never=6, Rarely=8, Sometimes=10, Very often=11, Always=13
  - Question 2: Never=6, Rarely=8, Sometimes=10, Very often=11, Always=13
  - Question 3: Never=6, Rarely=8, Sometimes=10, Very often=11, Always=13
  - Question 4: Never=6, Rarely=8, Sometimes=10, Very often=11, Always=13
  - Question 5: Never=6, Rarely=8, Sometimes=10, Very often=11, Always=13
  - Question 6: Never=6, Rarely=8, Sometimes=10, Very often=11, Always=13

  A total score is calculated by summing each question score as follows:

  - Total=SUM(Question1, Question2, Question3, Question4, Question5, Question6)

  HIT-6 ranges from 36-78 where a low score indicates no impact of headache.

- **EQ-5D (5 level)**[8]
  The EQ-5D (5 level) response scales are coded as follows:

  - Mobility: I have no problems in walking about=0, I have slight problems in walking about=0.051, I have moderate problems in walking about=0.063, I have severe problems in walking about=0.212, I am unable to walk about=0.275

- Self-care: I have no problems washing or dressing myself=0, I have slight problems washing or dressing myself=0.057, I have moderate problems washing or dressing myself=0.076, I have severe problems washing or dressing myself=0.181, I am unable to wash or dress myself=0.217

- Usual activities: I have no problems doing my usual activities=0, I have slight problems doing my usual activities=0.051, I have moderate problems doing my usual activities=0.067, I have severe problems doing my usual activities=0.174, I am unable to do my usual activities=0.190

- Anxiety: I am not anxious or depressed=0, I am slightly anxious or depressed=0.079, I am moderately anxious or depressed=0.104, I am severely anxious or depressed=0.296, I am extremely anxious or depressed=0.301

- Pain: I have no pain or discomfort=0, I have slight pain or discomfort=0.060, I have moderate pain or discomfort=0.075, I have severe pain or discomfort=0.276, I have extreme pain or discomfort=0.341.

The EQ-5D (5 level) index score is derived as follows:

- Index score = 1-0.9675xSUM(Mobility, Self-care, Usual activities, Anxiety, Pain)

The EQ-5D (5 level) index score ranges from –0.281 to 1, where a score of 1 implies perfect health, a score of 0 implies a health status of death and negative scores imply a health status worse than death. No missing data items are permitted in order to compute a score.

- **SF-36 (v1)**[9]

  The SF-36 response scales are coded as follows:

  - Question 1: Excellent=100, Very good=75, Good=50, Fair=25, Poor=0

  - Question 2: Much better than 1 year ago=100, Somewhat better than 1 year ago=75, About the same=50, Somewhat worse now than 1 year ago=25, Much worse now than 1 year ago=0

  - Questions 3-12: Yes limited a lot=0, Yes limited a little=50, No not limited at all=100

  - Questions 13-19: Yes=0, No=100

  - Question 20: Not at all=100, Slightly=75, Moderately=50, Quite a bit=25, Extremely=0

- Question 21: None=100, Very mild=80, Mild=60, Moderate=40, Severe=20, Very severe=0
- Question 22: Not at all=100, Slightly=75, Moderately=50, Quite a bit=25, Extremely=0
- Questions 23, 26, 27, 30: All of the time=100, Most of the time=80, A good bit of the time=60, Some of the time=40, A little of the time=20, None of the time=0
- Questions 24, 25, 28, 29, 31: All of the time=0, Most of the time=20, A good bit of the time=40, Some of the time=60, A little of the time=80, None of the time=100
- Question 32: Not at all=100, Slightly=75, Moderately=50, Quite a bit=25, Extremely=0
- Questions 33, 35: Definitely true=0, Mostly true=25, Don't know=50, Mostly false=75, Definitely false=100
- Questions 34, 36: Definitely true=100, Mostly true=75, Don't know=50, Mostly false=25, Definitely false=0.

The SF-36 domain scores listed below will be derived from summing the items in that domain and dividing by the number of items in that domain as follows:

- Physical functioning = SUM(3-12)/10
- Role limitations due to physical health = SUM(13-16)/4
- Role limitations due to emotional problems = SUM(17-19)/3
- Energy/fatigue = SUM(23, 27, 29, 31)/4
- Emotional well-being = SUM(24, 25, 26, 28, 30)/5
- Social functioning = SUM(20, 32)/2
- Pain = SUM(21, 22)/2
- General health = SUM(1, 33-36)/5.

The SF-36 scores range from 0-100 where lower scores suggest greater presence of

limitations in that domain, and no missing data is permitted in order to compute a score.

- **HADS**[10]
  The HADS response scales are coded as follows:

  - Question 1 (I feel tense or 'wound up'): Not at all=0, From time to time occasionally=1, A lot of the time=2, Most of the time=3

  - Question 2 (I feel as if I am slowed down): Not at all=0, Sometimes=1, Very often=2, Nearly all the time=3

  - Question 3 (I still enjoy the things I used to enjoy): Definitely as much=0, Not quite so much=1, Only a little=2, Hardly at all=3

  - Question 4 (I get a sort of frightened feeling like 'butterflies' in the stomach): Not at all=0, Occasionally=1, Quite often=2, Very often=3

  - Question 5 (I get a sort of frightened feeling as if something awful is about to happen): Not at all=0, A little but it doesn't worry me=1, Yes but not too badly=2, Very definitely and quite badly=3

  - Question 6 (I have lost interest in my appearance): I take just as much care as ever=0, I may not take quite as much care=1, I don't take as much care as I should=2, Definitely=3

  - Question 7 (I can laugh and see the funny side of things): As much as I always could=0, Not quite so much now=1, Definitely not so much now=2, Not at all=3

  - Question 8 (I feel restless as if I have to be on the move): Not at all=0, Not very much=1, Quite a lot=2, Very much indeed=3

  - Question 9 (Worrying thoughts go through my mind): Very Little=0, From time to time but not too often=1, A lot of the time=2, A great deal of the time=3

  - Question 10 (I look forward with enjoyment to things): As much as I ever did=0, Rather less than I used to=1, Definitely less than I used to=2, Hardly at all=3

  - Question 11 (I feel cheerful): Most of the time=0, Sometimes=1, Not often=2, Never=3

  - Question 12 (I get sudden feelings of panic): Not at all=0, Not very often=1, Quite often=2, Very often indeed=3

- Question 13 (I can sit at ease and feel relaxed): Definitely=0, Usually=1, Not often=2, Not at all=3

- Question 14 (I can enjoy a good book or radio or television programme): Often=0, Sometimes=1, Not often=2, Very seldom=3

The HADS subscale scores listed below will be derived from summing the items in that subscale, as follows:

- Anxiety subscale = SUM(1, 4, 5, 8, 9, 12, 13)

- Depression subscale = SUM(2, 3, 6, 7, 10, 11, 14)

The HADS subscale scores range from 0 to 21, where low scores are good and high scores are bad. One missing data item is permitted in this subscale and is computed at the participant level as follows:

Subscale = (SUM(completed items in subscale)/6)*7

The HADS subscale scores can also be dichotomised using cut-off values as follows:

- Normal 0-7
- Borderline Abnormal 8-10
- Abnormal 11-21.

- **Allodynia symptom checklist-12**
  The allodynia symptom checklist response scales are coded as follows:

  - Combing your hair: Does not apply=0, Never=0, Rarely=0, Less than half the time=1, Half the time or more=2

  - Pulling your hair back: Does not apply=0, Never=0, Rarely=0, Less than half the time=1, Half the time or more=2

  - Shaving your face: Does not apply=0, Never=0, Rarely=0, Less than half the time=1, Half the time or more=2

  - Wearing eyeglasses: Does not apply=0, Never=0, Rarely=0, Less than half the time=1, Half the time or more=2

  - Wearing contact lenses: Does not apply=0, Never=0, Rarely=0, Less than half the time=1, Half the time or more=2

  - Wearing earrings: Does not apply=0, Never=0, Rarely=0, Less than half the time=1, Half the time or more=2

  - Wearing necklace: Does not apply=0, Never=0, Rarely=0, Less than half the time=1,

Half the time or more=2

- Wearing tight clothing: Does not apply=0, Never=0, Rarely=0, Less than half the time=1, Half the time or more=2

- Taking a shower: Does not apply=0, Never=0, Rarely=0, Less than half the time=1, Half the time or more=2

- Resting your face or head on a pillow: Does not apply=0, Never=0, Rarely=0, Less than half the time=1, Half the time or more=2

- Exposure to heat: Does not apply=0, Never=0, Rarely=0, Less than half the time=1, Half the time or more=2

- Exposure to cold: Does not apply=0, Never=0, Rarely=0, Less than half the time=1, Half the time or more=2

The total score is calculated by summing the values for each question.

The score ranges from 0 to 24 where a low score is good

The allodynia scores can also be dichotomised using cut-off values as follows:

0-2 = no allodynia
3-5 = mild allodynia
6-8 = moderate allodynia
9 or more = severe allodynia

- **Headache diary data**
  The Headache Diary is completed over 7 days prior to the assessment visit with information on severity and duration of headache, and use of analgesia recorded.

  - The overall headache severity score for each participant is calculated by summing the severity score reported on each day that a headache was reported, divided by the number of days that a headache was reported. There are 2 versions of the headache diary. The first version scored headache severity on a 0-5 scale. The second version scored headache severity on a 0-10 scale. In both cases, a score of zero means no headache and does not contribute to the severity score. To combine the scores, the first version using the 0-5 scores will be mapped onto the second version scores using the following method: 0=0; 1=2; 2=4; 3=6; 4=8; and 5=10.

  - The overall duration of headache (hours) for each participant is calculated by summing the duration of headache reported on each day that a headache was reported, divided by the number of days that a headache was reported.

  - Headache frequency is calculated as the number of days a headache is experienced that week. In line with international Headache society reporting guidelines on headache

outcomes which recommends reporting headache frequency per month, the weekly headache frequency will then be multiplied by 4 to provide a headache frequency per month (which will then be comparable with other trial datasets).

- Similarly, analgesic use will be calculated by summing up the number of times they are used over a week, then multiplied by 4 to provide analgesic use per month.

For headache frequency and analgesic use, if there is missing data in the headache diary, meaning that an incomplete week of headache data is available, the data reported will be extrapolated to calculate a full week value before being multiplied by 4 to obtain the frequency by month. For example, if a patient reports the following:

Headache, missing, No Headache, Headache, missing, Headache, No Headache. This corresponds to 3 days of headache over 5 days. This would be extrapolated to a week by 3/5 x 7 days = 4.2 days of headache/week. Then 4.2 x 4 = 16.8 days/month.

Other outcomes will be calculated as follows:

- Age – number of days from date of birth to randomisation date divided by 365.25 to give age in years;
- Duration of IIH – number of days from date of diagnosis of IIH to randomisation date divided by 30.4 to give duration of IIH in months;
- Blood pressure – take average of 2nd and 3rd measure if present. If only one set of BP measures reported then these values will be used;
- Length of stay – the number of days from date of surgery to date of discharge;
- Worst eye – the eye that has the worst Mean Deviation (most negative value) score on the Humphrey Visual field assessment at baseline will be considered the 'worst eye' for all analyses. Unless that eye has a Frisen grade of zero, in which case the other eye will be considered the worse eye;
- Best (fellow) eye – the eye that has the better Mean Deviation (most positive score) score on the Humphrey Visual field assessment at baseline will be considered the 'best eye' for all analyses;
- Ishihara colour assessment – number of correct plates / number of plates x 100%.

9.5. Analysis methods – primary outcome

A template for reporting the primary outcome is given in the Template Final Report.

The primary outcome is the ICP at 12 months and will be summarised using means and standard deviations. A linear regression model will be used to compare the ICP at 12 months between the two arms, with baseline ICP and the stratification variable in section 4.6 included as covariates in the model, where possible, with the adjusted mean difference between groups presented alongside the 95% confidence interval.

9.6. Analysis methods – secondary outcomes

A template for reporting the secondary outcomes is given in the Template Final Report.

Continuous data items (e.g. HIT-6, anthropometric data) will be analysed in the same way as the primary outcome. Analyses will be performed on data at 12, 24 and 60 months. For the visual function and papilloedema (Frisen grading) data which is collected in both eyes, data will be presented for the worst eye and the best eye separately (see section 9.4), and analysed as per the primary outcome.

The Fundus photographs at 12, 24 and 60 months are also compared to the baseline photograph to assess whether the image is better, worse or the same as the baseline photograph (see section 4.4). The same, better or worse data will be presented for the worst eye and the best eye separately, and analysed at 12, 24 and 60 months using a chi-squared test.

The IIH symptom data is binary. The number and percentage of participants experiencing each symptom will be presented at baseline and 12, 24 and 60 months by treatment arm. Log-binomial models will be used to compare the symptom data between the two arms at 12, 24 and 60 months, with current IIH symptom and the stratification variable in section 4.6 included as covariates in the model, where possible, with the adjusted relative risk presented alongside the 95% confidence interval.

Similarly, the number of referrals to CSF shunting procedures and optic nerve sheath fenestration is binary. Data will be presented at 12, 24 and 60 months by treatment arm. Log-binomial models will be used to compare the data between the two arms at 12, 24 and 60 months, with the stratification variable in section 4.6 included as a covariate in the model, where possible, with the adjusted relative risk presented alongside the 95% confidence interval.

9.7. Analysis methods – exploratory outcomes and analyses

Any data that does not form an outcome will be presented using simple summary statistics by treatment group (i.e. numbers and proportions for binary data and mean (median) and standard deviation (inter-quartile range) for continuous normal (non-normal) data.

We are hypothesising that those in the bariatric surgery arm will have a lower ICP than those in the dietary weight loss programme arm at 12 months. This is based on the fact that weight loss in the bariatric surgery arm should be greater than those in the dietary weight loss programme arm. To assess association between weight loss and ICP, scatter plots of change in weight and change in ICP (between baseline and 12 months), change in BMI and change in ICP (between baseline and 12 months) and percentage weight loss and change in ICP (between baseline and 12 months) will be presented and a Spearman's correlation coefficient by treatment group will be produced.

9.8. Safety data

The number and percentage of patients experiencing any serious adverse events (SAEs) will be presented by group. Statistical significance will be determined by chi-squared test. No other formal analysis is anticipated due to the low anticipated frequency of events. A detailed descriptive table of all SAEs will be produced including the proportion and percentage of those determined to be treatment related (causality) by group. A template for reporting is given in the Template Final Report.

9.9. Planned subgroup analyses

There are no planned subgroups analyses as part of this SAP.

9.10. Sensitivity analyses

Sensitivity analyses will be limited to the primary outcome and will consist of:

- Per-protocol analyses (using the per-protocol analysis population described in section 5.4);
- An analysis to assess the effect of missing responses using last observation carried forward (the primary outcome is only collected in both groups at baseline and 12 months, so this is essentially carrying forward the baseline ICP to 12 months, which is essentially assuming no change) and multiple imputation (with the following variables used baseline ICP, BMI, mean deviation, OCT, on acetazolamide at entry (stratification variable) and randomised treatment);
- An analysis to assess the effect of ICP data collected outside the time window by excluding ICP values that are -1.5 or +3 months outside the 12 month assessment point;
- An analysis to assess the effect of any technical errors with the visual tests on the outcome mean deviation.[11] If patient has either a false positive value >15% or a false negative value >25% or a fixation loss value >33%, then the mean deviation value for that patient is excluded.

10. Analysis of sub-randomisations

Not applicable.

11. Health economic analysis

As indicated in the protocol there will also be an economic analysis. The details of this analysis are documented separately.

12. Statistical software

SAS software, version 9.4 (or higher) and/or Stata version 15 (or higher) will be used for all analyses.

13. References

1. Scott CJ, Kardon RH, Lee AG, Frisén L, Wall M. Diagnosis and grading of papilledema in patients with raised intracranial pressure using optical coherence tomography vs clinical expert assessment using a clinical staging scale. Arch Ophthalmol. 2010;128(6):705-11.
2. Sinclair AJ, Burdon MA, Nightingale PG, Ball AK, Good P, Matthews TD, et al. Low energy diet and intracranial pressure in women with idiopathic intracranial hypertension: prospective cohort study. BMJ 2010;341:c2701
3. Colquitt JL, Picot J, Loveman E, Clegg AJ. Surgery for obesity. Cochrane Database Syst Rev 2009;(2):CD003641
4. Gupta SK. Intention-to-treat concept: A review. Perspect Clin Res. 2011;2(3):109-112.
5. Schulz KF, Altman DG, Moher D, for the CONSORT Group. CONSORT 2010 Statement: updated guidelines for reporting parallel group randomised trials. BMJ. 2010;340:c332.
6. Altman DG, Dore CJ. Randomisation and baseline comparisons in clinical trials. Lancet.

1990;335:149–53.

7. White IR, Horton NJ, Carpenter J, Pocock SJ. Strategy for intention to treat analysis in randomised trials with missing outcome data. BMJ. 2011;342:d40.
8. Devlin N, Shah K, Feng Y, Mulhern B, van Hout B. Valuing Health-Related Quality of Life: An EQ-5D-5L Value Set for England. Office of Health Economics. 2016, Research Paper 16/01
9. Ware, John E., and Cathy Donald Sherbourne. "The MOS 36-Item Short-Form Health Survey (SF-36): I. Conceptual Framework and Item Selection." *Medical Care*, vol. 30, no. 6, 1992, pp. 473–483. *JSTOR*, JSTOR, www.jstor.org/stable/3765916.
10. Zigmond AS, Snaith RP. The hospital anxiety and depression scale. Acta Psychiatr Scand 1983 Jun;67(6):361-70.
11. Wall M, Johnson CA, Cello KE, et al. Visual Field Outcomes for the Idiopathic Intracranial Hypertension Treatment Trial (IIHTT). Investigative Ophthalmology & Visual Science. 2016;57(3):805-12.

Appendix A: Deviations from SAP

This report below follows the statistical analysis plan dated <insert effective date of latest SAP> apart from following:

**Section of report not following SAP**	**Reason**
<insert section >	<insert, e.g. exploratory analyses request by TMG>

Appendix B: Trial schema

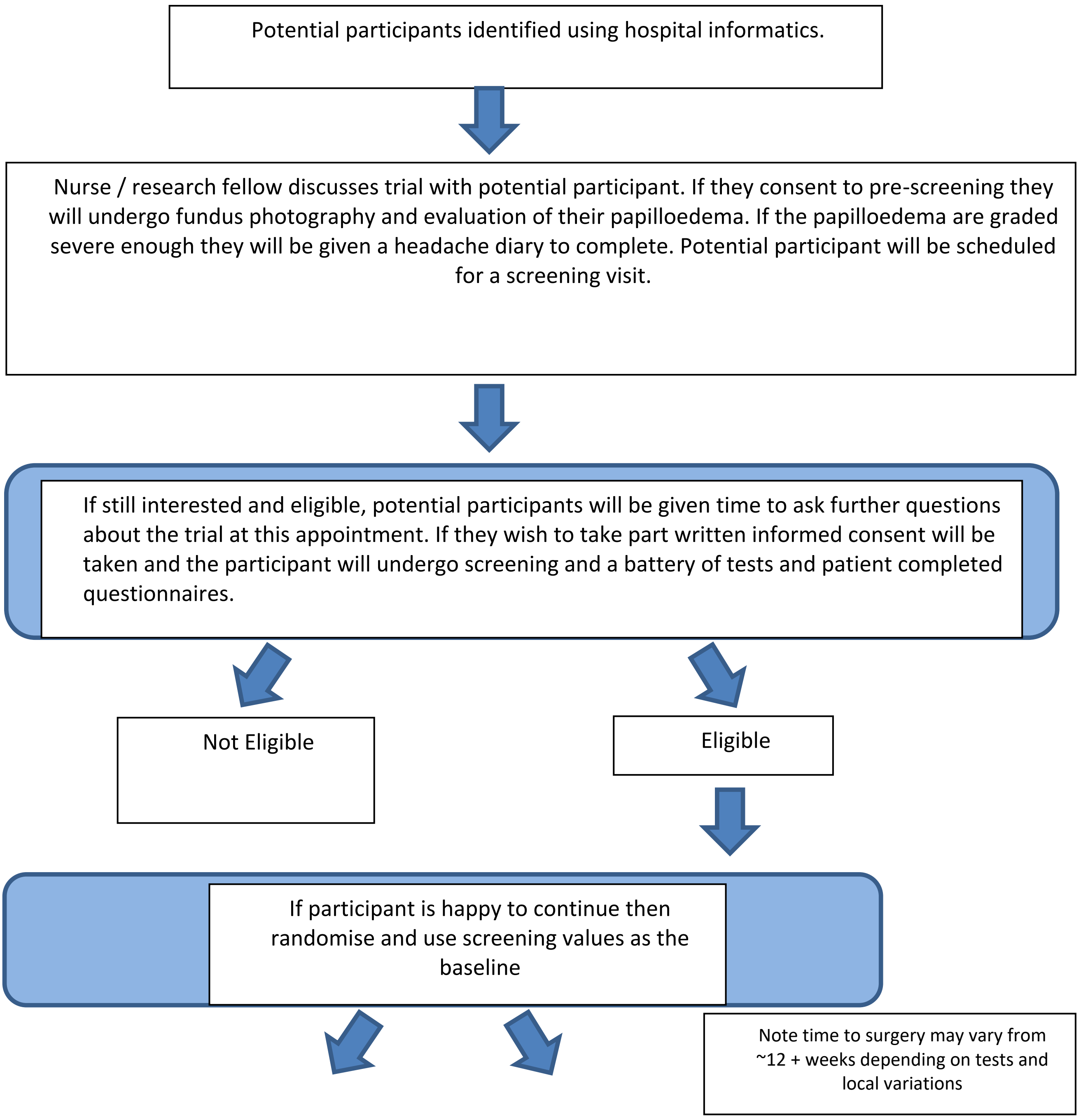

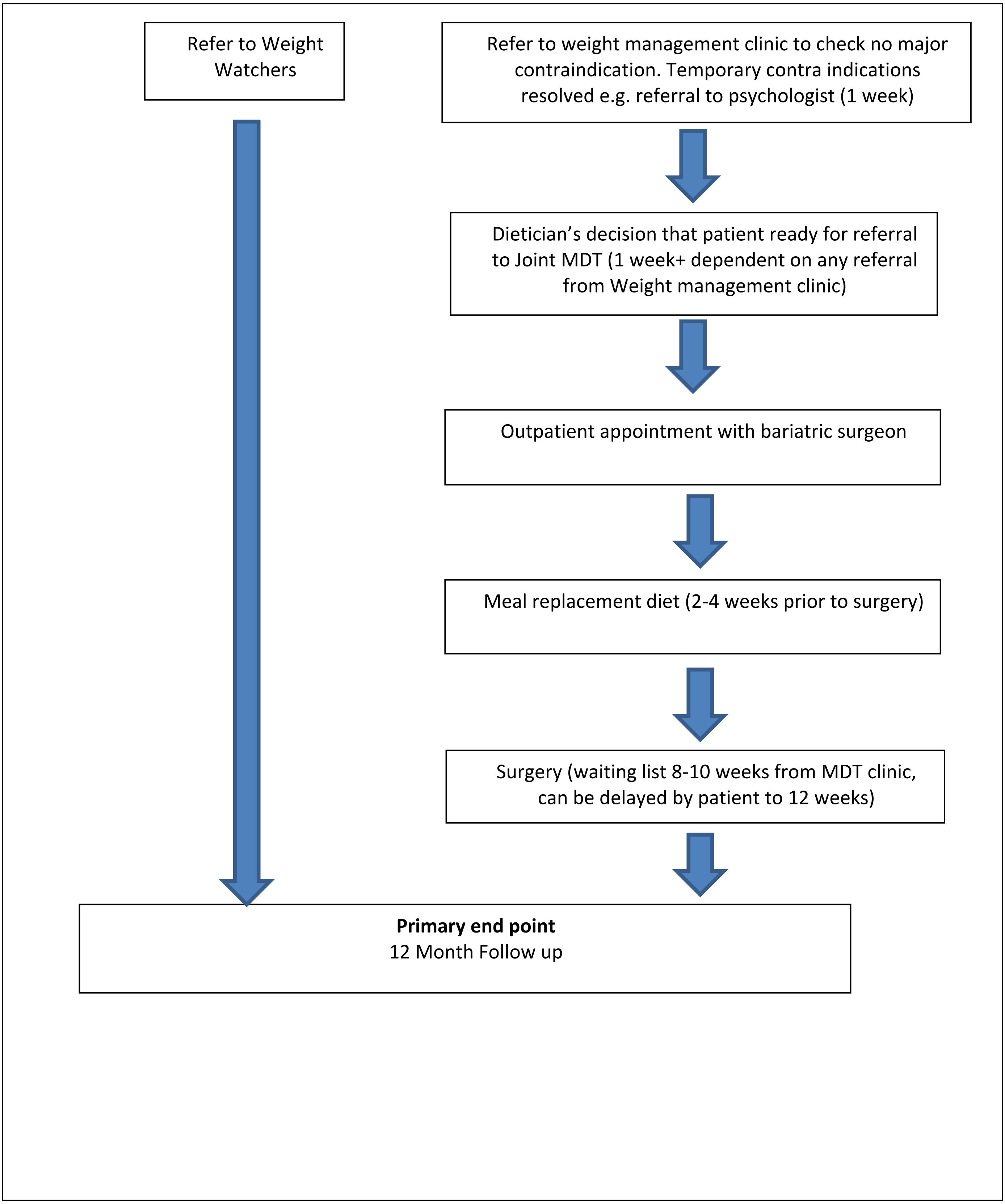
Refer to Weight Watchers
Refer to weight management clinic to check no major contraindication. Temporary contra indications resolved e.g. referral to psychologist (1 week)
Dietician's decision that patient ready for referral to Joint MDT (1 week+ dependent on any referral from Weight management clinic)
Outpatient appointment with bariatric surgeon
Meal replacement diet (2-4 weeks prior to surgery)
Surgery (waiting list 8-10 weeks from MDT clinic, can be delayed by patient to 12 weeks)
Primary end point
12 Month Follow up

Appendix C: Schedule of assessments

Outcome	Measure	Pre-screening visit	Baseline	3 months	6 months	Post-op	(Primary endpoint) 12 months	24 months	60 months
**Primary outcome**									
Intracranial pressure	Lumbar puncture		x			x	x	x	x
**Secondary outcomes**									
Eligibility	Pregnancy test		x						
Weight	BMI		x	x	x	x	x	x	x
	Waist/hip ratio		x	x	x	x	x	x	x
	Blood pressure		x	x	x	x	x	x	x
	Fat Mass		x	x	x	x	x	x	x
Visual assessments	Visual acuity and contrast sensitivity		x				x	x	x
	Humphrey visual field (24-2)		x				x	x	x
	Ishihara colour assessment		x				x	x	x
	Optical coherence tomography		x				x	x	x
	Retinal photographs	x					x	x	x
Headache assessments	Headache Impact Test 6	x					x	x	x
	Post-LP Headache diary		x			x	x	x	x
	Headache diary	x					x	x	x
Quality of Life	EQ-5D-5L		x				x	x	x
	ICECAP-A		x				x	x	x
	SF-36 Version 1		x				x	x	x
	HADS		x				x	x	x

	Allodynia Symptom Checklist-12		x				x	x	x
Health Economics	Cost-effectiveness, -utility and -benefit		x				x	x	x
Biomarkers	Blood		x			x	x	x	x
	CSF		x			x	x	x	x
	Meal stimulation		x			x	x	x	x
Sleep apnoea									
	Epworth Sleepiness Scale, Berlin questionnaire	-	x				x		
	STOP-BANG	x					x		
SAE monitoring	SAE forms		x	x	x	x	x	x	x

Appendix D: Template report

A template report for the final analyses will be provided in a separate document.
