# Supplementary material for: Effectiveness of Bariatric Surgery vs Community Weight Management Intervention for the Treatment of Idiopathic Intracranial Hypertension: A Randomized Clinical Trial
Source: JAMA Neurol. 2021 Apr 26;78(6):678–86. doi: 10.1001/jamaneurol.2021.0659 (PMC8077040; doi:10.1001/jamaneurol.2021.0659)
Supplement: Supplement 2. — eMethods 1. Clinical Trial Investigators and Sites, Bariatric Surgery Pathway Team, Patient Identification Centers, and Study Oversight Committees eMethods 2. Community Weight Management Intervention, Bariatric Surgery Pathway, and Hierarchical Regression Analysis eResults. Relevant Medication Changes Over the Course of the Clinical Trial eTable 1. Inclusion and Exclusion Criteria of the IIH:WT eTable 2. IIH:WT Schedule of Events eTable 3. Secondary Outcome: Headache eTable 4. Secondary Outcome: Visual Data (Worst Eye) eTable 5. IIH Symptoms, Baseline to 12 Months eTable 6. Quality of Life and Hospital Anxiety and Depression Scores eTable 7. Quality of Life Subscales as Measured by the SF-36 eTable 8. Serious Adverse Events at 12 and 24 Months [file jamaneurol-e210659-s002.pdf]

Supplementary Online Content

Mollan SP, Mitchell JL, Ottridge RS, et al. Effectiveness of bariatric surgery vs community weight management for the treatment of idiopathic intracranial hypertension: a randomized clinical trial. *JAMA Neurol.* Published online April 26, 2021. doi:10.1001/jamaneurol.2021.0659

eMethods 1. Clinical Trial Investigators and Sites, Bariatric Surgery Pathway Team, Patient Identification Centers, and Study Oversight Committees

eMethods 2. Community Weight Management Intervention, Bariatric Surgery Pathway, and Hierarchical Regression Analysis

eResults. Relevant Medication Changes Over the Course of the Clinical Trial

eTable 1. Inclusion and Exclusion Criteria of the IIH:WT

eTable 2. IIH:WT Schedule of Events

eTable 3. Secondary Outcome: Headache

eTable 4. Secondary Outcome: Visual Data (Worst Eye)

eTable 5. IIH Symptoms, Baseline to 12 Months

eTable 6. Quality of Life and Hospital Anxiety and Depression Scores

eTable 7. Quality of Life Subscales as Measured by the SF-36

eTable 8. Serious Adverse Events at 12 and 24 Months

This supplementary material has been provided by the authors to give readers additional information about their work.

© 2021 Mollan SP et al. *JAMA Neurology.*

eMethods 1. Clinical Trial Investigators and Sites, Bariatric Surgery Pathway Team, Patient Identification Centers, and Study Oversight Committees

Trial Investigators
Chief Investigator: Professor. Alex Sinclair
Trial Manager: Mr Ryan Ottridge
Trial Statistician: Dr Kristian Brock
Health Economists: Dr Magda Aguiar and Professor Emma Frew

Trial sites and investigators
University Hospitals Birmingham National Health service (NHS) Foundation Trust: Miss Susan Mollan and Mr Tim Mathews.
Manchester Royal Eye Hospital, Manchester University NHS Foundation Trust: Mr Alec Ansons.
Royal Devon and Exeter NHS Foundation Trust: Mr James Benzimra.

Bariatric surgery pathway team
Mr. Rishi Singhal, Mr Paul Super, Mr Markos Daskalakis (Consultant Bariatric & Upper GI Surgeons)
Sally Abbott – Specialist Bariatric Dietician

Patient identification centres
Gloucester Royal Hospital, Gloucestershire Hospitals NHS Foundation Trust: Dr Ben Wakerley.
Leicester General Hospital, University Hospitals of Leicester NHS trust: Dr Mark Lawden.
Royal Stoke University Hospital, University Hospitals of North Midlands NHS Foundation Trust: Dr Brendan Davies.
Royal Hallamshire Hospital, Sheffield Teaching Hospitals NHS Foundation Trust: Dr Simon Hickman.

Study oversight committees
We thank all the members of the study oversight committees for their valued contributions.
The IIH:WT Trial Steering Committee; the Data Monitoring and Ethics Committee and the Trial Management Group.

eMethods 2. Community Weight Management Intervention, Bariatric Surgery Pathway, and Hierarchical Regression Analysis

Community weight management intervention

WeightWatchers^{TM} program was chosen as the community weight management intervention (CWI) because it had superior weight loss[1], was the best attended and most cost-effective[2]. Participants in the CWI arm were given exemption vouchers for 52 consecutive and specified weeks of their local WeightWatchers^{TM} group with access to WeightWatchers^{TM} online and mobile tools for 12 months. Vouchers provided 12 sessions at baseline, 3, 6 and 9 months.

Bariatric surgery pathway

The bariatric surgery pathway participants were screened to ensure their suitability, initially for medical and psychological assessment in the weight management clinic. This assessment continued for as long as thought appropriate, as per routine care. Once suitable, the case was discussed in the joint multi-disciplinary meeting, prior a group session for education regarding surgery. The participant then attended a consultant bariatric surgeon and was given a date for surgery. Twelve weeks was permitted for further consideration of the procedure if required. The standard patient pathway was envisioned to take approximately 4 months. The choice of surgical intervention was decided between the surgeon and participant, based on the participant's health and preference. These included laparoscopic adjustable gastric banding, laparoscopic Roux-en-Y gastric bypass or laparoscopic sleeve gastrectomy.

Hierarchical regression analysis

Initially, hierarchical regression models were generated, with data for both eyes for all patients analyzed in one model, using group structure to distinguish between the eyes. Models contained population-level terms (i.e. terms that apply to each experimental unit) to reflect: 1) the mean baseline value (i.e. the intercept); 2) the mean change from baseline associated with each assessment time (i.e. time as a factor variable); 3) the extra mean change from baseline associated with each assessment time in the experimental arm (i.e. the interaction of treatment allocation and time as a factor variable). Additionally, hierarchical regression models contained random effects (i.e. terms that are specific to each experimental unit) to reflect the random deviations from the population-level mean value at baseline (i.e. random intercepts).

© 2021 Mollan SP et al. *JAMA Neurology.*

eResults. Relevant Medication Changes Over the Course of the Clinical Trial

Headache preventative medication was taken by 27% (18/66) of all patients at enrolment. This reduced markedly amongst the bariatric surgery arm from 36% (12/33) at baseline to 7% (2/30) at 12 months, compared with little difference in the CWI arm from baseline 18% (6/33) to 21% (6/29) at 12 months. Acetazolamide was taken by 29% (19/66) of all patients at enrolment. The number of patients using acetazolamide reduced amongst those in the bariatric surgery arm from 24% (8/33) to 3% (1/30) with a mean daily dose change from 781mg (471.3) to 500mg (standard deviation [SD] 0) compared with the CWI arm whose numbers using acetazolamide reduced from 33% (11/33) to 28% (8/33) with a mean daily dose change from 909mg (SD 550.8) to 844mg (SD 498.9). Topiramate was used by 12% (4/33) in the bariatric surgery arm at baseline. All had discontinued by 12 months, whilst in the CWI arm 6% (2/33) topiramate use increased to 10% (3/29) by 12 months. Those taking other diuretics were 3% (1/33) in bariatric surgery arm and 6% (2/33) in the CWI arm at baseline. By 12 months, 3% (1/30) were using a diuretic in the bariatric surgery arm and 10% (3/29) in the CWI arm. There were no significant differences in the use of antihypertensive medications or hormonal contraception.

© 2021 Mollan SP et al. *JAMA Neurology.*

eTable 1. Inclusion and Exclusion Criteria of the IIH:WT

	Criterion
**Inclusion**	Female patients with IIH aged between 18 and 55 years, diagnosed according to the Friedman Jacobson criteria,[1] who have active disease [papilloedema (Frisén[2] grade ≥1 in at least one eye), significantly raised LP OP ≥25 cmCSF] of over 2 months' duration and no evidence of venous sinus thrombosis (MRI or CT and venography as noted at diagnosis)
	Body mass index of ≥35 kg/m^2.
	Have previously tried other appropriate non-surgical treatments to lose weight but have not been able to achieve or maintain adequate, clinically beneficial weight loss for at least 6 months.
	Able to give informed consent.
**Exclusion**	Age <18 or >55 years.
	Pregnancy, or planning pregnancy.
	Significant comorbidity, Cushing's syndrome, Addison's disease or the use of oral or injected glucocorticoid therapy.
	Previously undergone optic nerve sheath fenestration
	Definite indication for or contraindication against surgery or dieting
	Have a specific medical or psychiatric contraindication for surgery, including drug misuse, eating disorder or major depression (suicidal ideation, drug overdose or psychological admission in the last 12 months).
	Previous bariatric surgery
	Inability to give informed consent, for example, due to cognitive impairment.

References to eTable 1:

1.Friedman DI, Liu GT, Digre KB. Revised diagnostic criteria for the pseudotumor cerebri syndrome in adults and children. *Neurology*. 2013; 81(13):1159-1165.

2. Frisen L. Swelling of the optic nerve head: a staging scheme. *J Neurol Neurosurg Psychiatry.* 1982;45(1):13-18.

© 2021 Mollan SP et al. *JAMA Neurology.*

eTable 2. IIH:WT Schedule of Events

Outcome	Measure	Baseline	3 months	6 months	Postoperative	12 months (primary endpoint)	24 months	60 months
**Intracranial pressure**	Lumbar puncture opening pressure	x			x	x	x	x
**Clinical measures**	Body mass index, blood pressure, waist/hip, fat mass, medication use	x	x	x	x	x	x	x
**Idiopathic Intracranial Hypertension symptoms**	Pulsatile tinnitus, visual loss, diplopia, visual obscurations	x				x	x	x
**Visual function**	Visual acuity, contrast sensitivity, colour assessment	x				x	x	x
	Humphrey visual field (24–2)	x				x	x	x
**Papilledema grade**	Optical coherence tomography	x				x	x	x
	Retinal photographs	x				x	x	x
**Headache**	HIT-6, headache diary	x				x	x	x
**Quality of life**	EQ-5D-5L, ICECAP-A, SF-36 v1, HADS	x				x	x	x
**Health economics**	Resource use questionnaire	x				x	x	x

HIT-6 = Headache impact test-6. EQ-5D-5L= ICECAP-A= SF-36v1=, HADS=Hospital anxiety and depression score.

© 2021 Mollan SP et al. *JAMA Neurology.*

eTable 3. Secondary Outcome: Headache

Differences in headache and visual function outcomes were not significant at 12- or 24-months (eTables 3 and 4). Exploratory analysis noted a greater improvement in monthly headache days, headache severity and HIT-6 scores in the bariatric surgery arm between baseline and 12-months (eTable 3).

	**Baseline**	**12 months**	**24 months**	**Difference baseline to 12 months**	**Difference baseline to 12 months**	**Difference baseline to 24 months**	**Difference between arms at 12 months**	**Difference between arms at 24 months**
					**Hierarchical regression**			
	**Mean (SD), n**			**Mean (SD); 95%CI, p**	**Mean (SE); 95%CI, p**			
**Headache disability (HIT-6)**								
**Community weight management intervention**	64.3 (8.6), 32	59.7 (12.4), 26	60.2 (10.9), 23	-6.2 (9.8), 28	-5.3 (1.9); (-9.1, -1.5), p=0.006	-5.7 (2.0); (-9.7, -1.8), p=0.004	-1.4 (2.6); (-6.6, 3.8), p=0.603	-1.4 (2.8); (-7.0, 4.1), p=0.610
**Bariatric surgery**	65.1 (6.0), 33	57.5 (9.1), 29	56.5 (13.0), 23	-7.4 (8.6), 29	-7.5 (1.9); (-11.1, -3.9), p<0.001	-8.0 (2.0); (-11.9, -4.0), p<0.001		
**Monthly headache days**								
**Community weight management intervention**	22.5 (7.8), 31	16.7 (11.8), 24	15.8 (11.1), 21	-6.3 (9.9), 23	-5.9 (2.2); (-10.2, -1.6), p=0.007	-7.4 (2.3); (-11.9, -2.9), p=0.001	-3.2 (2.8); (-8.6, 2.2), p=0.247	-2.9 (3.0); (-8.7, 2.9), p=0.328
**Bariatric surgery**	22.0 (8.3), 32	13.2 (11.4), 29	11.8 (11.8), 24	-8.1 (10.5), 28	-8.5 (2.0); (-12.5, -4.5), p<0.001	-9.7 (2.2); (-13.9, -5.5), p<0.001		
**Monthly analgesic frequency**								
**Community weight management intervention**	14.1 (9.4), 31	10.2 (10.5), 24	9.0 (9.5), 21	..	-3.7 (2.1); (-7.9, 0.5), p=0.085	-6.0 (2.2); (-10.4, -1.6), p=0.008	-3.0 (2.6); (-8.1, 2.2), p=0.257	1.2 (2.8); (-4.3, 6.7), p=0.665
**Bariatric surgery**	10.6 (8.5), 32	7.3 (9.1), 29	9.3 (11.0), 24	..	-3.1 (2.0); (-7.0, 0.9), p=0.125	-1.2 (2.1); (-5.3, 2.9), p=0.570		
**Headache severity (VRS 0-10)**								
**Community weight management intervention**	5.0 (2.1), 31	4.0 (3.3), 24	3.8 (3.0), 21	-1.1 (2.5), 22	-1.0 (0.6); (-2.2, 0.2), p=0.110	-1.5 (0.6); (-2.7, -0.2), p=0.023	-0.9 (0.7); (-2.3, 0.6), p=0.231	0.2 (0.8); (-1.3, 1.7), p=0.796
**Bariatric surgery**	5.0 (1.9), 32	3.2 (2.6), 29	3.8 (3.1), 24	-1.6 (2.7), 28	-1.8 (0.6); (-2.9, -0.7), p=0.002	-1.2 (0.6); (-2.4, 0.0), p=0.045		

CI=confidence interval. SD=standard deviation. SE=standard error.

eTable 4. Secondary Outcome: Visual Data (Worst Eye)

	**Baseline**	**12 months**	**24 months**	**Difference baseline to 12 months**	**Difference baseline to 12 months**	**Difference baseline to 24 months**	**Difference between arms at 12 months**	**Difference between arms at 24 months**
	Mean (SD), n			Mean (SD); 95%CI, p	Hierarchical regression Mean (SE); 95%CI, p			
**logMar visual acuity**								
**Community weight management intervention**	0.0 (0.2), 33	0.0 (0.2), 28	0.0 (0.2), 21	-0.02 (0.20)	0.0 (0.1); (-0.1, 0.1), p=0.985	0.0 (0.1); (-0.1, 0.1), p=0.661	0.0 (0.1); (-0.1, 0.1), p=0.598	0.0 (0.1); (-0.1, 0.1), p=0.988
**Bariatric surgery**	0.0 (0.2), 33	0.0 (0.2), 30	-0.1 (0.1), 24	-0.08 (0.24)	-0.1 (0.1); (-0.2, 0.0), p=0.058	-0.1 (0.1); (-0.1, 0.0), p=0.120		
**Log contrast sensitivity**								
**Community weight management intervention**	1.7 (0.1), 33	1.7 (0.1), 28	1.7 (0.1), 21	..	0.0 (0.1); (0.0, 0.1), p=0.630	0.0 (0.1); (0.0, 0.1), p=0.584	0.0 (0.1); (-0.1, 0.0), p=0.411	0.0 (0.1); (-0.1, 0.1), p=0.951
**Bariatric surgery**	1.7 (0.1), 33	1.7 (0.1), 29	1.7 (0.1), 24	..	0.0 (0.1); (0.0, 0.1), p=0.463	0.1 (0.1); (0.0, 0.1), p=0.066		
**Perimetric mean deviation, dB (HVF 24-2 SITA standard)**								
**Community weight management intervention**	-3.5 (3.8), 33	-2.0 (2.3), 29	-2.1 (2.8), 22	1.2 (2.6)	1.3 (0.5); (0.3, 2.3), p=0.010	1.5 (0.6); (0.4, 2.6), p=0.010	-0.5 (0.8); (-2.0, 1.0), p=0.526	0.1 (0.8); (-1.5, 1.8), p=0.863
**Bariatric surgery**	-3.6 (3.5), 32	-2.8 (2.6), 29	-2.2 (2.2), 24	1.1 (2.7)	1.0 (0.5); (-0.1, 2.0), p=0.064	1.8 (0.6); (0.7, 2.8), p=0.002		
**Optical Coherence Tomography retinal nerve fibre layer in more affected (worst) eye (µm)**								
**Community weight management intervention**	161.7 (95.7), 32	111.8 (33.1), 28	107.4 (31.9), 22	-56 (88.3), 27	-50.5 (15.9); (-81.7, -19.3), p=0.001	-53.0 (17.1); (-86.6, -19.4), p=0.002	-8.1 (17.3); (-41.9, 25.8), p=0.641	-7.7 (19.6); (-46.1, 30.7), p=0.695
**Bariatric surgery**	148.8 (99.1), 32	103.0 (27.4), 29	103.0 (27.3), 22	-43 (107.3), 29	-45.3 (15.7); (-76.1, -14.5), p=0.004	-47.4 (17.1); (-80.9, -13.9), p=0.006		

All visual function measures are of worst eye. Negative values in the mean difference and adjusted mean difference favour surgical arm. SD=standard deviation. CI=confidence interval. HVF- Humphrey visual field.

© 2021 Mollan SP et al. *JAMA Neurology.*

eTable 5. IIH Symptoms, Baseline to 12 Months

There was no evidence of improvement in the IIH symptoms of pulsatile tinnitus, visual symptoms, diplopia and visual obscurations in either group (eTable 5).

	**Baseline**		**12 months**		**Relative Risk* (95% CI)**	**p**
	**Community weight management**	**Bariatric surgery**	**Community weight management**	**Bariatric surgery**		
**Pulsatile Tinnitus**						
Not experienced	8 (24)	9 (27)	11(38)	16(53)	0.76 (0.50 to 1.17)	0.2
Experienced	25 (76)	24 (73)	18(62)	14(47)		
**Visual Loss**						
Not experienced	10 (30)	8 (24)	15(52)	20(67)	0.69 (0.37 to 1.30)	0.2
Experienced	23 (70)	25 (76)	14(48)	10(33)		
**Diplopia**						
Not experienced	29 (88)	19 (58)	25(86)	26(87)	0.33 (0.07 to 1.67)	0.2
Experienced	4 (12)	14 (42)	4(14)	4(13)		
**Visual Obscurations**						
Not experienced	16 (48)	16 (48)	25(86)	23(77)	1.53 (0.54 to 4.35)	0.4
Experienced	17 (52)	17 (52)	4(14)	7(23)		
**Headache**						
Not experienced	1 (3)	2 (6)	6(21)	8(27)	0.98 (0.67 to 1.44)	0.9
Experienced	32 (97)	31 (94)	23(79)	22(73)		

Data are n(%) unless otherwise stated.*Adjusted for baseline IIH symptoms and acetazolamide use at entry (stratification variable). Relative risk less than 1 favours bariatric surgery.

CI = confidence intervals.

eTable 6. Quality of Life and Hospital Anxiety and Depression Scores

	Base line	12 month	24 month	Difference baseline to 12 months	Difference baseline to 24 months	Difference between arms at 12 months	Difference between arms at 24 months
	Mean (SD), n			Hierarchical regression mean (SE); 95%CI, p		Hierarchical regression mean (SE); 95%CI, p	
**Quality of Life**							
**Quality of life (SF-36) PCS summary**							
Community weight management intervention	29.1 (12.2), 30	33.4 (14.8), 25	32.0 (13.8), 22	4.5 (2.6); (-0.5, 9.6), p=0.079	4.5 (2.8); (-0.9, 9.9), p=0.099	7.3 (3.6); (0.2, 14.4), p=0.043	10.4 (3.8); (3.0, 17.9), p=0.006
Bariatric Surgery	28.3 (13.4), 30	41.6 (14.0), 28	45.2 (12.0), 23	13.1 (2.5); (8.1, 18.0), p<0.001	16.2 (2.7); (10.9, 21.5), p<0.001		
**Quality of life (SF-36) MCS summary**							
Community weight management intervention	35.8 (10.2), 30	37.9 (12.0), 25	39.6 (12.6), 22	2.1 (2.4); (-2.6, 6.9), p=0.384	4.3 (2.6); (-0.8, 9.3), p=0.097	1.6 (3.2); (-4.6, 7.8), p=0.617	-0.5 (3.4); (-7.1, 6.1), p=0.876
Bariatric Surgery	39.7 (11.5), 30	39.8 (12.5), 28	39.8 (12.4), 23	-0.1 (2.4); (-4.7, 4.6), p=0.981	0.0 (2.6); (-5.0, 5.0), p=0.998		
**Hospital anxiety and depression scores**							
**HADS -A**							
Community weight management intervention	10.5 (4.6), 32	10.5 (5.2), 26	9.7 (5.6), 21	-0.1 (0.9); (-1.8, 1.6), p=0.925	-1.2 (0.9); (-3.1, 0.6), p=0.179	-1.1 (1.3); (-3.7, 1.5), p=0.405	-0.2 (1.4); (-3.0, 2.6), p=0.887
Bariatric Surgery	10.5 (5.1), 30	9.5 (4.8), 28	9.0 (5.8), 24	-1.1 (0.9); (-2.8, 0.6), p=0.202	-1.3 (0.9); (-3.1, 0.4), p=0.142		
**HADS-D**							
Community weight management intervention	7.9 (4.8), 32	7.3 (4.2), 27	7.0 (5.0), 22	-0.3 (0.8); (-1.9, 1.3), p=0.727	-1.5 (0.9); (-3.3, 0.2), p=0.082	-1.6 (1.2); (-4.0, 0.8), p=0.200	-1.5 (1.3); (-4.0, 1.1), p=0.268
Bariatric Surgery	7.6 (4.1), 31	6.2 (5.1), 30	4.8 (4.9), 24	-1.6 (0.8); (-3.1, 0.0), p=0.053	-2.7 (0.9); (-4.4, -1.0), p=0.002		

Data are mean (SD) or mean (SE).

SD=standard deviation. SE=standard error. CI=confidence interval. HIT-6 = Headache impact test-6. HADS=Hospital anxiety and depression score. SD = standard deviation. SE = standard error. SF-36 = 36-item short form survey.

© 2021 Mollan SP et al. *JAMA Neurology.*

eTable 7. Quality of Life Subscales as Measured by the SF-36

	**Baseline**	**12 months**	**24 months**	**Difference baseline to 12 months**	**Difference baseline to 24 months**	**Difference between arms at 12 months**	**Difference between arms at 24 months**
				**Hierarchical regression mean (SE); 95%CI, p**			
**Physical functioning**							
Community weight management	56.8 (26.1), 31	62.6 (30.6), 27	59.6 (30.1), 23	4.1 (4.3); (-4.2, 12.5), p=0.331	6.6 (4.5); (-2.3, 15.5), p=0.144	20.2 (6.8); (6.9, 33.5), p=0.003	27.7 (7.2); (13.7, 41.8), p<0.001
Bariatric Surgery	56.6 (27.5), 32	81.9 (22.9), 29	92.6 (13.3), 23	24.7 (4.1); (16.6, 32.8), p<0.001	34.6 (4.5); (25.8, 43.5), p<0.001		
**Role limitation due to physical health**							
Community weight management	28.1 (40.5), 32	43.3 (47.2), 26	46.7 (43.5), 23	17.4 (8.8); (0.2, 34.6), p=0.047	24.4 (9.2); (6.4, 42.3), p=0.008		
Bariatric Surgery	36.4 (44.2), 33	56.0 (43.6), 29	57.3 (46.9), 24	9.7 (8.4); (3.2, 36.2), p=0.019	21.1 (9.0); (3.5, 38.8), p=0.019	10.5 (11.8); (-12.5, 33.6), p=0.371	5.0 (12.5); (-19.4, 29.5), p=0.687
**Role limitation due to emotional problems**							
Community weight management	37.6 (43.7), 31	51.3 (46.4), 26	53.6 (46.9), 23	13.2 (10.0); (-6.4, 32.8), p=0.186	19.2 (10.4); (-1.3, 39.6), p=0.066		
Bariatric Surgery	45.8 (45.4), 32	57.5 (47.9), 29	65.2 (44.4), 23	11.6 (9.6); (-7.2, 30.4), p=0.226	19.5 (10.4); (-0.9, 39.9), p=0.060	5.9 (12.2); (-18.0, 29.8), p=0.627	7.9 (13.1); (-17.9, 33.6), p=0.550
**Energy/Fatigue**							
Community weight management	28.0 (18.2), 32	33.7 (27.4), 27	36.1 (23.9), 23	5.7 (4.8); (-3.8, 15.1), p=0.242	10.8 (5.1); (0.7, 20.8), p=0.035		
Bariatric Surgery	26.1 (20.8), 33	49.0 (26.7), 29	46.5 (28.8), 24	22.4 (4.7); (13.2, 31.7), p<0.001	20.1 (5.0); (10.3, 30.0), p<0.001	14.9 (6.4); (2.4, 27.4), p=0.020	7.5 (6.8); (-5.9, 20.9), p=0.275
**Emotional well-being**							
Community weight management	50.5 (23.6), 32	56.0 (27.3), 27	52.7 (27.8), 23	5.3 (4.4); (-3.4, 14.0), p=0.232	5.2 (4.7); (-4.0, 14.5), p=0.268		
Bariatric Surgery	55.0 (26.3), 33	59.2 (26.0), 29	59.8 (29.4), 24	3.1 (4.3); (-5.4, 11.6), p=0.476	5.0 (4.6); (-4.0, 14.1), p=0.277	2.3 (6.9); (-11.2, 15.8), p=0.738	4.3 (7.2); (-9.9, 18.5), p=0.550
**Social functioning**							
Community weight management	46.9 (8.4), 32	48.6 (6.3), 27	50.5 (10.3), 23	1.8 (2.4); (-2.9, 6.5), p=0.450	3.6 (2.5); (-1.3, 8.5), p=0.145		
Bariatric Surgery	55.3 (11.3), 33	50.4 (10.3), 29	49.5 (8.6), 24	-4.9 (2.3); (-9.4, -0.3), p=0.036	-5.9 (2.4); (-10.7, -1.1), p=0.016	1.8 (2.5); (-3.2, 6.7), p=0.482	-1.1 (2.7); (-6.5, 4.2), p=0.680
**Pain**							

© 2021 Mollan SP et al. *JAMA Neurology.*

Community weight management	45.6 (26.2), 32	51.6 (30.4), 26	43.5 (30.0), 22	6.9 (5.9); (-4.6, 18.4), p=0.237	2.5 (6.2); (-9.7, 14.7), p=0.688		
Bariatric Surgery	42.9 (25.6), 32	62.4 (27.4), 28	61.5 (31.0), 24	18.4 (5.7); (7.2, 29.6), p=0.001	17.4 (6.1); (5.5, 29.3), p=0.004	8.4 (7.6); (-6.5, 23.3), p=0.267	11.9 (8.1); (-4.0, 27.7), p=0.143
**General health**							
Community weight management	34.1 (18.7), 32	39.4 (19.7), 27	32.8 (21.7), 23	4.7 (4.1); (-3.3, 12.7), p=0.247	0.3 (4.3); (-8.2, 8.7), p=0.949		
Bariatric Surgery	30.8 (17.6), 33	49.0 (24.0), 29	58.3 (27.8), 24	17.9 (4.0); (10.2, 25.7), p<0.001	26.4 (4.2); (18.1, 34.7), p<0.001	9.9 (5.6); (-1.2, 20.9), p=0.079	22.8 (6.0); (11.1, 34.6), p<0.001

Data are mean (SD) or mean (SE).

CI = confidence intervals. SD = standard deviation. SE = standard error. SF-36 = 36-item short form survey.

© 2021 Mollan SP et al. *JAMA Neurology.*

eTable 8. Serious Adverse Events at 12 and 24 Months

Time following randomisation	Community weight management intervention		Bariatric surgery		Total
	Related	Unrelated	Related	Unrelated	
0-12months	0	3 (3)	4	8 (6)	15
12-24months	1*	7 (4)	1	0 (0)	9
Total	1	10 (7)	5	8 (6)	24

Data are n. Those in brackets are the number of events that are a hospitalised episode of exacerbation of idiopathic intracranial hypertension.

* This participant had a headache following lumbar puncture as part of the trial, which was therefore assigned as a related serious adverse event headache. Adverse events are presented by Medical Dictionary for Regulatory Activities preferred term.

© 2021 Mollan SP et al. *JAMA Neurology.*
