# Supplementary material for: Effectiveness of Bariatric Surgery vs Community Weight Management Intervention for the Treatment of Idiopathic Intracranial Hypertension: A Randomized Clinical Trial
Source: JAMA Neurol. 2021 Apr 26;78(6):678–86. doi: 10.1001/jamaneurol.2021.0659 (PMC8077040; doi:10.1001/jamaneurol.2021.0659)
Supplement: Supplement 3. — Data Sharing Statement [file jamaneurol-e210659-s003.pdf]

Data Sharing Statement

Data

Data available: Yes
Data types: Deidentified participant data
How to access data: a.b.sinclair@bham.ac.uk
When available: beginning date: 12-01-2021, end date: 12-01-2024

Supporting Documents

Document types: None

Additional Information

Who can access the data: Individual participant data, after anonymization will be made available, along with the study protocol, statistical analysis plan and consent forms. Reasonable requests will provide data beginning 12 months and ending 3 years after publication of this article to researchers whose proposed use of the data is approved by the original study investigators. Proposals should be made to the corresponding author and requesters will need to sign a data access agreement.
Types of analyses: Reasonable requests will provide data beginning 12 months and ending 3 years after publication of this article to researchers whose proposed use of the data is approved by the original study investigators. Proposals should be made to the corresponding author and requesters will need to sign a data access agreement.
Mechanisms of data availability: Reasonable requests will provide data beginning 12 months and ending 3 years after publication of this article to researchers whose proposed use of the data is approved by the original study investigators. Proposals should be made to the corresponding author and requesters will need to sign a data access agreement.
